# Making the most of big qualitative datasets: a living systematic review of analysis methods

**DOI:** 10.3389/fdata.2024.1455399

**Published:** 2024-09-25

**Authors:** Abinaya Chandrasekar, Sigrún Eyrúnardóttir Clark, Sam Martin, Samantha Vanderslott, Elaine C. Flores, David Aceituno, Phoebe Barnett, Cecilia Vindrola-Padros, Norha Vera San Juan

**Affiliations:** ^1^Rapid Research, Evaluation, and Appraisal Lab (RREAL), Department of Targeted Intervention, University College London, London, United Kingdom; ^2^Oxford Vaccine Group, University of Oxford and NIHR Oxford Biomedical Research Centre, Oxford, United Kingdom; ^3^Centre on Climate Change and Planetary Health, The London School of Hygiene & Tropical Medicine, London, United Kingdom; ^4^Centro Latinoamericano de Excelencia en Cambio Climático y Salud, Universidad Peruana Cayetano Heredia, Lima, Peru; ^5^School of Medicine, Pontificia Universidad Católica de Chile, Santiago, Chile; ^6^Department of Clinical, Educational, and Health Psychology, University College London, London, United Kingdom

**Keywords:** big qualitative data, research methods, healthcare, digital tools, artificial intelligence, machine learning

## Abstract

**Introduction:**

Qualitative data provides deep insights into an individual's behaviors and beliefs, and the contextual factors that may shape these. Big qualitative data analysis is an emerging field that aims to identify trends and patterns in large qualitative datasets. The purpose of this review was to identify the methods used to analyse large bodies of qualitative data, their cited strengths and limitations and comparisons between manual and digital analysis approaches.

**Methods:**

A multifaceted approach has been taken to develop the review relying on academic, gray and media-based literature, using approaches such as iterative analysis, frequency analysis, text network analysis and team discussion.

**Results:**

The review identified 520 articles that detailed analysis approaches of big qualitative data. From these publications a diverse range of methods and software used for analysis were identified, with thematic analysis and basic software being most common. Studies were most commonly conducted in high-income countries, and the most common data sources were open-ended survey responses, interview transcripts, and first-person narratives.

**Discussion:**

We identified an emerging trend to expand the sources of qualitative data (e.g., using social media data, images, or videos), and develop new methods and software for analysis. As the qualitative analysis field may continue to change, it will be necessary to conduct further research to compare the utility of different big qualitative analysis methods and to develop standardized guidelines to raise awareness and support researchers in the use of more novel approaches for big qualitative analysis.

**Systematic review registration:**

https://osf.io/hbvsy/?view_only=.

## 1 Introduction

A term that has become well-known over recent years with the expansion of the digital world is “big data” which often refers to large bodies of quantitative (numerical) data (Cox et al., 2018; George et al., 2014; Hampton et al., 2013). However, this field has recently evolved to also include large sets of qualitative data (Jamieson and Lewthwaite, 2019). Forms of big qualitative data often include open-ended answers in surveys, social media data, news articles, patient health records, interview transcripts, and combinations of different data sources triangulated together (Mills, 2019).

On the one hand, qualitative data provides deep insights into an individual's behaviors and beliefs, and the contextual factors that may shape these (Grigoropoulou and Small, 2022; Johnson and Vindrola-Padros, 2017; Tenny et al., 2024). However, such data is not always harnessed to its full potential in the context of researching emergencies, or can be dismissed due to the misconception that it requires more time to collect and analyse, and has a higher risk of being more biased than quantitative data (Johnson and Vindrola-Padros, 2017; Hammarberg et al., 2016; Vindrola-Padros et al., 2020). This often leads to qualitative research focusing predominantly on small sample sizes and leaving out the possibility for large qualitative datasets. Neglecting this type of research prevents answering the “how” and “why” of research questions, as focusing on quantitative approaches only enables the “what” and “when” to be answered (Tenny et al., 2024).

On the other hand, rapid research is an approach applied when resources such as time and budgets are constrained. For instance, when responding to humanitarian crises, or when evaluating services that are already available to the public (Nunns, 2009; Vindrola-Padros, 2021). In both contexts, rapid research allows us to capture a snapshot of a situation to inform evidence-based decision making (Nunns, 2009; Vindrola-Padros, 2021).

Methods have been developed within the field of qualitative research to increase their speed. This commonly involves relying on large teams to cover more ground in a shorter amount of time, running stages of data collection and analysis in parallel, and traditionally relying largely on manual (rather than digital) methods to rapidly collect and analyse qualitative data such as group analysis, note-taking instead of full transcription, and narrowing the analytical scope to focus on specific themes (Vindrola-Padros et al., 2020; Gale et al., 2013). The field of digital qualitative data analysis has evolved in parallel, focusing on the use of computational methods and most recently artificial intelligence. However, currently no standards exist for the guidance on conducting big qualitative analysis (Karafillakis et al., 2021). Both approaches have advantages and limitations, with traditional manual analysis requiring more time, and digital analysis relying on potentially biased algorithms.

While other reviews have examined qualitative analysis methods broadly (Carrera-Fernández et al., 2014; Mohajan, 2018; Westbrook, 1994), or big data analytics (Mehta and Pandit, 2018), a comprehensive review focused specifically on methods and software for large-scale qualitative data analysis has been lacking. This represents an important gap that this systematic review is addressing, given the increased use of big qualitative data across disciplines.

The aims of this systematic review were to: (1) Identify methods used for analyzing large qualitative datasets; (2) Identify the strengths and limitations of the methods identified by authors of the literature; (3) Compare the most frequently reported methods, steps, citations, data sources, and sample sizes between studies using digital approaches and studies using manual approaches to analyse big qualitative data. The results of this review will inform the development of the collaborative and digital analysis of big qualitative data in time sensitive contexts (LISTEN) method (Clark et al., 2022).

## 2 Methods

Evidence for this review was sourced using a horizon scan which involved academic literature, gray literature, and media discourse pertaining to big qualitative data analysis methods (Amanatidou et al., 2012). Triangulating peer-reviewed and gray literature with media discourse adds depth and contextualizes results within real-time, real-world communications. Searching peer-reviewed publications, gray literature, and media discourse allowed us to comprehensively identify methods and steps being used by the wider research community to analyse big qualitative datasets.

### 2.1 Academic literature systematic review

We adhered to the Preferred Reporting Items for Systematic reviews and Meta-Analyses (PRISMA) statement (Page et al., 2021). The protocol for the systematic review was published on the Open Science Framework website (Clark et al., 2022). Adhering to the PRISMA guidelines and publishing our protocol on the Open Science Framework reflects our commitment to methodological rigor and transparency.

We designed our review to incorporate constantly updated evidence using a live systematic review (LSR) approach, which is a novel approach in evidence synthesis designed to maintain the currency and relevance of a systematic review by incorporating new research findings as they emerge. This method addresses the limitations of traditional systematic reviews, which often become outdated shortly after publication due to the constant influx of new studies. We used ResearchRabbit.ai, a peer-reviewed publication discovery tool (ResearchRabbit, 2021), to continuously source and update the systematic review with new related publications. We imported the final included publications into the ResearchRabbit.ai software as “seed publications” from which the software will identify older and newly published articles relevant to the topic of big qualitative data analysis. This process will continuously update the number of articles that fulfill the inclusion criteria and maintains the relevance of the review findings. Further information on the live systematic review can be found in Supplementary Appendix 1.

#### 2.1.1 Eligibility criteria

We included publications that described methods used to analyse big qualitative data. The “big qualitative data” was defined as studies with 100 recordings/entries or more (Brower et al., 2019; Mills, 2019). This could include ≥100 free text responses to a survey, ≥100 tweets, ≥100 interview transcripts. The types of data sources listed here are not exhaustive. Empirical peer-reviewed literature, gray literature (e.g. dissertations, conference abstracts, and conference presentations) were included in the review. We excluded studies where methods were not described for analyzing qualitative data. There were no limits on publication date, language, or the context in which the research was conducted. We did not limit the inclusion criteria by publication date to conduct this review in as comprehensive a manner as possible and because there is no precise record of when people started analyzing large qualitative datasets.

#### 2.1.2 Search, screening, and extraction

**Figure 1:**
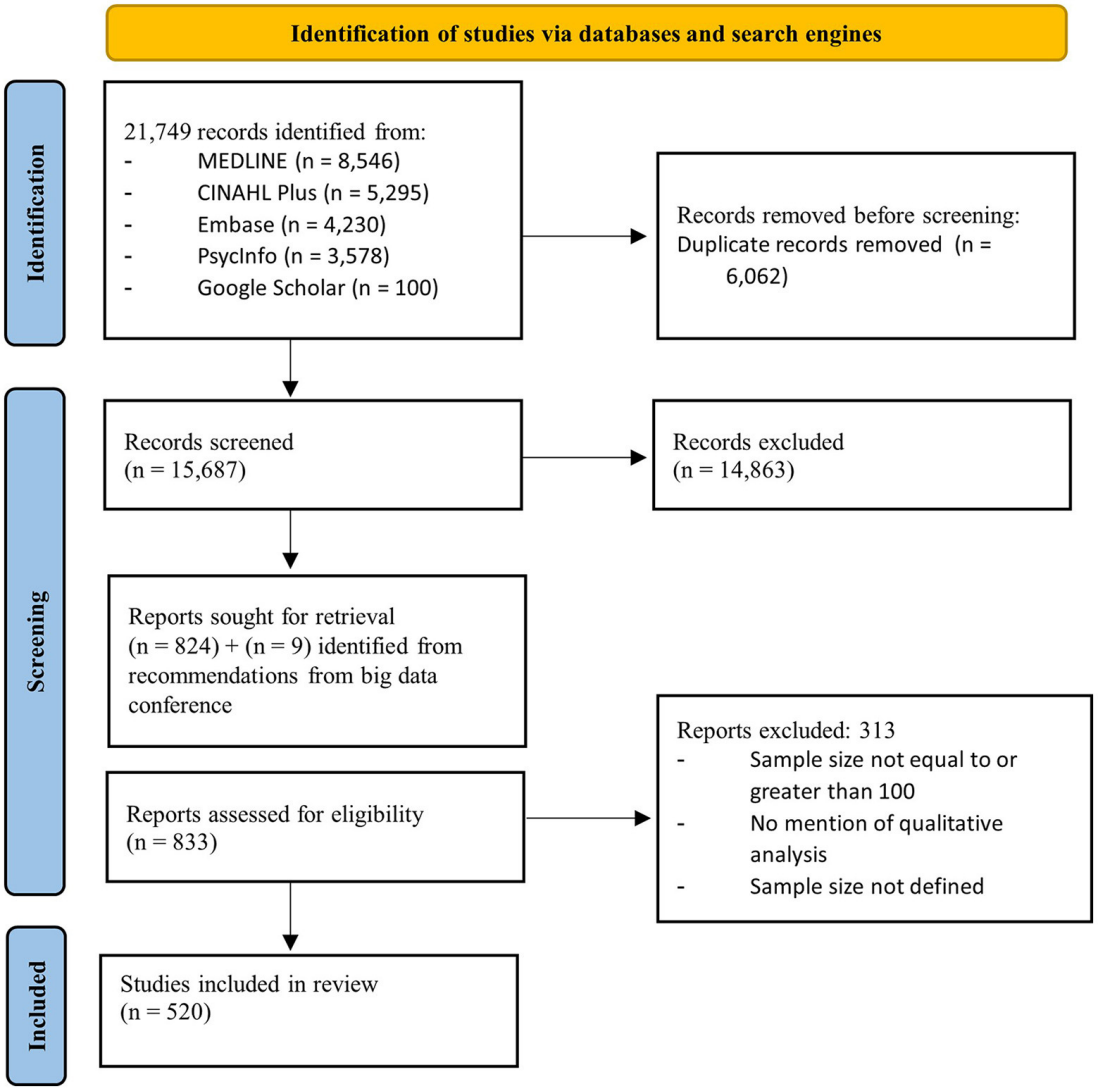
PRISMA 2020 Flow diagram of screening and inclusion process.

Four databases (Ovid MEDLINE, EBSCOhost CINAHL Plus, Ovid Embase and Ovid PsycInfo) and one search engine (Google Scholar) were searched in August 2022 using a comprehensive search strategy combining terms related to big qualitative data sources (e.g, “big data,” “large qualitative,” “big qual”), and analytical approaches (e.g., “analysis,” “methodological approach,” “interpretation”). A full strategy and search results are attached in Supplementary Appendix 2. Additional records were identified through hand searching publications and based on recommendations from our network of experts. The PRISMA Flow diagram presented in Figure 1 outlines the number of records identified from each database, number of records included after each screening stage, and reasons for excluding records from the review.

The search results were imported into EndNote to enable de-duplication, followed by the platform Rayyan (Ouzzani et al., 2016), which identified additional duplications not identified by EndNote. Two independent researchers screened titles and abstracts of identified publications. The two researchers then cross-checked 10% of each other's excluded articles. Any disagreements were discussed to reach a consensus on inclusion decisions.

The two independent researchers also split the full-text screening and combined the process with data extraction using a Microsoft Excel form with pre-specified fields. Data on study characteristics, type of analyzed data, big qualitative data analysis methods, digital software used, as well as study strengths and limitations were extracted. The data extraction fields can be found in Supplementary Appendix 3.

#### 2.1.3 Data reduction and emerging findings

Extracted data were further consolidated using Rapid Research Evaluation and Appraisal Lab (RREAL) sheets (Vindrola-Padros et al., 2022) to reduce (Watkins, 2017) down the data from the extraction form. The RREAL sheets also allowed the team to synthesize findings and identify emerging findings whilst full data extraction was ongoing. Under each category in the RREAL sheet a list of codes were then developed were and were then used to inform the development of uniform “tags” that fed into the next stage of analysis.

#### 2.1.4 Tagging

We used the tagging process to standardize data entry throughout the extraction phase in preparation for the frequency analysis and text network analysis. Key phrases were isolated using square brackets “[[ ]]” and applied consistently across all studies. The field containing extracted data on big qualitative data analysis methods was segregated into three distinct fields: analysis methods, analysis steps, and any citations of methods used. Any qualitative analysis methods and approaches discussed in the literature were categorized as “analysis methods.” Specific tasks or steps that may have formed the analysis methods or approaches such as team collaboration, open coding, or cross-checking of codes were categorized as “analysis steps.” Extraction form fields containing data on digital software usage, sample size, and data source were also tagged using square brackets. The tagging process was carried out collaboratively to ensure team members agreed on adequate removal of irrelevant text and the production of meaningful semantic units.

#### 2.1.5 Frequency analysis

The updated data extraction form, including the standardized tags was used to conduct frequency analyses within Microsoft Excel. Frequencies and percentages for analysis methods, analysis steps, digital software usage, sample size, and data sources were calculated. Frequencies by relevant sub-groups, such as different analysis methods, were also calculated.

#### 2.1.6 Text network analysis

InfraNodus, a visual text analysis tool, was used to conduct text network analysis of extracted data from the academic literature review (Paranyushkin, 2019). InfraNodus employs various algorithms for text network analysis, including text normalization, stop words removal, text-to-network conversion using bigrams and 4-g, betweenness centrality for identifying influential keywords, modularity-based community detection for topic modeling, and Force-Atlas for graph visualization. The tool analyses the graph's modularity, main cluster size, and entropy of influential words distribution to categorize discourse structure and measure bias. It also identifies structural gaps to highlight potential areas for new idea generation. Additionally, InfraNodus supports latent Dirichlet allocation (LDA) as an optional topic modeling method.

The research team used InfraNodus's algorithm to produces visual networks to show how text fragments (or tags) relate to other text fragments (or tags) within a dataset (Paranyushkin, 2011). Unique comma-separated values (CSV) files were imported into InfraNodus that include only the data fields (tags) relevant to the line of inquiry. Once the datasets were imported into InfraNodus, the software identified the tags that were most frequently referenced together with other tags (co-occurrence) within a publication. Each tag formed a node within the network, and the size of the node was proportional to how frequently that tag was referenced with other tags, otherwise known as its betweenness centrality (Paranyushkin, 2019). InfraNodus also quantified the relationships between the tags, based on how often they co-occurred together.

The inclusion of text network analysis in this study serves multiple purposes. It aligns with our aim to capture both traditional and cutting-edge methodologies in big qualitative data analysis, offering a comprehensive view of the field. This approach allows for the visualization of relationships between different analysis methods, steps, and concepts within the literature, providing unique insights into how various approaches to big qualitative data analysis are interconnected.

By using text network analysis, we were able to identify key trends and patterns in the use of different analysis methods and steps. For instance, it helped us visualize the frequent co-occurrence of “multiple coders” and “team discussion” across various analysis approaches. While our frequency analysis provided quantitative data on the prevalence of different methods and steps, text network analysis offered a more nuanced understanding of how these elements relate to each other within the context of individual studies.

Further to this, text network analysis is particularly suited to analyzing large volumes of textual data, making it highly relevant to our focus on big qualitative data analysis methods. It demonstrates one of the computational approaches that researchers might use when dealing with extensive qualitative datasets. By incorporating this method into our review, we not only study innovative methods but also employ them, providing a practical example of how such techniques can be applied in qualitative research synthesis.

### 2.2 Media discourse

#### 2.2.1 Search and extraction

A horizon scan of data from the internet was conducted on “Brandwatch” (www.brandwatch.com) a market research tool, in March 2023 (Brandwatch, 2024).

A search strategy of Boolean operatives and keywords was developed based on the terms used in the academic literature search and the team's expertise, to capture online conversations and attitudes pertaining to big qualitative data analysis methods. The Boolean search can be found in Supplementary Appendix 4 and could be grouped into the five themes below:

- “Big Qual”
- Social data
- Breadth and depth
- Digital sociology
- Method

The search was limited to the 2-year period between January 1st, 2021, and March 13th, 2023. The Brandwatch search was not limited by data source and a wide range of websites were represented including social media and news websites. All available data within the public domain was included in this media review.

Data of posts including social media and news media posts were exported to Microsoft Excel CSV files and subsequently interpreted. Graphs and diagrams were exported to JPEG and PNG files for analysis and reporting. Data on internet post volume, reach, likes, and retweets were extracted from relevant posts to evaluate their level of engagement.

#### 2.2.2 Data analysis

The social analytics algorithms (Iris) of Brandwatch were used to perform analysis on the extracted data using keywords, volume of internet posts, and sentiments where appropriate. The algorithm used by Brandwatch is names Iris, the algorithm is proprietary and not shared openly (Brandwatch, 2024). For the purpose of this study, “mentions” refers to the volume of internet results pertaining to each search. “Reach” refers to how many unique profiles or users view any particular content and “engagement” refers to the number of interactions received from different users on any particular content such as likes, comments, or retweets. Brandwatch conducts sentiment analysis by identifying and tagging keywords/phrases that have positive, negative, and neutral sentiments in extracted social media data. Brandwatch's social analytics algorithm was also used to produce time-mention volume graphs, topic wheels, topic clusters, and trending topic word clouds. Data was filtered using keywords representing the five a priori themes specified in Section 2.2.1.

## 3 Results

### 3.1 Study selection and characteristics

The findings in this section pertain to research aim 1 of this systematic review: to identify methods used for analyzing large qualitative datasets.

#### 3.1.1 Academic literature

The study selection process can be found in Figure 1. The search returned 21,749 articles and following the removal of duplicates, 15,687 articles were screened based on the relevance of their title and abstract to the eligibility criteria. There were 833 relevant articles that were then screened based on their full text. As a result of full text screening, 520 articles were deemed appropriate to include in the review, details of each article can be found in Supplementary Appendix 5. Nine of these articles were identified by the research team who recently attended a conference on the “breadth-and-depth” method (Oxford Uo, 2023).

The most common reasons for excluding publications included studies not declaring if they conducted qualitative analysis, or specifying the methods used for analysis, how large their sample sizes were, or studies having sample sizes with <100 recordings/entries.

Most included studies were conducted in high-income countries (*n* = 336, 64.6%), such as the USA (*n* = 180, 34.6%), followed by the UK (*n* = 79, 15.2%), Australia (*n* = 54, 10.4%) and Canada (*n* = 23, 4.4%). There were 65 studies that took a multi-country or global approach. The included studies collected data with sample sizes ranging from 100 to 896,867 data entries. Seven out of the eight publications with the largest sample sizes (50,000–896,867) were all using tweets as their data source for analysis.

There were 609 citations of data sources used across the publications (some publications analyzed more than one type of data source). The most common data sources were open-ended survey responses (*n* = 176, 28.90%), interview transcripts (*n* = 160, 26.27%), and first-person narratives (*n* = 119, 19.54%). Table 1 highlights the top 10 most frequent data sources, see Supplementary Appendix 6 for the complete list of data sources. Less common data sources included visual forms of data such as emojis (*n* = 1), images (from Instagram, X (formerly known as Twitter), Facebook, and websites) (*n* = 3), and videos (from Instagram and YouTube) (*n* = 4). Studies using these data sources were published between 2015–2022.

**Table 1:** The most common types of data sources and their frequency of use in the included publications.

**Data source**	**Frequency (*n*)**	**Percentage within the total 609 citations of data sources (%)**
Open-ended survey	176	28.90%
Interviews	160	26.27%
First-person narratives	120	19.70%
Focus groups	27	4.43%
Observations	15	2.46%
Tweets	13	2.13%
Social media	12	1.97%
Documents	11	1.81%
Forum messages	9	1.48%
Internet posts	8	1.31%

#### 3.1.2 Characteristics of media posts

A horizon scan of internet data yielded 37,129 mentions of “Big Qualitative Data” from the period between 1st January 2021, and 13th March, 2023, 81% of all mentions were posted on X (formerly known as Twitter). The three most popular topics of conversation across the internet were “Research,” “Social Data Science,” and “Digital Sociology” respectively. The greatest number of mentions on the internet of “Big Qualitative Data” occurred on 31st October 2022 (969 mentions), and Universities and large tech companies (Facebook, Google, and Microsoft) contributed the most to these discussions.

### 3.2 Emerging topics in big qualitative data analysis

In the following section we synthesize our findings from the academic literature and media review. We grouped results regarding (1) the most frequent methods and steps used; (2) strengths and limitations when analyzing big qualitative data; (3) comparison of digital and manual approaches to analyse large bodies of qualitative data.

#### 3.2.1 Most frequently used methods and steps to analyse big qualitative data

In this section we highlight findings from the frequency analysis, InfraNodus analysis and media review to show the most frequent methods in both the academic literature and the media.

In the academic literature, we identified over 150 different methods and steps used to analyse big qualitative data. A summary of the methods and steps are presented below, grouped by the types of approaches that can be taken to analyse big qualitative data. A full list of methods, steps, and citations is included in Supplementary Appendices 7–9.

- General qualitative analysis approaches: well-known qualitative processes were referenced which consist of a sequence of steps that enable a researcher to analyse data. These common approaches included content analysis, thematic analysis, grounded theory analysis and framework analysis. Also included were uncommon approaches such as rapid assessment procedures and the breadth-and-depth method.
- Preparing the data: several steps were focussed on preparing collected data for analysis. This included transcribing and translating data, reducing data that may have been irrelevant to the research questions, constructing a corpus of data by triangulating (bringing together) different data sources, and familiarizing with the data by re-reading extracts of the data.
- Coding: a common step used across the literature was coding. Different approaches to coding were identified, these included using inductive (identifying themes or topics from the raw data) or deductive approaches (coding to pre-defined themes or topics). Also included were collaborative approaches to coding, with multiple researchers coding the same extracts and cross checking their agreement in coding.
- Tabulations, mapping and visualizing data: approaches to visualize large data were often used, through techniques of tabulations, concept mapping, flow charts and process models. These methods allowed researchers to group together similar findings to gain a simplified overview of large datasets.
- Working with local or lived experience researchers: tailoring analysis methods based on the individuals involved in research were reported, such as using indigenist research approaches. Or using methods that enabled an in-depth understanding of each person, their beliefs, and experiences such as frame analysis, narrative inquiry, consensual qualitative research, and phenomenological approaches. Collaborating with lived experience and local researchers during the analysis and interpretation of findings was also often practiced.
- Team dynamics: many approaches were focussed on utilizing a multidisciplinary diverse team, which allowed for team discussion and for supervision or training of other team members. Techniques such as undergoing reflexive practice were often used for researchers to assess how their opinions and experiences may have biased or impacted their interpretations of their findings.
- Iterative process: iterative approaches to analysis were discussed, some studies would take this approach to start analyzing data as data collection was ongoing to re-shape data collection tools, recruitment strategies, or to gain a snapshot of the emerging findings. Other studies conducted an in-depth analysis of a sub-sample of the data, followed by an in-depth analysis of the whole sample, to get a preliminary idea of findings, or to develop a draft coding framework. Both of which could then be iteratively updated following whole sample analysis.
- Quality assurance and transparency: several techniques to assure quality and transparency throughout the analysis were identified. These included validating findings through member checking, where participants or stakeholders such as key informants could review the interpretations made by researchers and confirm whether these were correct. Following quality assurance guidelines within standard qualitative research, keeping audit trails and cross-checking other researchers' analysis and interpretations were commonly featured across the literature.
- Social media and computational analysis: using approaches such as ethnography and digital ethnography were referenced to analyse data from social media and computer-mediated interactions. Also referenced was sentiment analysis which typically involved analyzing social media data to detect positive or negative sentiment. A variety of methods that quantified qualitative findings and computational methods were also used. Some examples included: machine learning using algorithms that can learn from human analysis; natural language processing as a way to help machines understand human language; network analysis to understand the relations between participants within social structures; and statistical approaches.

The most frequently used methods and steps were identified across the literature using frequency analysis and can be found in Table 2. The most common methods that were used to guide analysis were thematic analysis, followed by content analysis and grounded theory methodology. Similarly, the most common steps that were identified were the use of multiple coders, team discussion and theme identification.

**Table 2:** The most frequent methods and steps used to analyse big qualitative datasets.

**Category**	**Frequency (*n*)**	**Percentage within the total citation of each method or step (%)**
**Big qualitative data analysis methods (*****n*** = 506 citations of hspace*5ptmethods)		
Thematic analysis	143	28.26%
Content analysis	130	25.69%
Grounded theory	43	8.50%
Consensual qualitative research	39	7.71%
Framework analysis	36	7.11%
**Steps used to analyse big qualitative data sets (*****n*** = 2,238 hspace*5ptcitations of steps)		
Multiple coders	284	12.69%
Team discussion	226	10.10%
Theme identification	161	7.19%
Inductive coding	137	6.12%
Categorizing codes	112	5.00%
Codebook	98	4.38%
Coding	96	4.29%
Cross-checking	90	4.02%
Data familiarization	88	3.93%
Iterative approach	72	3.22%

The least common methods that were used across the literature included semantic network analysis (*n* = 1), qualitative comparative analysis (*n* = 1), and topic modeling (*n* = 3). These approaches were referenced in publications that were published between 2018–2022, and only in publications that used digital software to support analysis. Topic modeling was only used in three studies with sample sizes greater than *n* = 1,000.

**Figure 2:**
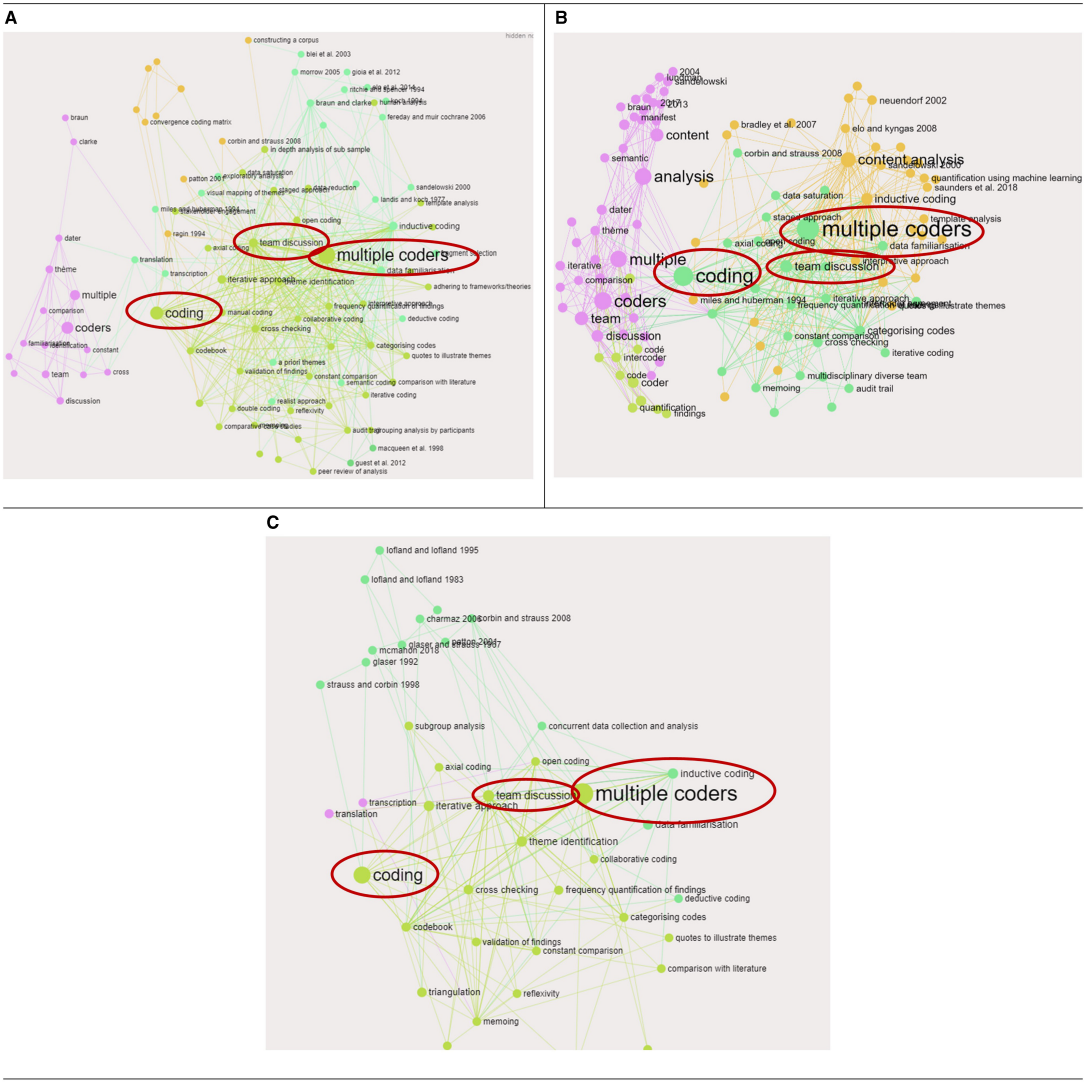
The steps for analysis that were most frequently used together within publications that specifically discussed thematic analysis, content analysis, or grounded theory analysis. **(A)** Associations with thematic analysis. **(B)** Associations with content analysis. **(C)** Associations with grounded theory analysis.

Our research team used InfraNodus to upload three separate CSV files that included publications that referenced the methods: thematic analysis, content analysis, and grounded theory analysis. Within each CSV file were also the list of citations and steps that were discussed in each publication (that had to reference either thematic analysis, content analysis or grounded theory analysis). Figure 2 shows “multiple coders” was the largest node across all three datasets, which means it was a step that was most often referenced alongside other steps in the publications, this can be seen by the large quantity of lines protruding from the node. Other large nodes that were co-occurring with other steps in each publication, include “team discussion” and “coding.” The fact that these nodes are the same color and are within proximity of each other, means they have often been mentioned together in publications. This supports the frequency analysis findings in Table 2, that the most frequent steps identified across the entire literature (e.g. multiple coders, team discussion, coding) are mentioned in publications that cite the most frequent methods too (thematic analysis, content analysis and grounded theory analysis).

InfraNodus was also used to summarize the quantitative measures of the relationships between steps, methods, and citations in this review sample. Tags in the methods, steps, and citations fields from data extraction are sorted in Table 3 by descending order of co-occurrence. The quantitative measure of co-occurrence for a tag increases when an individual tag is mentioned with other tags. The tags “multiple coders” and “team discussion” had the strongest relationship in the network with 121 co-occurrences which means these tags were used together most frequently across the publications and suggests that many studies in the sample used a team of researchers and employed collaborative analysis processes such as team discussion. “Theme identification,” “inductive coding,” and “codebook” represented the next most influential concepts in terms of co-occurrences. These findings support the frequency analysis, in that the steps that were used most frequently were also commonly referenced together.

**Table 3:** The top 15 tags with the greatest influence in the network.

**Source**	**Target**	**Frequency (*n*)**
Multiple coders	Team discussion	121
Theme identification	Multiple coders	70
Inductive coding	Multiple coders	62
Multiple coders	Codebook	58
Theme identification	Team discussion	55
Thematic analysis	Multiple coders	54
Categorizing codes	Multiple coders	51
Multiple coders	Coding	49
Multiple coders	Cross checking	49
Braun and Clarke	Thematic analysis	43
Team discussion	Codebook	42
Cross checking	Team discussion	40
Team	Discussion	39
Multiple	Coders	38
Content	Analysis	33

When looking at the findings associated with methods from the horizon scanning, there were 808 mentions from the internet from 359 unique authors that referenced the term “method.” The majority of posts that discussed methods also discussed “Data analysis” and “Data Science.” When analyzing sentiment in posts mentioning “method” and “large digital data,” ~4% (*n* = 17) of the extracted data from 414 posts—were identified as containing negative sentiments by Brandwatch.

**Figure 3:**
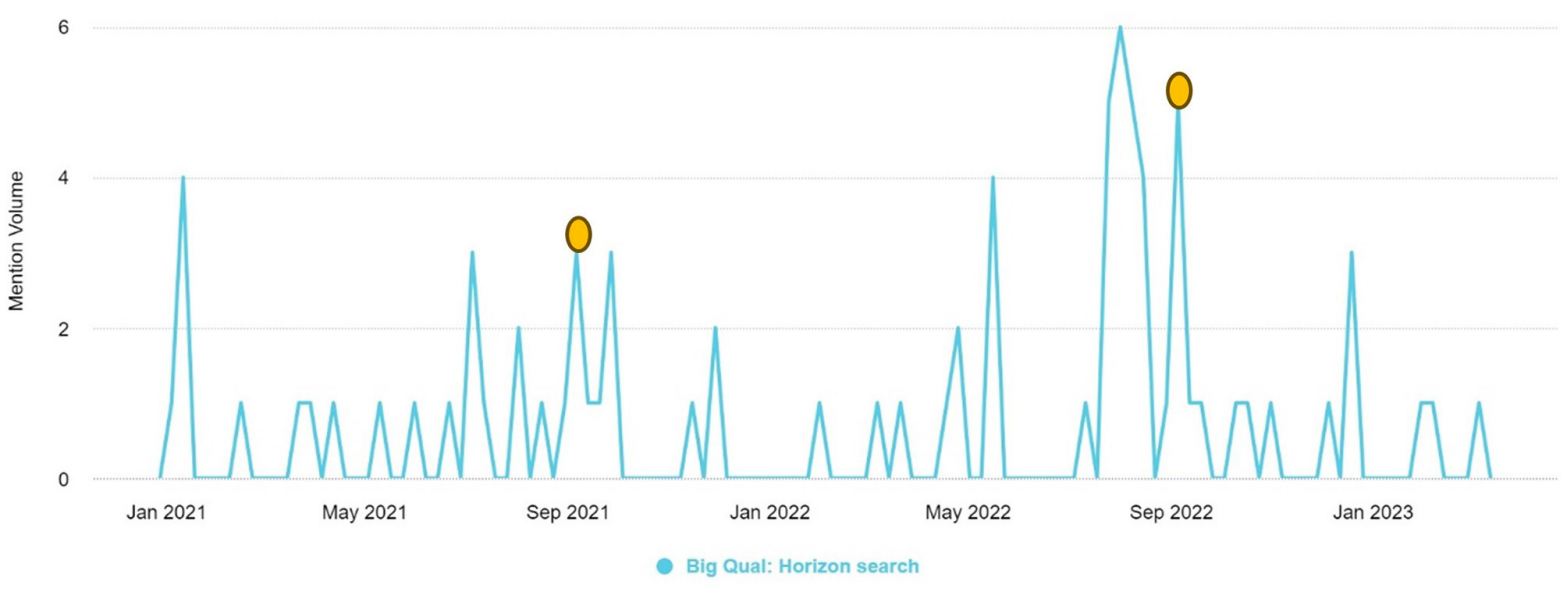
The volume of mentions of the “breadth-and-depth method” between January 2021–March 2023.

The breadth-and-depth method proposed by Edwards et al. yielded high mention volumes in the media review (Edwards et al., 2023). For example, a qualitative researcher from the University of Lincoln retweeted a post on the development of “the breadth-and-depth method of big qual analysis using a large archival qualitative dataset,” which achieved a reach of 3.9 K interactions. The top five most shared URLs pertaining to the breadth-and-depth method included links to the aforementioned publication by Edwards et al. or the associated seminar hosted by the National Center for Research Methods in July 2022 (Edwards et al., 2022). Two of the highest mention volume peaks in Figure 3 below correspond to the publication date of the paper (Edwards et al., 2023) and the sharing of the seminar recording from the National Center for Research Methods.

#### 3.2.2 Strengths and limitations when analyzing big qualitative data

Team dynamics were referred to as a strength across the literature, as individuals with different experiences and perspectives were involved in the interpretation of findings (Abraham et al., 2021). The co-design of research with local researchers and individuals with lived experience was also frequently recognized as a strength as it supported external researchers with understanding the socio-cultural dynamics that may have affected findings (Ewert, 2021). Similarly, verifying findings with stakeholders such as local researchers or individuals with lived experience or with participants of the study was also identified as a strength. Reflective practice was thought to be beneficial by researchers as it allowed them to understand how their experiences may have affected or biased their interpretations, as was team discussion as it allowed disagreements to be resolved and consensus to be reached on analysis (Hailemariam et al., 2020).

Approaches to strengthen credibility were also identified by the authors such as cross-checking analysis between researchers; and using team-based iterative approaches to develop codebooks, to get a larger pool of perspectives of what should be included in the codebook and updating the codebook as new findings emerged (Kim, 2017). Using visual outputs such as summary tables and mind maps was also recognized as a strength by authors as it enabled researchers to make sense of their large datasets within short timelines and with limited resources (Bergmann et al., 2017). Frequency counts were reported to enable researchers to identify patterns within the data based on frequency preventing researcher bias (Abraham et al., 2021).

Only a few publications discussed the strengths of software that were used to support analysis, and when they did they often reported that the software had enabled researchers to manage large volumes of data to provide an overview of the nature of the data to complement researcher-based interpretations (Abbott et al., 2017). Comparing machine learning analysis with human analysis showed a high level of agreement between the two, and the authors found that this demonstrated an element of trust in machine learning approaches (Towler et al., 2022). NVivo was identified as being beneficial as it allowed researchers to visualize and assign meaning to the data during the coding stage, it was also identified as enabling a rigorous and systematic approach. Leximancer was also recognized for enabling researchers to visualize and assign meaning to the data easily, it was also found to have a user-friendly interface. An added strength to Leximancer was that the software was found to drive the coding of data, rather than the researcher driven coding (Haynes et al., 2018).

A key limitation reported in the literature was that limited resources and timelines often prevented authors from analyzing the entirety of large datasets; from iteratively updating data collection tools based on emerging findings; from identifying when data saturation was reached; from reflecting on analysis or interpretations; from undertaking member checking; or to allow for double coding and cross-checking coding (Treves-Kagan et al., 2017). Some authors highlighted that coding and analyzing such extensive bodies of data was time intensive, labor intensive, and prone to error. Approaches such as deductive coding and template analysis were identified as preventing theory building; framework analysis was recognized as being time consuming; content analysis, rapid qualitative assessments, and concept mapping were flagged for simplifying the complexity of data and preventing researchers from understanding the detail in the breadth of findings.

Another reported limitation was that qualitative analysis was often prone to observer or researcher bias, influencing interpretations of the data (Abbott et al., 2017; Abebe et al., 2019). Authors flagged issues with the analysis of certain data sources, such as self-report surveys which often lead to short responses, preventing in-depth analysis and the ability to make interpretative claims. When analyzing global social media data, it was often hard for researchers to distinguish demographic information from participants (Abebe et al., 2019; Alpert et al., 2017). Additionally, authors flagged that these limitations often arose when social media data was excluded from analysis due to not being written in the native language of the researchers, as this limited the generalisability of findings. The authors also flagged concerns with translating data sources, as it could lead to a loss of nuance and cultural validity (Schiller, 2016; van de Beek et al., 2022).

Limitations were also reported with sentiment analysis, machine learning and semi-automated text analytics which could at times mislabel topics or fail to recognize sarcasm, leading to a required input from researchers to confirm the accuracy of the interpretations (Abraham et al., 2021). NVivo which was driven by human coding, was reported as being subjective and time consuming. Based on the social media discourse identified from the horizon scanning phase of this review, availability and affordability were the primary concerns regarding existing digital software.

#### 3.2.3 Comparison of digital and manual approaches to analyse big qualitative data

The research team relied on the frequency analysis of the academic literature to compare publications that referenced using digital software and methods to support with their analysis, vs. those that didn't. The findings from the social media discourse were also used to identify discussions on the different types of software that could be used for big qualitative data analysis.

There were 297 (57%) publications that used software to support with analysis. The sample sizes of these publications ranged from *n* = 100 to *n* = 896,867, the most common data sources were interviews, open-ended surveys, first-person narratives, focus groups and social media, whilst the most common analysis methods were thematic analysis, content analysis, and grounded theory. The publications with the largest sample sizes (50,000–896,867) throughout the whole dataset had used software.

Over 50 different types of software were used across the literature, which were grouped by the research team into the seven categories summarized below. Supplementary Appendix 10 lists all the of software that were used to analyse large sets of qualitative data.

- Basic software: the use of standard software such as Microsoft Word and variations of Microsoft Excel were frequently mentioned to display and tabulate data.
- Traditional qualitative analysis software: there were many different types of software that were used that had been developed primarily for qualitative analysis. This included software such as NVivo, Atlas.ti, Dedoose and Taguette all of which allow you to organize and code datasets.
- Text mining and text analysis software: text mining software was used across the literature to analyse vast bodies of text, often using natural language processing and machine learning to identify patterns. Leximancer is an example of a text mining software that enables analysis such as calculating the occurrence of specific words, and the identification of common words they are often associated with. Other forms of machine learning were also used in a similar way to identify patterns within the text. Treato Ltd. was a company that was referenced in some publications that could fulfill text mining capabilities. Text analysis software such as the Linguistic Inquiry and Word Count program were used to calculate the percentage of words within a text that could fall into predefined linguistic or emotional categories. Short Text Topic Modeling programs were also used to identify similar words within a body of text.
- Social network analysis software: Social network analysis allowed relationships between individuals in a network or social group to be understood. Netlytic, Node XL and Meltwater are some of the examples of software that allow for the identification of communication networks from social media data. Symplur is another example of this type of software, but especially focussed on healthcare social media.
- Statistical analysis software: Many different programs to allow for quantitative statistical analysis of transformed qualitative data were discussed. This included programming languages such as R, SAS, Python, SPSS, Stata, Matlab and others.
- Survey software: A few survey and database programs to allow for data organization were also mentioned. This included Qualtrics, REDCap, Concept System Core and Limesurvey Database.
- Miscellaneous: Other software was mentioned that didn't fall under any of the categories above but did allow for: general programming; geographical mapping; mapping of different viewpoints; identification of words and phrases; translation; tracking of publications; screen captures; online whiteboard analysis; and web-based word games.

Table 4 below lists the most common software that was used across the literature, which shows that researchers most frequently relied on traditional qualitative software (e.g., NVivo and Atlas.ti) or basic software (e.g., Microsoft Excel) to conduct analysis of their large qualitative datasets.

**Table 4:** Most frequent software used among systematic review sample.

**Digital software**	**Frequency (*n*)**	**Percentage within the total 566 citations of software**
NVivo	122	21.55%
Microsoft Excel	43	7.60%
Atlas.ti	37	6.54%
MaxQDA	21	3.71%
Microsoft Word	14	2.47%
R	12	2.12%
Dedoose	10	1.77%
SPSS	9	1.59%
NUD^*^IST	7	1.24%

Within the media review data, social media discourse on digital software for big qualitative datasets pertained to identifying software that could enable hybrid qualitative and quantitative methods, manage large data corpuses, and be suitable for collaboration during data analysis. For example, the keywords “large qualitative dataset” produced X (formally known as Twitter) discussions regarding software suggestions for analyzing big qualitative data. A University College Cork researcher tweeted a call for “good ^*^free^*^ software” recommendations that allows collaborative analysis on a large qualitative dataset. X (formally known as Twitter) users responded to this call with recommendations including Dedoose, Taguette, and Qualcoder 3.1.

There were over 223 (43%) publications that did not reference using any software to analyse their data sets. These publications had sample sizes ranging from 100–8,886 which was smaller than the studies that did rely on software. The analysis methods that were used in publications that used no software were similar to the methods used in the publications that did use digital software: content analysis, thematic analysis, and grounded theory analysis. The most common data sources with publications citing no software use were open-ended surveys, interviews, first-person narratives, focus groups, and documents. Table 5 compares these figures between publications citing software use vs. citing no software use.

**Table 5:** Comparison of sample sizes, data sources, and analysis methods in publications that used software compared to publications that did not use any software.

**Publications citing software use**	**Publications citing no software use**
**Sample size**	
100–896,867	100–8,886
Top 5 most frequent data sources (frequency of its citations hspace*5ptacross relevant publication groups)	
Interviews (93)	Open-ended survey (84)
Open-ended survey (92)	Interviews (67)
First-person narratives (70)	First-person narratives (50)
Focus groups (20)	Documents (8)
Social media (10)	Focus groups (7)
Top 5 most frequent analysis methods (frequency of its hspace*5ptcitations across relevant publication groups)	
Thematic analysis (73)	Content analysis (67)
Content analysis (58)	Thematic analysis (60)
Grounded theory (24)	Grounded theory (17)
Framework analysis (22)	Consensual qualitative research (17)
Consensual qualitative research (19)	Framework analysis (12)

## 4 Discussion

### 4.1 Main findings

Publications detailing big qualitative analysis methods were most commonly conducted in high-income countries such as the USA, UK, Australia and Canada. This is probably because low- and middle- income countries do not have access as readily to the resources and technology available to enable big qualitative analysis, or to the researchers trained in the use of these technologies, contributing to the well-known existence of inequity in access to research resources (Luna et al., 2014; Shumba and Lusambili, 2021; Wyber et al., 2015; Yegros-Yegros et al., 2020). This finding points to disparities in access to resources required for large-scale qualitative analysis. This could include monetary resources, as well as access to training. This is supported by the concerns of affordability and availability of such software, which was flagged on social media from the horizon scan.

The most common data sources used across the literature were open-ended survey responses, interview transcripts, and first-person narratives. Less common data sources were tweets, images and videos. Tweets were the most common form of data source within publications working with sample sizes >50,000. Big qualitative datasets, such as patient health records, social media posts, and data archives, have gained great prominence in healthcare research since the COVID-19 pandemic. This type of big data is readily available for analysis, and it combines the in-depth insight characteristic of qualitative inquiry and the generalisability from large sample sizes. Responding to funder initiatives, many research teams have turned to large scale secondary data analysis (Andreotta et al., 2019; Bazzaz Abkenar et al., 2021; Beneito-Montagut, 2020; Control ECfDPa, 2020). The literature in the review that analyzed forms of social media data or data that can be harnessed from online sources (internet posts, social media, YouTube comments, tweets, forum messages) were all published relatively recently, between 2010–2023. Images and videos were not used frequently, but were only referenced in literature published between 2015–2022, coinciding with previous literature that has cited this form of data source may emerge as a more common field for analysis with further enhancement of technology (Mills, 2019; Clealand and MacLeod, 2021; Cremer and Loebbecke, 2019; Glaw et al., 2017; Hitch, 2023).

Thematic analysis, content analysis, and grounded theory were the most frequently used methods in the review sample. The most frequently used steps for analysis included in the literature were the use of multiple researchers for coding, team discussion to resolve differences and theme identification. Although the “breadth and depth method” (Edwards et al., 2021) featured extensively in the social media posts reviewed in the horizon scan, only three of the 520 included studies used this method. These three studies were published between 2022–2023, which suggests its relative novelty in the field of big qualitative data analysis. A horizon scan of 37,129 media posts using BrandWatch, a commercial market research tool, identified discourse pertaining to the lack of guidance or clear directives on software availability and methodological approaches to analyzing big qualitative data sets. Other emerging fields that we identified from the literature included using analysis methods such as semantic network analysis, topic modeling, and the breadth and depth method. Topic modeling is a new and fast-growing method for qualitative data analysis utilizing machine learning techniques (Churchill and Singh, 2022). The high prevalence of mentions of the “breadth-and-depth” method which is often used with archived qualitative data and iterative computational methods, in addition to the burgeoning field of machine learning based topic modeling methods suggests a growing interest in qualitative analysis methods for large datasets. Topic modeling approaches have been used by researchers in conjunction with in-depth, manual qualitative analysis methods such as thematic analysis for large social media datasets (Rodriguez and Storer, 2020) and falls under the umbrella of the Computational social science discipline discussed frequently across the social media platforms.

Some key strengths of the approaches to analyse big qualitative data that were reported in the literature included working with teams of multi-disciplinary and diverse members, especially involving local researchers or those with lived experience in the design, conduct or verification of analysis. Other strengths included embarking on reflective practice, using team based iterative approaches, cross-checking analysis between researchers, using visual outputs to make sense of findings in short timelines, and relying on frequency counts to identify patterns. The use of digital software was considered sufficient to provide an overview of large bodies of data to complement researcher interpretations (Mills, 2019). A key limitation flagged across the literature was that limited resources and timelines had prevented researchers from being able to analyse the entirety of data sets or conduct the in-depth analysis required. Similarly, conducting in-depth analysis was flagged as being too time consuming. Additional limitations reported included relying on data sources that led to short responses prevented in-depth analysis, issues with translation of data and relying on deductive approaches that prevent theory building. Issues with relying on sentiment analysis were also identified, as software and machine learning could mischaracterise phrases, or fail to identify sarcasm. The affordability and availability of software used to conduct analysis of large sets of qualitative data was also identified as a limitation.

Although we were able to identify a plethora of research studies using large qualitative datasets, both primary and secondary, our findings suggest that researchers are still relying on traditional qualitative analysis methods such as thematic analysis, grounded theory methodology, and content analysis. Research teams are using collaborative coding processes to divide analysis efforts and overcome potential time-constraints. There are a few reasons why these approaches may not be suitable for large qualitative datasets. Brower et al. suggest that one of the challenges of analyzing large qualitative datasets with traditional methods can be the inability to create a cohesive story or analytical output (Brower et al., 2019). Newly developed methodologies such as the LISTEN method (Clark et al., 2022) and the breadth-and-depth method (Edwards et al., 2021) with a focus on collaborative discussion and conflict resolution can aid in overcoming this constraint. Both the academic literature and social media data reflected researchers' concerns over incorporating explicitly quantitative methods in their qualitative researcher studies. For instance, there is debate in the existing literature over the use of quantities to analyse text data through methods such as topic modeling, betweenness centrality, and frequency analyses. Sale et al. imply that the quantification of qualitative data is ontologically inconsistent; these methods seek to oversimplify qualitative findings and miss the nuance and contextuality of qualitative data. While studies like Nikolenko et al. emphasize the benefits of topic modeling approaches to thematically segregate and analyse large text corpuses in short periods of time, they understand the importance of refining computational models to better reflect human insight and thus support the methodological paradigm underpinning qualitative research (Nikolenko et al., 2016). This notion, reflected in studies such as Nikolenko et al.'s topic modeling efforts contribute to the growing qualitative-quantitative debate by suggesting that methodological innovation should not be limited by research paradigms and instead be responsive to scientific advancements and constraints (Gillespie et al., 2024). The use of social data analytics and digital software in this systematic review and collaborative approaches such as the LISTEN method contribute to this growing body of evidence (Paranyushkin, 2019; Brandwatch, 2024).

There were 297 (*n* = 57%) publications that used software, with over 50 different types of software reported. Most publications frequently relied on traditional qualitative software (e.g., NVivo and Atlas.ti) or basic software (e.g., Microsoft Excel) to conduct analysis of large qualitative datasets. Studies with large sample sizes tended to use more complex software such as statistical analysis packages–R, machine learning tools—AutoML, and topic modeling software—Node XL. The 223 (*n* = 43%) publications that did not use any software to support with analysis, showed few differences in the most common methods used compared to those that did use software, as both relied most frequently on thematic analysis, content analysis and grounded theory analysis. However, differences did exist with more modern approaches to analysis such as topic modeling, machine learning techniques, and social network analysis, which were only associated with studies using digital software. Additionally, the publications relying on software tended to involve larger sample sizes and had social media data as one of their most frequently used data sources.

As studies using digital software were still more likely to use traditional analysis methods, it suggests that digital software is being used to support traditional qualitative analysis approaches in a way that may overcome the time burden of manual coding procedures. The more discrete findings within publications using more modern complex software and analysis techniques with some of the largest sample sizes (*n* ≥ 500,000) or using data sources like social media, images and video may not have been used so frequently as this is a new field, but with time, we may find that these areas are used more often in qualitative research.

### 4.2 Implications of artificial intelligence and machine learning techniques in qualitative data analysis

Included studies rarely reported the ethical implications of big data and the incorporation of artificial intelligence and machine-learning approaches to overcome time-constraints and analytical cohesiveness in big qualitative data analysis. While our understanding of the ethical use of big qualitative data—specifically identifiable data such as social media posts—has advanced immensely since the Cambridge Analytica scandal of 2018 (Meredith, 2018), there is still a lack of guidelines on the analysis and reporting of findings from big qualitative datasets particularly given the growing fields of computational social science and machine-learning approaches to tackle these datasets. While this systematic review maps the breadth of big qualitative data analysis methods, innovative reporting and publishing guidelines need to be developed in this field to ensure rigor and relevance of findings.

Our review identified several AI and ML techniques being utilized in digital software for extensive qualitative data analysis. These include natural language processing, sentiment analysis, topic modeling, and semantic network analysis (van Manen, 2023; Sahin et al., 2023). Compared to traditional manual methods, AI and ML techniques offer advantages in processing speed and ability to handle extremely large datasets. For instance, topic modeling algorithms can quickly identify thematic patterns across thousands of documents, a task that would be prohibitively time-consuming for human coders (Heracleous and Fernandes, 2019; O'Kane et al., 2019). However, these techniques also have limitations. They may miss nuanced contextual meanings or struggle with sarcasm and idiomatic expressions that human analysts can readily interpret (Care and Kim, 2018; Hesse et al., 2015). Additionally, the “black box” nature of some AI algorithms can make it challenging to fully understand how conclusions are drawn (von Eschenbach, 2021; Zhang et al., 2024; Cheligeer et al., 2023).

Our findings suggest that while AI and ML techniques are increasingly being adopted, particularly for studies with very large sample sizes (*n* ≥ 500,000) or those analyzing social media data, images, and videos, traditional qualitative analysis methods still predominate. This indicates a gradual shift in the field, with researchers beginning to recognize the potential of these advanced techniques while still relying on established methodologies, perhaps due to a lack of guidelines and quality standards for the use of AI and ML techniques in qualitative research of this magnitude (O'Kane et al., 2019; Dossett et al., 2021).

The implications of this shift are multifaceted. On one hand, AI and ML techniques can significantly enhance the efficiency and scale of qualitative data analysis, potentially leading to more comprehensive insights and the ability to tackle previously unmanageable datasets. This could be particularly beneficial in time-sensitive contexts or when dealing with vast amounts of social media data. On the other hand, there are concerns about the depth of analysis these techniques can provide and their ability to capture the nuanced, context-dependent nature of qualitative data (Care and Kim, 2018; Hesse et al., 2015; Lichtenstein and Rucks-Ahidiana, 2021).

Further to this, the use of AI and ML raises important ethical considerations, particularly regarding data privacy and the potential for algorithmic bias (Zhang et al., 2024; Cheligeer et al., 2023; Akter et al., 2021). Researchers must be cautious about the uncritical application of these techniques and ensure that they complement rather than replace human interpretation and insight. Given these considerations, a hybrid approach combining AI/ML techniques for initial data exploration and pattern identification, followed by human interpretation and validation, appears to be the most robust method for analyzing extensive qualitative datasets. This approach leverages the strengths of both computational and human analysis, potentially leading to more comprehensive and nuanced findings (Care and Kim, 2018; Hesse et al., 2015). As the field continues to evolve, there is a clear need for further methodological research to refine these techniques and develop best practices for their application in qualitative research (Zhang et al., 2024; Cheligeer et al., 2023). Additionally, training and capacity building in these new methodologies will be crucial to ensure their effective and ethical use across the research community.

### 4.3 Strengths and limitations of the systematic review

Our work has several strengths. Firstly, we triangulated and combined different data sources, including online data from the media and academic literature identified from traditional databases and search engines. The media review data informed the analysis of the academic literature and provided guidance for hand searching of additional relevant literature. The range of methods that were used in this review to analyse the qualitative data included scanning the internet and identifying mentions of big qualitative data analysis methods, the RREAL sheet exercise to identify emerging findings to inform the tagging procedure for the frequency analysis, frequency analysis to identify patterns within the large bodies of data and InfraNodus to conduct text network analysis to identify co-occurring analysis steps used within publications. In addition to this multi-faceted approach to analyzing the data, the team also included multiple rounds of team discussion. This discussion was extremely useful when trying to reach consensus on the studies to include in the review when screening the data, how to align the tagging procedure, what outputs to create within the horizon scanning and InfraNodus software, and how to interpret findings. Furthermore, the live nature of this systematic review is another strength as evidence will constantly update to reflect emerging findings in the field of big qualitative data analysis methods.

Despite our thorough work, some limitations must be acknowledged. A key limitation was that due to the number of included publications, the research team did not have the capacity to conduct a quality appraisal of each publication. The wider research team decided this approach was appropriate as this review has only provided a repository of methods and approaches taken, instead of suggesting to readers that one method should be chosen over another. Whilst combining multiple methods of analysis in terms of using RREAL sheets to re-tabulate data and identify emerging findings, and conducting InfraNodus analysis increased the methodological rigor of this study, the process itself was time consuming and labor intensive. Additionally, while the tagging process increased the efficiency of the data extraction process, it is possible that reducing the data in this way could have potentially limited the nuance of our findings with respect to summarizing the reported strengths and limitations of big qualitative data analysis methods from the included publications.

### 4.4 Recommendations for future research and practice

We intend for the approaches used within this systematic review (combining academic literature and social media data, iterative approaches to data analysis, frequency analysis, text network analysis and cycles of team discussion), can guide others when working with such large bodies of qualitative data.

As qualitative data continues to grow in scale and diversity, there is room for methodological testing of hybrid computational techniques that show promise but require further validation for utility and trustworthiness. There is a need to develop standardized guidelines or methodological publications that can support researchers in navigating the available approaches and software for big qualitative data analysis. Setting standards in this field will maximize the responsible use of big qualitative data, highlighting, for example, the need to respect patient privacy, and consider stakeholder involvement alongside using Artificial Intelligence (AI) for data analysis. As the use of digital methods continues, ensuring dissemination of affordable software is essential to overcome the digital divide to enable global participation with analysis of big qualitative data.

## 5 Conclusions

This systematic review followed a novel approach to identify a diverse range of methods and software used for analyzing large qualitative datasets. Combining academic and social media discourse analysis has allowed us to develop a comprehensive overview of this developing field, and we recommend this approach to be followed by other teams conducting systematic reviews.

We identified a growing focus on larger non-traditional qualitative data sources (social media data, images and videos) and development of new methods (semantic network analysis, topic modeling, and the breadth and depth method). As this field continues to change it will be necessary to conduct further research to compare the utility of different big qualitative analysis methods. It will also be helpful to develop standardized guidelines on the approaches that can be used for big qualitative data analysis to raise awareness and support researchers in the use of more novel approaches and data sources.

Tracing this evolution of this field is a key contribution of this review, we will continue to inform on emerging evidence as part of a living systematic review (Supplementary Appendix 1). The learnings from this review are useful to our research team in consolidating the LISTEN method that uses collaborative and digital approaches to analyse large bodies of data in time sensitive contexts (Clark et al., 2022).

## Data Availability

The original contributions presented in the study are included in the article/Supplementary material, further inquiries can be directed to the corresponding author.

## Supplementary material

Supplementary Appendix 1 Information on a living systematic review.

Supplementary Appendix 2 Academic literature search terms.

Supplementary Appendix 3 Academic literature data extraction form.

Supplementary Appendix 4 Search terms to identify media data via BrandWatch.

Supplementary Appendix 5 Included articles.

Supplementary Appendix 6 List of identified data sources.

Supplementary Appendix 7 All methods used to analyse large bodies of qualitative data.

Supplementary Appendix 8 All steps used to analyse big qualitative datasets.

Supplementary Appendix 9 All citations references in the review sample.

Supplementary Appendix 10 All software used to analyse large bodies of qualitative data.

Supplementary Appendix 11 Datasheet of extracted information from included articles.
