# Supplementary material for: Making the most of big qualitative datasets: a living systematic review of analysis methods
Source: Front Big Data. 2024 Sep 25;7:1455399. doi: 10.3389/fdata.2024.1455399 (PMC11461344; doi:10.3389/fdata.2024.1455399)
Supplement: Supplementary Appendix 1 — Information on a living systematic review. [file Data_Sheet_1.DOCX]

# Appendices

## Appendix 1. Information on a living systematic review

A living systematic review has been created on Research Rabbit:

<https://www.researchrabbitapp.com/collection/public/YZ1YP7Y26Q> with the 520 included publications in this review. Associated publications that have already been published in peer-reviewed journals as well as new publications will continue to be updated on the review.

## Appendix 2. Academic literature search terms

**Ovid MEDLINE(R) ALL <1946 to November 23, 2022>**

1. breadth-and-depth method.ti,ab. 1
2. (breadth and depth method).ti,ab. 1
3. method*.ti,ab. 7767998
4. analy*.ti,ab. 7165168
5. methodological approach.ti,ab. 4521
6. analytical approach.ti,ab. 6060
7. interpret*.ti,ab. 476394
8. investigat*.ti,ab. 4157689
9. 9 3 or 4 or 5 or 6 or 7 or 8 13695886
10. large qualitative.ti,ab. 119
11. big qual.ti,ab. 1
12. large*.ti,ab. 2579489
13. big*.ti,ab. 94410
14. exp Big Data/ 2567
15. exp Qualitative Research/ 78072
16. qualitative data*.ti,ab. 21960
17. 12 or 13 or 14 2658617
18. 15 or 16 95673
19. 17 and 18 9489
20. 10 or 11 or 19 9572
21. 9 and 20 8546
22. (breadth-and-depth method or (breadth and depth method)).ti,ab. 1
23. 21 or 22 8546

**CINAHL Plus - Friday, November 25, 2022 11:17:31 AM**

| **#** | **Query** | **Limiters/Expanders** | **Last Run Via** | **Results** |
| --- | --- | --- | --- | --- |
| S1 | method* or analy* or methodological approach or analytical approach or interpret* or investigat* | Expanders - Apply equivalent subjects  Search modes - Boolean/Phrase | Interface - EBSCOhost Research Databases  Search Screen - Advanced Search  Database - CINAHL Plus | 3,361,277 |
| S2 | (large* or big* or big data*) AND (qualitative methods OR qualitative data) | Expanders - Apply equivalent subjects  Search modes - Boolean/Phrase | Interface - EBSCOhost Research Databases  Search Screen - Advanced Search  Database - CINAHL Plus | 5,165 |
| S3 | large qualitative* OR big qual* | Expanders - Apply equivalent subjects  Search modes - Boolean/Phrase | Interface - EBSCOhost Research Databases  Search Screen - Advanced Search  Database - CINAHL Plus | 606 |
| S4 | S2 OR S3 | Expanders - Apply equivalent subjects  Search modes - Boolean/Phrase | Interface - EBSCOhost Research Databases  Search Screen - Advanced Search  Database - CINAHL Plus | 5,588 |
| S5 | S1 AND S4 | Expanders - Apply equivalent subjects  Search modes - Boolean/Phrase | Interface - EBSCOhost Research Databases  Search Screen - Advanced Search  Database - CINAHL Plus | 5,277 |
| S6 | (breadth-and-depth method) or (breadth and depth method) | Expanders - Apply equivalent subjects  Search modes - Boolean/Phrase | Interface - EBSCOhost Research Databases  Search Screen - Advanced Search  Database - CINAHL Plus | 18 |
| S7 | S5 OR S6 | Expanders - Apply equivalent subjects  Search modes - Boolean/Phrase | Interface - EBSCOhost Research Databases  Search Screen - Advanced Search  Database - CINAHL Plus | 5,295 |

**APA PsycInfo <1806 to November Week 2 2022>**

1. method*.ti,ab. 1049120
2. analy*.ti,ab. 1345691
3. methodological approach.ti,ab. 3117
4. analytical approach.ti,ab. 1279
5. interpret*.ti,ab. 208309
6. investigat*.ti,ab. 776667
7. 1 or 2 or 3 or 4 or 5 or 6 2464983
8. large qualitative.ti,ab. 56
9. big qual.ti,ab. 1
10. large*.ti,ab. 441184
11. big*.ti,ab. 28385
12. exp Big Data/ 1454
13. exp Qualitative Methods/ 19962
14. qualitative data*.ti,ab. 21105
15. 10 or 11 or 12 465197
16. 13 or 14 39912
17. 15 and 16 4152
18. 8 or 9 or 17 4192
19. 7 and 18 3577
20. (breadth-and-depth method or (breadth and depth method)).ti,ab. 2
21. 19 or 20 3578

**Embase <1980 to 2022 Week 46>**

1. method*.ti,ab. 11359385
2. analy*.ti,ab. 9357533
3. methodological approach.ti,ab. 5206
4. analytical approach.ti,ab. 6436
5. interpret*.ti,ab. 556357
6. investigat*.ti,ab. 5161767
7. 1 or 2 or 3 or 4 or 5 or 6 17752156
8. large qualitative.ti,ab. 133
9. big qual.ti,ab. 0
10. large*.ti,ab. 3165313
11. big*.ti,ab. 128433
12. exp big data/ 5011
13. Qualitative Methods.ti,ab. 8375
14. qualitative data*.ti,ab. 27570
15. 10 or 11 or 12 3273076
16. 13 or 14 35465
17. 15 and 16 4387
18. 8 or 9 or 17 4500
19. 7 and 18 4228
20. (breadth-and-depth method or (breadth and depth method)).ti,ab. 2
21. 19 or 20 4230

**Google Scholar results (first 100 publications)**

(method OR analysis) AND ((big qual) OR (large qualitative)) OR (breadth and depth method)

## Appendix 3. Academic literature data extraction form

| **Study characteristics** | - Authors - Publication title - Publication year - Journal - Location of study - Study design - Sample size - Aim of study |
| --- | --- |
| **Data source** | - Type of data being analysed |
| **Qualitative analysis methods** | - Citations for methods used to analyse data - Overall methods and approaches to analyse data - Steps used to analyse data |
| **Digital software** | - Name of digital software used (if any) |
| **Strengths and limitations** | - Strengths - Limitations |

## Appendix 4. Search terms to identify media data via Brandwatch


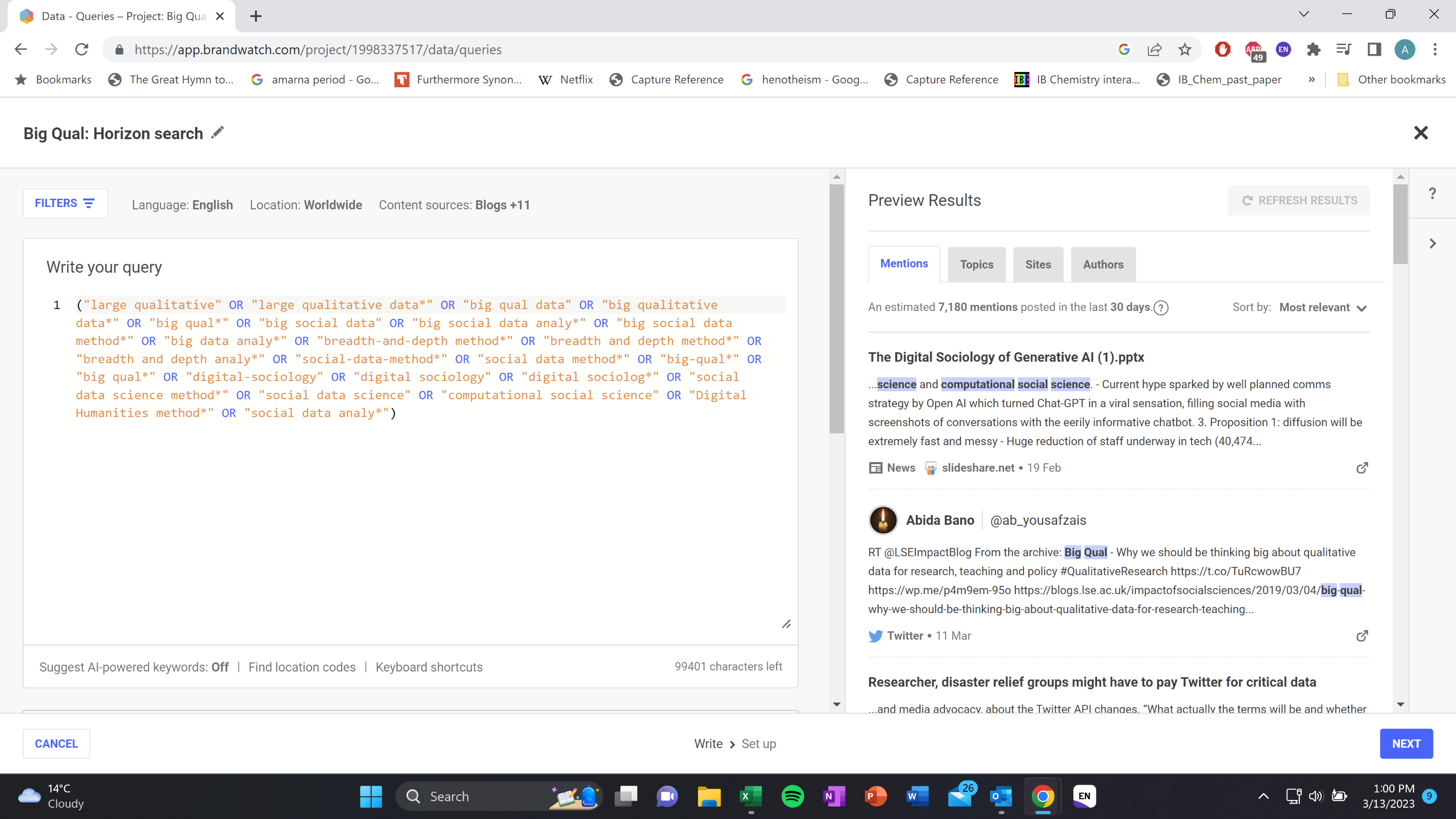


## Appendix 5. Included articles

| **Authors** | **Title** | **Publication Year** | **Journal** |
| --- | --- | --- | --- |
| Abbott, Penelope and Magin, Parker and Lujic, Sanja and Hu, Wendy | Supporting continuity of care between prison and the community for women in prison: a medical record review | 2017 | Australian health review : a publication of the Australian Hospital Association |
| Abebe, A. M. and Wudu Kassaw, M. and Zemariam, A. B. and Estifanos Shewangashaw, N. | Coverage, Opportunity, and Challenges of Expanded Program on Immunization among 12-23-Month-Old Children in Woldia Town, Northeast Ethiopia, 2018 | 2019 | BioMed Research International |
| Abraham, Traci H. and Finley, Erin P. and Drummond, Karen L. and Haro, Elizabeth K. and Hamilton, Alison B. and Townsend, James C. and Littman, Alyson J. and Hudson, Teresa | A method for developing trustworthiness and preserving richness of qualitative data during team-based analysis of large data sets | 2021 | American Journal of Evaluation |
| Adam, Katherine E. and Baillie, Sarah and Rushton, Jonathan | 'Clients. Outdoors. Animals.': retaining vets in UK farm animal practice-thematic analysis of free-text survey responses | 2019 | The Veterinary record |
| Adeniyi, Oladele Vincent and Ajayi, Anthony Idowu and Issah, Moshood and Owolabi, Eyitayo Omolara and Goon, Daniel Ter and Avramovic, Gordana and Lambert, John | Beyond health care providers' recommendations: understanding influences on infant feeding choices of women with HIV in the Eastern Cape, South Africa | 2019 | International breastfeeding journal |
| Aderinto, A. A. | Social correlates and coping measures of street-children: A comparative study of street and non-street children in south-western Nigeria | 2000 | Child Abuse and Neglect |
| Adorno, Gail and Lopez, Ellen and Burg, Mary Ann and Loerzel, Victoria and Killian, Michael and Dailey, Amy B. and Iennaco, Joanne D. and Wallace, Cara and Sharma, Dinghy Kristine B. and Stein, Kevin | Positive aspects of having had cancer: A mixed-methods analysis of responses from the American Cancer Society Study of Cancer Survivors-II (SCS-II) | 2018 | Psycho-oncology |
| Akkermans, A. Aranka and Lamerichs, J. M. W. J. Joyce and Schultz, M. J. Marcus and Cherpanath, T. G. V. Thomas and van Woensel, J. B. M. Job and van Heerde, M. Marc and van Kaam, A. H. L. C. Anton and van de Loo, M. D. Moniek and Stiggelbout, A. M. Anne and Smets, E. M. A. Ellen and de Vos, M. A. Mirjam | How doctors actually (do not) involve families in decisions to continue or discontinue life-sustaining treatment in neonatal, pediatric, and adult intensive care: A qualitative study | 2021 | Palliative medicine |
| Akram, F. and Pidcock, M. and Oake, D. and Sholler, G. and Farrar, M. and Kasparian, N. | "The Usual Challenges of Work Are All Magnified": Australian Paediatric Health Professionals' Experiences During the COVID-19 Pandemic | 2022 | Heart Lung and Circulation |
| Akter, T. and Ali, A. M. | Factors influencing knowledge and practice of hygiene in Water, Sanitation and Hygiene (WASH) programme areas of Bangladesh Rural Advancement Committee | 2014 | Rural and remote health |
| Algoso, Maricris and Ramjan, Lucie and East, Leah and Peters, Kath | An exploration of undergraduate nursing assistant employment in aged care and its value to undergraduate nursing education | 2019 | Nurse education today |
| Aljubaily, Hesham Yahya | Measuring university students' perceptions of characteristics of ideal university instructor in Saudi Arabia and the United States: An application of nonparametric item response theory study | 2011 | Dissertation Abstracts International Section A: Humanities and Social Sciences |
| Allen, Katherine R. and Goldberg, Abbie E. | Sexual activity during menstruation: A qualitative study | 2009 | Journal of Sex Research |
| Allen, Louise M. and Palermo, Claire and Hay, Margaret | Recruitment and retention of volunteer multiple mini interview interviewers: Understanding their motivations | 2022 | Medical Education |
| Alpert, Jordan M. and Dyer, Karen E. and Lafata, Jennifer Elston | Patient-centered communication in digital medical encounters | 2017 | Patient education and counseling |
| Altun, Dilek | Preschoolers' emergent motivations to learn reading: A grounded theory study | 2019 | Early Childhood Education Journal |
| Alverson, Charlotte Y. and Yamamoto, Scott H. | Messages from former students and families: Analysis of statements from one state's post-school outcomes survey | 2019 | Career Development and Transition for Exceptional Individuals |
| Amico, Jennifer R. and Stimmel, Samantha and Hudson, Shawna and Gold, Marji | "$231 ... to pull a string!!!" American IUD users' reasons for IUD self-removal: An analysis of internet forums | 2020 | Contraception |
| An, S. J. and George, A. S. and LeFevre, A. and Mpembeni, R. and Mosha, I. and Mohan, D. and Yang, A. and Chebet, J. and Lipingu, C. and Killewo, J. and Winch, P. and Baqui, A. H. and Kilewo, C. | Program synergies and social relations: implications of integrating HIV testing and counselling into maternal health care on care seeking | 2015 | BMC public health |
| Anderson, C. A. and Baker, K. and Harter, L. | Adolescents' Perceptions of Childbirth | 2020 | MCN. The American journal of maternal child nursing |
| Anderson, Ekaterina and Solch, Amanda K. and Fincke, B. Graeme and Meterko, Mark and Wormwood, Jolie B. and Vimalananda, Varsha G. | Concerns of Primary Care Clinicians Practicing in an Integrated Health System: a Qualitative Study | 2020 | Journal of general internal medicine |
| Anderson, K. and Cunningham, J. and Devitt, J. and Cass, A. | The IMPAKT study: Using qualitative research to explore the impact of end-stage kidney disease and its treatments on aboriginal and Torres Strait Islander Australians | 2013 | Kidney International Supplements |
| Anderson, K. and Diaz, A. and Parikh, D. R. and Garvey, G. | Accessibility of cancer treatment services for Indigenous Australians in the Northern Territory: perspectives of patients and care providers | 2021 | BMC health services research |
| Andre, Ernesto Barros | Origins of school conflicts in Angolan context | 2020 | Journal of Aggression, Conflict and Peace Research |
| Andreotta, Matthew and Nugroho, Robertus and Hurlstone, Mark J. and Boschetti, Fabio and Farrell, Simon and Walker, Iain and Paris, Cecile | Analyzing social media data: A mixed-methods framework combining computational and qualitative text analysis | 2019 | Behavior Research Methods |
| Aninanya, Gifty Apiung and Howard, Natasha and Williams, John E. and Apam, Benjamin and Prytherch, Helen and Loukanova, Svetla and Kamara, Eunice Karanja and Otupiri, Easmon | Can performance-based incentives improve motivation of nurses and midwives in primary facilities in northern Ghana? A quasi-experimental study | 2016 | Global health action |
| Anny Chen, L. Y. and Wu, C. Y. and Lee, M. B. and Yang, L. T. | Suicide and associated psychosocial correlates among university students in Taiwan: A mixed-methods study | 2020 | Journal of the Formosan Medical Association |
| Arevalo, Mariana and Brown, Louis D. | Using a reasoned action approach to identify determinants of organized exercise among Hispanics: a mixed-methods study | 2019 | BMC public health |
| Ariyo, Kevin and McWilliams, Andrew and David, Anthony S. and Owen, Gareth S. | Experiences of assessing mental capacity in England and Wales: A large-scale survey of professionals | 2021 | Wellcome open research |
| Armstrong, Natalie and Brewster, Liz and Tarrant, Carolyn and Dixon, Ruth and Willars, Janet and Power, Maxine and Dixon-Woods, Mary | Taking the heat or taking the temperature? A qualitative study of a large-scale exercise in seeking to measure for improvement, not blame | 2018 | Social science & medicine (1982) |
| Arnot, Megan and Wolpert, Miranda and Greenwood, Ethan | How does the British public understand mental health? A qualitative analysis of open-text responses | 2022 | The International journal of social psychiatry |
| Arrish, J. and Yeatman, H. and Williamson, M. | Self-Reported Nutrition Education Received by Australian Midwives before and after Registration | 2017 | Journal of pregnancy |
| Ashby, G. B. and Riggan, K. A. and Huang, L. and Torbenson, V. E. and Long, M. E. and Wick, M. J. and Allyse, M. A. and Rivera-Chiauzzi, E. Y. | "I had so many life-changing decisions I had to make without support": a qualitative analysis of women's pregnant and postpartum experiences during the COVID-19 pandemic | 2022 | BMC Pregnancy and Childbirth |
| Atkinson, L. and Silverio, S. A. and Bick, D. and Fallon, V. | Relationships between paternal attitudes, paternal involvement, and infant-feeding outcomes: Mixed-methods findings from a global on-line survey of English-speaking fathers | 2021 | Maternal and Child Nutrition |
| Atwood, Molly E. and Friedman, Aliza and Meisner, Brad A. and Cassin, Stephanie E. | The Exchange of Social Support on Online Bariatric Surgery Discussion Forums: A Mixed-Methods Content Analysis | 2018 | Health communication |
| Atyeo, Natalie N. and Frank, Tahvi D. and Vail, Emma F. and Sperduto, William A. L. and Boyd, David L. | Early Initiation of Breastfeeding Among Maya Mothers in the Western Highlands of Guatemala: Practices and Beliefs | 2017 | Journal of human lactation : official journal of International Lactation Consultant Association |
| Auvinen, Elina and Huhtala, Mari and Rantanen, Johanna and Feldt, Taru | Drivers or Drifters? The "Who" and "Why" of Leader Role Occupancy-A Mixed-Method Study | 2021 | Frontiers in psychology |
| Azul, A. M. and Almendra, R. and Quatorze, M. and Loureiro, A. and Reis, F. and Tavares, R. and Mota-Pinto, A. and Cunha, A. and Rama, L. and Malva, J. O. and Santana, P. and Ramalho-Santos, J. | Unhealthy lifestyles, environment, well-being and health capability in rural neighbourhoods: a community-based cross-sectional study | 2021 | BMC public health |
| Bain, A. and Kavanagh, S. and McCarthy, S. and Babar, Z. U. D. | Assessment of insulin-related knowledge among healthcare professionals in a secondary care hospital in the United Kingdom | 2019 | International Journal of Pharmacy Practice |
| Bainbridge, Daryl and Giruparajah, Mohanna and Zou, Hanyan and Seow, Hsien | The care experiences of patients who die in residential hospice: A qualitative analysis of the last three months of life from the views of bereaved caregivers | 2018 | Palliative & supportive care |
| Balchander, D. and Cabrera, C. I. and Zack, B. and Porter, S. and Sunshine, J. and D'Anza, B. | Assessing Telehealth Through the Lens of the Provider: Considerations for the Post-COVID-19 Era | 2022 | Telemedicine journal and e-health : the official journal of the American Telemedicine Association |
| Bamford, C. and Lee, R. and McLellan, E. and Poole, M. and Harrison-Dening, K. and Hughes, J. and Robinson, L. and Exley, C. | What enables good end of life care for people with dementia? A multi-method qualitative study with key stakeholders | 2018 | BMC geriatrics |
| Barber, L. and Lane, R. and Holmes, L. and Murray, N. and Hamill, J. K. | Surgical smoke: how an issue in healthcare fits a planetary health framework | 2022 | New Zealand Medical Journal |
| Barlow, Sally and Dixey, Rachael and Todd, Jacquelyne and Taylor, Vanessa and Carney, Sarah and Newell, Rob | â€˜Abandoned by Medicineâ€™? A qualitative study of women's experiences with lymphoedema secondary to cancer, and the implications for care | 2014 | Primary Health Care Research & Development |
| Barnes, Andrew and Rumbold, James L. and Olusoga, Peter | Attitudes towards protective headgear in UK rugby union players | 2017 | BMJ open sport & exercise medicine |
| Barnighausen, Kate and Matse, Sindy and Hughey, Allison B. and Hettema, Anita and Barnighausen, Till W. and McMahon, Shannon A. | "We know this will be hard at the beginning, but better in the long term": understanding PrEP uptake in the general population in Eswatini | 2020 | AIDS care |
| Battle, R. S. and Lee, J. P. and Antin, T. M. J. | Knowledge of tobacco control policies among U.S. Southeast Asians | 2010 | Journal of Immigrant & Minority Health |
| Beamish, A. J. and Rabie, M. and Johnston, M. J. and Harries, R. L. and Mohan, H. and Gokani, V. J. | Use of the eLogbook in surgical training in the United Kingdom: A nationwide survey and consensus recommendations from the Association of Surgeons in Training | 2021 | British Journal of Surgery |
| Bearman, Margaret and Greenhill, Jennene and Nestel, Debra | The power of simulation: a large-scale narrative analysis of learners' experiences | 2019 | Medical education |
| Beaton, Danielle M. and Sirois, Fuschia and Milne, Elizabeth | Experiences of criticism in adults with ADHD: A qualitative study | 2022 | PLoS ONE |
| Bell, Jennifer A. H. and Hyland, Sylvia and DePellegrin, Tania and Upshur, Ross E. G. and Bernstein, Mark and Martin, Douglas K. | SARS and hospital priority setting: a qualitative case study and evaluation | 2004 | BMC health services research |
| Bell, Sigall K. and Bourgeois, Fabienne and DesRoches, Catherine M. and Dong, Joe and Harcourt, Kendall and Liu, Stephen K. and Lowe, Elizabeth and McGaffigan, Patricia and Ngo, Long H. and Novack, Sandy A. and Ralston, James D. and Salmi, Liz and Schrandt, Suz and Sheridan, Sue and Sokol-Hessner, Lauge and Thomas, Glenda and Thomas, Eric J. | Filling a gap in safety metrics: development of a patient-centred framework to identify and categorise patient-reported breakdowns related to the diagnostic process in ambulatory care | 2022 | BMJ Quality & Safety |
| Benham-Clarke, Simon and Ford, Tamsin and Mitchell, Siobhan B. and Price, Anna and Newlove-Delgado, Tamsin and Blake, Sharon and Eke, Helen and Moore, Darren A. and Emma Russell, Abigail and Janssens, Astrid | Role of education settings in transition from child to adult health services for young people with ADHD | 2021 | Emotional & Behavioural Difficulties |
| Benjamin, L. and Ni, X. and Wang, S. W. | Implicit Support Differs Across Five Groups in the U.S., Taiwan, and Mexico | 2021 | Cultural Diversity and Ethnic Minority Psychology |
| Bennett, Michelle and von Treuer, Kathryn and McCabe, Marita P. and Beattie, Elizabeth and Karantzas, Gery and Mellor, David and Sanders, Kerrie and Busija, Ljoudmila and Goodenough, Belinda and Byers, Jessica | Resident perceptions of opportunity for communication and contribution to care planning in residential aged care | 2020 | International journal of older people nursing |
| Berkhout, Suze G. and Sheehan, Kathleen A. and Abbey, Susan E. | Individual- and Institutional-level Concerns of Health Care Workers in Canada During the COVID-19 Pandemic: A Qualitative Analysis | 2021 | JAMA network open |
| Berry, E. and Jenkins, C. and Allen, S. | Facilitators and barriers to social distancing for young people during the COVID-19 pandemic | 2022 | BMC public health |
| Beverly, E. A. and Diaz, S. and Kerr, A. M. and Balbo, J. T. and Prokopakis, K. E. and Fredricks, T. R. | Students' Perceptions of Trigger Warnings in Medical Education | 2018 | Teaching and learning in medicine |
| Bhatia, R. and Gilliam, E. and Aliberti, G. and Pinheiro, A. and Karamourtopoulos, M. and Davis, R. B. and DesRochers, L. and Schonberg, M. A. | Older adults' perspectives on primary care telemedicine during the COVID-19 pandemic | 2022 | Journal of the American Geriatrics Society |
| Bierstetel, Sabrina J. | Sharing positive experiences on social media: An investigation of online capitalization, responsiveness, and subjective well-being | 2022 | Dissertation Abstracts International: Section B: The Sciences and Engineering |
| Bigand, Teresa and Anderson, Cristina Lee and Roberts, Mary Lee and Shaw, Michele Rose and Wilson, Marian | Benefits and adverse effects of cannabis use among adults with persistent pain | 2019 | Nursing Outlook |
| Biggs, M. A. and Gould, H. and Foster, D. G. | Understanding why women seek abortions in the US | 2013 | BMC Women's Health |
| Blomberg, K. and Eriksson, M. and Boo, R. and Gronlund, A. | Using a Facebook Forum to Cope With Narcolepsy After Pandemrix Vaccination: Infodemiology Study | 2019 | Journal of medical Internet research |
| Bogart, L. M. and Fu, C. M. and Eyraud, J. and Cowgill, B. O. and Hawes-Dawson, J. and Uyeda, K. and Klein, D. J. and Elliott, M. N. and Schuster, M. A. | Evaluation of the dissemination of SNaX, a middle school-based obesity prevention intervention, within a large US school district | 2018 | Translational Behavioral Medicine |
| Boivin, J. and Harrison, C. and Mathur, R. and Burns, G. and Pericleous-Smith, A. and Gameiro, S. | Patient experiences of fertility clinic closure during the COVID-19 pandemic: appraisals, coping and emotions | 2020 | Human Reproduction |
| Bond, Virginia and Hoddinott, Graeme and Viljoen, Lario and Simuyaba, Melvin and Musheke, Maurice and Seeley, Janet | Good Health and Moral Responsibility: Key Concepts Underlying the Interpretation of Treatment as Prevention in South Africa and Zambia Before Rolling Out Universal HIV Testing and Treatment | 2016 | AIDS patient care and STDs |
| Booth, A. and Bell, T. and Halhol, S. and Pan, S. and Welch, V. and Merinopoulou, E. and Lambrelli, D. and Cox, A. | Using Social Media to Uncover Treatment Experiences and Decisions in Patients With Acute Myeloid Leukemia or Myelodysplastic Syndrome Who Are Ineligible for Intensive Chemotherapy: Patient-Centric Qualitative Data Analysis | 2019 | Journal of medical Internet research |
| Borakati, A. | Evaluation of an international medical E-learning course with natural language processing and machine learning | 2021 | BMC medical education |
| Bosakova, L. and Madarasova Geckova, A. and Borrell, C. and Hajduova, Z. and van Dijk, J. P. and Reijneveld, S. A. | How adults and children perceive the impact of social policies connected to unemployment on well-being in the household: a concept mapping approach | 2019 | International journal of public health |
| Botan, Vanessa and Laparidou, Despina and Phung, Viet-Hai and Cheung, Peter and Freeman, Adrian and Wakeford, Richard and Denney, Meiling and Law, Graham R. and Siriwardena, Aloysius Niroshan | Candidate perceptions of the UK Recorded Consultation Assessment: cross-sectional data linkage study | 2022 | Education for Primary Care |
| Bowser, D. and Sparkes, S. P. and Mitchell, A. and Bossert, T. J. and Barnighausen, T. and Gedik, G. and Atun, R. | Global Fund investments in human resources for health: innovation and missed opportunities for health systems strengthening | 2014 | Health policy and planning |
| Brents, Colleen | Occupational injuries among craft brewery workers in Colorado | 2022 | Dissertation Abstracts International: Section B: The Sciences and Engineering |
| Brimblecombe, N. and Tingle, A. and Murrells, T. | How mental health nursing can best improve service users' experiences and outcomes in inpatient settings: responses to a national consultation | 2007 | Journal of psychiatric and mental health nursing |
| Brinson, D. and Ward, C. and Ford, C. and Begg, A. | Smokefree and vapefree streets: high levels of support from tourists, residents and businesses, implications for tourist-destination communities in New Zealand | 2022 | New Zealand Medical Journal |
| Brooks, E. M. and Gonzalez, M. and Eden, A. R. and O'Neal, J. and Sabo, R. T. and Etz, R. S. | What Family Physicians Really Think of Maintenance of Certification Part II Activities | 2017 | The Journal of continuing education in the health professions |
| Brown, Suzanne and Smalling, Susan and Groza, Victor and Ryan, Scott | The experiences of gay men and lesbians in becoming and being adoptive parents | 2009 | Special Issue: Lesbian and gay adoptive parents: Issues and outcomes |
| Buck, J. and Webb, L. and Moth, L. and Morgan, L. and Barclay, S. | Persistent inequalities in Hospice at Home provision | 2020 | BMJ supportive & palliative care |
| Buehler, Emily M. and Crowley, Jenny L. and Peterson, Ashley M. and High, Andrew C. | Broadcasting for help: A typology of support-seeking strategies on Facebook | 2019 | New Media & Society |
| Byrd-Williams, C. and Ewing, M. and Rosenthal, E. L. and St John, J. A. and Menking, P. and Redondo, F. and Sieswerda, S. | Training Needs of Community Health Workers Facing the COVID-19 Pandemic in Texas: A Cross-Sectional Study | 2021 | Frontiers in public health |
| Caes, Line and Abbott, Katie and Currie, Sinead | Exploring women's perceptions of pain when breastfeeding using online forums | 2021 | International breastfeeding journal |
| Camlin, Carol S. and Charlebois, Edwin D. and Geng, Elvin and Semitala, Fred and Wallenta, Jeanna and Getahun, Monica and Kampiire, Leatitia and Bukusi, Elizabeth A. and Sang, Norton and Kwarisiima, Dalsone and Clark, Tamara D. and Petersen, Maya L. and Kamya, Moses R. and Havlir, Diane V. | Redemption of the "spoiled identity:" the role of HIV-positive individuals in HIV care cascade interventions | 2017 | Journal of the International AIDS Society |
| Camlin, Carol S. and Ssemmondo, Emmanuel and Chamie, Gabriel and El Ayadi, Alison M. and Kwarisiima, Dalsone and Sang, Norton and Kabami, Jane and Charlebois, Edwin and Petersen, Maya and Clark, Tamara D. and Bukusi, Elizabeth A. and Cohen, Craig R. and Kamya, Moses R. and Havlir, Diane | Men "missing" from population-based HIV testing: Insights from qualitative research | 2016 | AIDS Care |
| Carlsson, Tommy and Bergman, Gunnar and Karlsson, Anna-Malin and Wadensten, Barbro and Mattsson, Elisabet | Experiences of termination of pregnancy for a fetal anomaly: A qualitative study of virtual community messages | 2016 | Midwifery |
| Castro-Vazquez, Genaro | A metabolic self in contemporary Japan: A cultural reading | 2019 | Social Theory & Health |
| Catling, C. and Rossiter, C. | Midwifery workplace culture in Australia: A national survey of midwives | 2020 | Women and Birth |
| Chakraverty, Devasmita | An examination of how women and underrepresented racial/ethnic minorities experience barriers in biomedical research and medical programs | 2014 | Dissertation Abstracts International Section A: Humanities and Social Sciences |
| Chamberlain, J. M. and Shaw, K. N. and Lillis, K. A. and Mahajan, P. V. and Ruddy, R. M. and Lichenstein, R. and Olsen, C. S. and Dean, J. M. | Creating an infrastructure for safety event reporting and analysis in a multicenter pediatric emergency department network | 2013 | Pediatric Emergency Care |
| Chang, C. and Ceci, C. and Uberoi, M. and Waselewski, M. and Chang, T. | Youth Perspectives on Their Medical Team's Role in Screening for and Addressing Social Determinants of Health | 2022 | Journal of Adolescent Health |
| Chapman, Tegan and Pincombe, Jan and Harris, Mary and Fereday, Jennifer | Antenatal breast expression: Exploration and extent of teaching practices amongst International Board Certified Lactation Consultant midwives across Australia | 2013 | Women & Birth |
| Chaves, Tharcila V. and Wilffert, Bob and Sanchez, Zila M. | The use of ketamine to cope with depression and post-traumatic stress disorder: A qualitative analysis of the discourses posted on a popular online forum | 2020 | The American journal of drug and alcohol abuse |
| Cherak, Stephana J. and Brown, Allison and Kachra, Rahim and Makuk, Kira and Sudershan, Sanjana and Paget, Mike and Kassam, Aliya | Exploring the impact of the COVID-19 pandemic on medical learner wellness: a needs assessment for the development of learner wellness interventions | 2021 | Canadian medical education journal |
| Cheshire, A. and Cartwright, T. | A Population-Practice-Based Model to Understand How Yoga Impacts on Human Global Functioning: A Qualitative Study | 2021 | Journal of Alternative and Complementary Medicine |
| Chevance, Astrid and Fortel, Axel and Jouannin, Adeline and Denis, Faustine and Mamzer, Marie-France and Ravaud, Philippe and Sidorkiewicz, Stephanie | Acceptability of and Willingness to Take Digital Pills by Patients, the Public, and Health Care Professionals: Qualitative Content Analysis of a Large Online Survey | 2022 | Journal of Medical Internet Research |
| Chew, Boon-How and Cheong, Ai-Theng and Ismail, Mastura and Hamzah, Zuhra and A-Rashid, Mohd-Radzniwan and Md-Yasin, Mazapuspavina and Ali, Norsiah and Mohd-Salleh, Noridah and Bashah, Baizury | A nationwide survey of public healthcare providers' impressions of family medicine specialists in Malaysia: a qualitative analysis of written comments | 2016 | BMJ open |
| Chisolm-Straker, Makini and Jardine, Logan and Bennouna, Cyril and Morency-Brassard, Nina and Coy, Lauren and Egemba, Maria Olivia and Shearer, Peter L. | Transgender and Gender Nonconforming in Emergency Departments: A Qualitative Report of Patient Experiences | 2017 | Transgender Health |
| Clay, L. and Hay-Smith, E. J. and Treharne, G. J. and Milosavljevic, S. | Unrealistic optimism, fatalism, and risk-taking in New Zealand farmers' descriptions of quad-bike incidents: a directed qualitative content analysis | 2015 | Journal of agromedicine |
| Clise, Madeleine H. and Matthew, Susan M. and McArthur, Michelle L. | Sources of pleasure in veterinary work: A qualitative study | 2021 | The Veterinary record |
| Clough, Alan R. and Margolis, Stephen A. and Miller, Adrian and Shakeshaft, Anthony and Doran, Christopher M. and McDermott, Robyn and Sanson-Fisher, Rob and Towle, Simon and Martin, David and Ypinazar, Valmae and Robertson, Jan A. and Fitts, Michelle S. and Bird, Katrina and Honorato, Bronwyn and West, Caryn | Alcohol control policies in Indigenous communities: A qualitative study of the perceptions of their effectiveness among service providers, stakeholders and community leaders in Queensland (Australia) | 2016 | The International journal on drug policy |
| Colomer-Lahiguera, Sara and Ribi, Karin and Dunnack, Hayley J. and Cooley, Mary E. and Hammer, Marilyn J. and Miaskowski, Christine and Eicher, Manuela | Experiences of people affected by cancer during the outbreak of the COVID-19 pandemic: an exploratory qualitative analysis of public online forums | 2021 | Supportive care in cancer : official journal of the Multinational Association of Supportive Care in Cancer |
| Combes, Gill and Sein, Kim and Allen, Kerry | How does pre-dialysis education need to change? Findings from a qualitative study with staff and patients | 2017 | BMC nephrology |
| Coombe, Jacqueline and Harris, Melissa L. and Loxton, Deborah | Motivators of contraceptive method change and implications for long-acting reversible contraception (non-)use: A qualitative free-text analysis | 2019 | Sexual & reproductive healthcare : official journal of the Swedish Association of Midwives |
| Coombes, Lucy and Braybrook, Debbie and Roach, Anna and Scott, Hannah and Harardottir, Daney and Bristowe, Katherine and Ellis-Smith, Clare and Bluebond-Langner, Myra and Fraser, Lorna K. and Downing, Julia and Farsides, Bobbie and Murtagh, Fliss E. M. and Harding, Richard and C, P. O. S. | Achieving child-centred care for children and young people with life-limiting and life-threatening conditions-a qualitative interview study | 2022 | European journal of pediatrics |
| Coyne, Imelda and Sheehan, Aisling and Heery, Emily and While, Alison E. | Healthcare transition for adolescents and young adults with long-term conditions: Qualitative study of patients, parents and healthcare professionals' experiences | 2019 | Journal of clinical nursing |
| Crannell, W. Christian and Clark, Eric and Jones, Chris and James, Ted A. and Moore, Jesse | A pattern-matched Twitter analysis of US cancer-patient sentiments | 2016 | The Journal of surgical research |
| Crellin, N. E. and Priebe, S. and Morant, N. and Lewis, G. and Freemantle, N. and Johnson, S. and Horne, R. and Pinfold, V. and Kent, L. and Smith, R. and Darton, K. and Cooper, R. E. and Long, M. and Thompson, J. and Gruenwald, L. and Freudenthal, R. and Stansfeld, J. L. and Moncrieff, J. | An analysis of views about supported reduction or discontinuation of antipsychotic treatment among people with schizophrenia and other psychotic disorders | 2022 | BMC Psychiatry |
| Cresswell, K. M. and Mozaffar, H. and Lee, L. and Williams, R. and Sheikh, A. | Workarounds to hospital electronic prescribing systems: A qualitative study in English hospitals | 2017 | BMJ Quality and Safety |
| Culbert, Gabriel J. and Earnshaw, Valerie A. and Wulanyani, Ni Made Swasti and Wegman, Martin P. and Waluyo, Agung and Altice, Frederick L. | Correlates and Experiences of HIV Stigma in Prisoners Living With HIV in Indonesia: A Mixed-Method Analysis | 2015 | The Journal of the Association of Nurses in AIDS Care : JANAC |
| Curry, L. and Schwartz, H. I. and Gruman, C. and Blank, K. | Physicians' voices on physician-assisted suicide: looking beyond the numbers | 2000 | Ethics & behavior |
| Curry, Saundra E. and Cortland, Clarissa I. and Graham, Mark J. | Role-modelling in the operating room: medical student observations of exemplary behaviour | 2011 | Medical education |
| Curtis, Katherine and Liabo, Kristin and Roberts, Helen and Barker, Maggie | Consulted but not heard: a qualitative study of young people's views of their local health service | 2004 | Health expectations : an international journal of public participation in health care and health policy |
| D'Alessandro, Anthony M. and Peltier, James W. and Dahl, A. J. | A large-scale qualitative study of the potential use of social media by university students to increase awareness and support for organ donation | 2012 | Progress in transplantation (Aliso Viejo, Calif.) |
| Damush, Teresa M. and Miller, Kristine K. and Plue, Laurie and Schmid, Arlene A. and Myers, Laura and Graham, Glenn and Williams, Linda S. | National implementation of acute stroke care centers in the Veterans Health Administration (VHA): formative evaluation of the field response | 2014 | Journal of general internal medicine |
| Das, Moumita and Angeli, Federica and Krumeich, Anja J. S. M. and van Schayck, Onno C. P. | Patterns of illness disclosure among Indian slum dwellers: a qualitative study | 2018 | BMC international health and human rights |
| Das, Manoja Kumar and Arora, Narendra Kumar and Kaur, Gurkirat and Malik, Prikanksha and Kumari, Mahisha and Joshi, Shipra and Rasaily, Reeta and Chellani, Harish and Gaikwad, Harsha and Debata, Pradeep and Meena, K. R. | Perceptions of family, community and religious leaders and acceptability for minimal invasive tissue sampling to identify the cause of death in under-five deaths and stillbirths in North India: a qualitative study | 2021 | Reproductive Health |
| Davison, Colleen M. and Watt, Hayley and Michael, Saja and Bartels, Susan A. | "I don't know if we'll ever live in harmony": a mixed-methods exploration of the unmet needs of Syrian adolescent girls in protracted displacement in Lebanon | 2021 | Archives of public health = Archives belges de sante publique |
| De Gagne, Jennie C. and Cho, Eunji and Yamane, Sandra S. and Jin, Haesu and Nam, Jeehae D. and Jung, Dukyoo | Analysis of Cyberincivility in Posts by Health Professions Students: Descriptive Twitter Data Mining Study | 2021 | JMIR medical education |
| Deetjen, U. and Powell, J. A. | Informational and emotional elements in online support groups: A Bayesian approach to large-scale content analysis | 2016 | Journal of the American Medical Informatics Association |
| Dehlendorf, Christine and Kimport, Katrina and Levy, Kira and Steinauer, Jody | A qualitative analysis of approaches to contraceptive counseling | 2014 | Perspectives on Sexual and Reproductive Health |
| DeJoy, S. B. | Midwives are nice, but . . .': perceptions of midwifery and childbirth in an undergraduate class | 2010 | Journal of Midwifery & Women's Health |
| Desai, A. V. and Agarwal, R. and Epstein, A. S. and Kuperman, G. J. and Michael, C. L. and Mittelstaedt, H. and Connor, M. and Bernal, C. and Lynch, K. A. and Ostroff, J. S. and Katz, B. and Corrigan, K. L. and Kramer, D. and Davis, M. E. and Nelson, J. E. | Needs and Perspectives of Cancer Center Stakeholders for Access to Patient Values in the Electronic Health Record | 2021 | JCO oncology practice |
| Dieckmann, Nathan F. and Stoyles, Sydnee A. and Aebischer, Jonathan H. and Olvera-Alvarez, Hector A. | Dissertation Topics in Nursing | 2022 | Nursing Research |
| Digby, R. and Winton-Brown, T. and Finlayson, F. and Dobson, H. and Bucknall, T. | Hospital staff well-being during the first wave of COVID-19: Staff perspectives | 2021 | International journal of mental health nursing |
| Dillen, Kim and Joshi, Melanie and Krumm, Norbert and Hesse, Michaela and Brunsch, Holger and Schmidt, Holger and Strupp, Julia and Radbruch, Lukas and Rolke, Roman and Voltz, Raymond and Consortium, Apvel | Availability as key determinant in the palliative home care setting from the patients' and family caregivers' perspectives: A quantitative-qualitative-content analysis approach | 2021 | Palliative & supportive care |
| Dixon, S. and Herbert, D. and Loxton, D. and Lucke, J. | As many options as there are, there are just not enough for me ' : A qualitative analysis of contraceptive use and barriers to access among Australian women | 2013 | European Journal of Contraception and Reproductive Health Care |
| Doldor, Elena and Wyatt, Madeleine and Silvester, Jo | Statesmen or cheerleaders? Using topic modeling to examine gendered messages in narrative developmental feedback for leaders | 2019 | The Leadership Quarterly |
| Dollahite, David C. and Marks, Loren D. and Barrow, Betsy Hughes | Exploring relational reconciliation processes in Christian, Jewish, and Muslim families | 2019 | Family Relations: An Interdisciplinary Journal of Applied Family Studies |
| Donaldson-Feilder, Emma and Lewis, Rachel and Pavey, Louisa and Jones, Bethan and Green, Melanie and Webster, Angela | Perceived barriers and facilitators of exercise and healthy dietary choices: A study of employees and managers within a large transport organisation | 2017 | Health Education Journal |
| Donnelly, Catherine and Ashcroft, Rachelle and Mofina, Amanda and Bobbette, Nicole and Mulder, Carol | Measuring the performance of interprofessional primary health care teams: understanding the teams perspective | 2019 | Primary health care research & development |
| Downe, S. and Kingdon, C. and Kennedy, R. and Norwell, H. and McLaughlin, M. J. and Heazell, A. E. P. | Post-mortem examination after stillbirth: Views of UK-based practitioners | 2012 | European Journal of Obstetrics and Gynecology and Reproductive Biology |
| Drummond, Claire and Sheppard, Lorraine | Examining primary and secondary school canteens and their place within the school system: a South Australian study | 2011 | Health education research |
| DuBow, Wendy M. and Kaminsky, Alexis | How an online women in technology group provides a locus of opposition | 2019 | Computers in Human Behavior |
| Dunford, Benjamin B. and Perrigino, Matthew and Tucker, Sharon J. and Gaston, Cynthia L. and Young, Jim and Vermace, Beverly J. and Walroth, Todd A. and Buening, Natalie R. and Skillman, Katherine L. and Berndt, Dawn | Organizational, Cultural, and Psychological Determinants of Smart Infusion Pump Work Arounds: A Study of 3 U.S. Health Systems | 2017 | Journal of Patient Safety |
| Edwards, A. and Thomas, R. and Williams, R. and Ellner, A. L. and Brown, P. and Elwyn, G. | Presenting risk information to people with diabetes: Evaluating effects and preferences for different formats by a web-based randomised controlled trial | 2006 | Patient Education and Counseling |
| Eide, Wenche Mjanger and Johansson, Linda and Eide, Leslie Sp | FIRST-YEAR nursing students' experiences of simulation involving care of older patients. A descriptive and exploratory study | 2020 | Nurse education in practice |
| Ekberg, Stuart and Barnes, Rebecca K. and Kessler, David S. and Mirza, Selman and Montgomery, Alan A. and Malpass, Alice and Shaw, Alison R. G. | Relationship between expectation management and client retention in online cognitive behavioural therapy | 2015 | Behavioural and Cognitive Psychotherapy |
| Ellinas, Elizabeth H. and Kaljo, Kristina and Patitucci, Teresa N. and Novalija, Jutta and Byars-Winston, Angela and Fouad, Nadya A. | No Room to "Lean In": A Qualitative Study on Gendered Barriers to Promotion and Leadership | 2019 | Journal of women's health (2002) |
| Elwy, A. Rani and Maguire, Elizabeth M. and Gallagher, Thomas H. and Asch, Steven M. and Durfee, Janet M. and Martinello, Richard A. and Bokhour, Barbara G. and Gifford, Allen L. and Taylor, Thomas J. and Wagner, Todd H. | Risk Communication After Health Care Exposures: An Experimental Vignette Survey With Patients | 2021 | MDM policy & practice |
| Emma Hilton, Charlotte | Unveiling self-harm behaviour: what can social media site Twitter tell us about self-harm? A qualitative exploration | 2017 | Journal of clinical nursing |
| Enlund, K. B. and Pettersson, A. and Eldh, A. C. | Dog Owners' Ideas and Strategies Regarding Dental Health in Their Dogs-Thematic Analysis of Free Text Survey Responses | 2022 | Frontiers in Veterinary Science |
| Ewert, Rebecca | "A country boy can survive:" Rural culture and male-targeted suicide prevention messaging | 2021 | Social science & medicine (1982) |
| Farmer, Didi Bertrand and Berman, Leslie and Ryan, Grace and Habumugisha, Lameck and Basinga, Paulin and Nutt, Cameron and Kamali, Francois and Ngizwenayo, Elias and St Fleur, Jacklin and Niyigena, Peter and Ngabo, Fidele and Farmer, Paul E. and Rich, Michael L. | Motivations and Constraints to Family Planning: A Qualitative Study in Rwanda's Southern Kayonza District | 2015 | Global health, science and practice |
| Farmer, Jane and Bigby, Christine and Davis, Hilary and Carlisle, Karen and Kenny, Amanda and Huysmans, Richard | The state of health services partnering with consumers: evidence from an online survey of Australian health services | 2018 | BMC Health Services Research |
| Farnood, Annabel and Johnston, Bridget and Mair, Frances S. | An analysis of the diagnostic accuracy and peer-to-peer health information provided on online health forums for heart failure | 2022 | Journal of advanced nursing |
| Feeley, T. and Ffrench-O'Carroll, R. and Tan, M. H. and Magner, C. and L'Estrange, K. and O'Rathallaigh, E. and Whelan, S. and Lyons, B. and O'Connor, E. | A model for occupational stress amongst paediatric and adult critical care staff during COVID-19 pandemic | 2021 | International archives of occupational and environmental health |
| Fehr, A. and Nieto-Sanchez, C. and Muela, J. and Jaiteh, F. and Ceesay, O. and Maneh, E. and Baldeh, D. and Achan, J. and Dabira, E. and Conteh, B. and Bunders-Aelen, J. and Smekens, T. and Broekhuizen, H. and D'Alessandro, U. and Peeters Grietens, K. | From informed consent to adherence: factors influencing involvement in mass drug administration with ivermectin for malaria elimination in The Gambia | 2021 | Malaria Journal |
| Feler, Joshua and Tan, Amy and Sammann, Amanda and Matouk, Charles and Hwang, David Y. | Decision Making Among Patients with Unruptured Aneurysms: A Qualitative Analysis of Online Patient Forum Discussions | 2019 | World neurosurgery |
| Ferguson, S. L. and Pitt, C. and Pitt, L. | Using artificial intelligence to examine online patient reviews | 2021 | Journal of health psychology |
| Fisher, C. and Herbenick, D. and Reece, M. and Dodge, B. and Satinsky, S. and Fischtein, D. | Exploring sexuality education opportunities at in-home sex-toy parties in the United States | 2010 | Sex Education |
| Fitch, M. I. and Longo, C. J. and Chan, R. J. | Cancer patients' perspectives on financial burden in a universal healthcare system: Analysis of qualitative data from participants from 20 provincial cancer centers in Canada | 2021 | Patient Education and Counseling |
| Fleming, Crystal M. and Lamont, Michele and Welburn, Jessica S. | African Americans respond to stigmatization: The meanings and salience of confronting, deflecting conflict, educating the ignorant and 'managing the self' | 2012 | Ethnic and Racial Studies |
| Foldy, Erica Gabrielle | Something of collaborative manufacture: The construction of race and gender identities in organizations | 2012 | Journal of Applied Behavioral Science |
| Foli, Karen J. and Reddick, Blake and Zhang, Lingsong and Krcelich, Kathryn | Substance Use in Registered Nurses: "I Heard About a Nurse Who . . ." | 2020 | Journal of the American Psychiatric Nurses Association |
| Forero, R. and Nahidi, S. and De Costa, J. and Mohsin, M. and Fitzgerald, G. and Gibson, N. and McCarthy, S. and Aboagye-Sarfo, P. | Application of four-dimension criteria to assess rigour of qualitative research in emergency medicine | 2018 | BMC health services research |
| Fowler, Cathrine and Schmied, Virginia and Psaila, Kim and Kruske, Sue and Rossiter, Chris | Ready for practice: What child and family health nurses say about education | 2015 | Nurse Education Today |
| Freeman, Emily and Coast, Ernestina and Murray, Susan F. | Men's Roles in Women's Abortion Trajectories in Urban Zambia | 2017 | International perspectives on sexual and reproductive health |
| Friend, Duncan S. | Data, dependence, democracy: Influence in the secondary use of government information | 2021 | Dissertation Abstracts International: Section B: The Sciences and Engineering |
| Frisch, Noreen and Butcher, Howard K. and Campbell, Diana and Weir-Hughes, Dickon | Holistic Nurses' Use of Energy-Based Caring Modalities | 2018 | Journal of holistic nursing : official journal of the American Holistic Nurses' Association |
| Froh, E. B. and Spatz, D. L. | Navigating Return to Work and Breastfeeding in a Hospital with a Comprehensive Employee Lactation Program | 2016 | Journal of human lactation : official journal of International Lactation Consultant Association |
| Fukuda, Tetsuya | School belonging and L2 motivation of first-year students at four Japanese universities | 2021 | Dissertation Abstracts International Section A: Humanities and Social Sciences |
| Fulop, Naomi J. and Ramsay, Angus I. G. and Perry, Catherine and Boaden, Ruth J. and McKevitt, Christopher and Rudd, Anthony G. and Turner, Simon J. and Tyrrell, Pippa J. and Wolfe, Charles D. A. and Morris, Stephen | Explaining outcomes in major system change: a qualitative study of implementing centralised acute stroke services in two large metropolitan regions in England | 2016 | Implementation science : IS |
| Furber, C. and Bedwell, C. and Campbell, M. and Cork, M. and Jones, C. and Rowland, L. and Lavender, T. | The Challenges and Realties of Diaper Area Cleansing for Parents | 2012 | JOGNN - Journal of Obstetric, Gynecologic, and Neonatal Nursing |
| Gadbois, Emily A. and Tyler, Denise A. and Shield, Renee R. and McHugh, John P. and Winblad, Ulrika and Trivedi, Amal and Mor, Vincent | Medicare Advantage control of postacute costs: perspectives from stakeholders | 2018 | The American journal of managed care |
| Gallay, Erin and Furlan Brighente, Miriam and Flanagan, Constance and Lowenstein, Ethan | Placeâ€based civic scienceâ€”collective environmental action and solidarity for ecoâ€resilience | 2022 | Child & Adolescent Mental Health |
| Garvey, Gail and Anderson, Kate and Gall, Alana and Butler, Tamara L. and Cunningham, Joan and Whop, Lisa J. and Dickson, Michelle and Ratcliffe, Julie and Cass, Alan and Tong, Allison and Arley, Brian and Howard, Kirsten | What Matters 2 Adults (WM2Adults): Understanding the Foundations of Aboriginal and Torres Strait Islander Wellbeing | 2021 | International journal of environmental research and public health |
| Geller, S. E. and Cox, S. M. and Kilpatrick, S. J. | A descriptive model of preventability in maternal morbidity and mortality | 2006 | Journal of Perinatology |
| Gergel, Tania and Das, Preety and Owen, Gareth and Stephenson, Lucy and Rifkin, Larry and Hindley, Guy and Dawson, John and Ruck Keene, Alex | Reasons for endorsing or rejecting self-binding directives in bipolar disorder: a qualitative study of survey responses from UK service users | 2021 | The lancet. Psychiatry |
| Germano, Elaine and Schorn, Mavis N. and Phillippi, Julia C. and Schuiling, Kerri | Factors that Influence Midwives to Serve as Preceptors: An American College of Nurse-Midwives Survey | 2014 | Journal of Midwifery & Women's Health |
| Giles, T. M. and Hammad, K. and Breaden, K. and Drummond, C. and Bradley, S. L. and Gerace, A. and Muir-Cochrane, E. | Nurses' perceptions and experiences of caring for patients who die in the emergency department setting | 2019 | International emergency nursing |
| Gold, Jessica A. and Jia, Lena and Bentzley, Jessica P. and Bonnet, Kacy A. and Franciscus, Amanda M. and Denduluri, Meenakshi S. and Zappert, Laraine T. | WISE: A support group for graduate and post-graduate women in STEM | 2021 | International Journal of Group Psychotherapy |
| Gonzalez, G. and Vaculik, K. and Khalil, C. and Zektser, Y. and Arnold, C. and Almario, C. V. and Spiegel, B. M. R. and Anger, J. T. | Women's Experience with Stress Urinary Incontinence: Insights from Social Media Analytics | 2020 | Journal of Urology |
| Gonzalez, Gabriela and Vaculik, Kristina and Khalil, Carine and Zektser, Yuliya and Arnold, Corey and Almario, Christopher V. and Spiegel, Brennan M. R. and Anger, Jennifer T. | Using Digital Ethnography to Understand the Experience of Women With Pelvic Organ Prolapse | 2021 | Female pelvic medicine & reconstructive surgery |
| Gonzalez, G. and Vaculik, K. and Khalil, C. and Zektser, Y. and Arnold, C. W. and Almario, C. V. and Spiegel, B. M. R. and Anger, J. T. | Experiences of Women with Interstitial Cystitis/Bladder Pain Syndrome: What Can We Learn From Women's Online Discussions? | 2022 | The Journal of urology |
| Gonzalez-Zapata, L. I. and AlvarezDardet, C. and Millstone, E. and Clemente-Gomez, V. and Holdsworth, M. and Ortiz-Moncada, R. and Lobstein, T. and Sarri, K. and De Marchi, B. and Horvath, K. Z. | The potential role of taxes and subsidies on food in the prevention of obesity in Europe | 2010 | Journal of Epidemiology and Community Health |
| Green, Carla A. and Polen, Michael R. and Janoff, Shannon L. and Castleton, David K. and Perrin, Nancy A. | "Not getting tanked": definitions of moderate drinking and their health implications | 2007 | Drug and alcohol dependence |
| Green, Raquel and Delfabbro, Paul H. and King, Daniel L. | Player-avatar interactions in habitual and problematic gaming: A qualitative investigation | 2021 | Journal of behavioral addictions |
| Greene, Jessica and Farley, Diane C. and Christianson, Jon B. and Scanlon, Dennis P. and Yunfeng, Shi | From Rhetoric to Reality: Consumer Engagement in 16 Multi-Stakeholder Alliances | 2016 | American Journal of Managed Care |
| Griffith, Frances J. and Wong, Serena and Dietrich, Kelsey M. and Exline, Julie J. and Pargament, Kenneth I. | The Music was Speaking to Me': Using narrative inquiry to describe sacred moments with music | 2022 | The Arts in Psychotherapy |
| Griffiths, Austin and Desrosiers, Patricia and Gabbard, Jay and Royse, David and Piescher, Kristine | Retention of Child Welfare Caseworkers: The Wisdom of Supervisors | 2019 | Child Welfare |
| Gyongy, Kinga | The relative emphasis of play rules between experienced and trainee caregivers of toddlers | 2017 | Early Child Development and Care |
| Hadjigeorgiou, E. and Vogazianos, P. and Christofi, M. D. and Motrico, E. and Dominguez-Salas, S. and Mesquita, A. R. and Christoforou, A. | Experiences, concerns, and needs of pregnant and postpartum women during the Covid-19 pandemic in Cyprus: a cross-sectional study | 2022 | BMC Pregnancy and Childbirth |
| Haesler, E. and Pittman, J. and Cuddigan, J. and Law, S. and Chang, Y. Y. and Balzer, K. and Berlowitz, D. and Carville, K. and Kottner, J. and Litchford, M. and Moore, Z. and Mitchell, P. and Sigaudo-Roussel, D. | An exploration of the perspectives of individuals and their caregivers on pressure ulcer/injury prevention and management to inform the development of a clinical guideline | 2022 | Journal of Tissue Viability |
| Hagaman, Ashley K. and Wutich, Amber | How many interviews are enough to identify metathemes in multisited and cross-cultural research? Another perspective on Guest, Bunce, and Johnson's (2006) landmark study | 2017 | Field Methods |
| Hailemariam, Maji and Key, Kent and Jefferson, Bernadel L. and Muhammud, Janice and Johnson, Jennifer E. | Community-Based Participatory Qualitative Research for Women: Lessons from the Flint Women's Study | 2020 | Progress in community health partnerships : research, education, and action |
| Halkett, G. K. B. and Berg, M. and Ebert, M. A. and Cutt, D. and Davis, M. and Hegney, D. and House, M. and Judson, M. and Kearvell, R. and Krawiec, M. and Lester, L. and Maresse, S. and McLoone, P. and McKay, J. | Radiation therapists' perspectives on participating in research | 2017 | Journal of Medical Radiation Sciences |
| Hamblin, Lydia E. and Essenmacher, Lynnette and Upfal, Mark J. and Russell, Jim and Luborsky, Mark and Ager, Joel and Arnetz, Judith E. | Catalysts of worker-to-worker violence and incivility in hospitals | 2015 | Journal of Clinical Nursing (John Wiley & Sons, Inc.) |
| Hanlon, Holly Rose and Prihodova, Lucia and Russell, Thelma and Donegan, Deirdre and O'Shaughnessy, Ann and Hoey, Hilary | Doctors' engagement with a formal system of continuing professional development in Ireland: a qualitative study in perceived benefits, barriers and potential improvements | 2021 | BMJ open |
| Hardcastle, Sarah J. and Chan, Derwin C. K. and Caudwell, Kim M. and Sultan, Sarwat and Cranwell, Jo and Chatzisarantis, Nikos L. D. and Hagger, Martin S. | Larger and More Prominent Graphic Health Warnings on Plain-Packaged Tobacco Products and Avoidant Responses in Current Smokers: a Qualitative Study | 2016 | International journal of behavioral medicine |
| Harder, Helena and Starkings, Rachel M. L. and Fallowfield, Lesley J. and Menon, Usha and Jacobs, Ian J. and Jenkins, Valerie A. and trialists, Ukctocs | Sexual functioning in 4,418 postmenopausal women participating in UKCTOCS: a qualitative free-text analysis | 2019 | Menopause (New York, N.Y.) |
| Harper, J. and Broad, A. and Biswakarma, R. | A survey of women's experiences, behaviours and attitudes of using period tracker applications (apps) | 2022 | Human Reproduction |
| Harris, Sophie and Jenkinson, Elizabeth and Carlton, Edward and Roberts, Tom and Daniels, Jo | "It's Been Ugly": A Large-Scale Qualitative Study into the Difficulties Frontline Doctors Faced across Two Waves of the COVID-19 Pandemic | 2021 | International journal of environmental research and public health |
| Hatcher, A. M. and McBride, R. S. and Rebombo, D. and Munshi, S. and Khumalo, M. and Christofides, N. | Process evaluation of a community mobilization intervention for preventing men's partner violence use in peri-urban South Africa | 2020 | Evaluation and program planning |
| Haynes, E. and Green, J. and Garside, R. and Kelly, M. P. and Guell, C. | Gender and active travel: A qualitative data synthesis informed by machine learning | 2019 | International Journal of Behavioral Nutrition and Physical Activity |
| Hays, R. and Daker-White, G. | The care.data consensus? A qualitative analysis of opinions expressed on Twitter | 2015 | BMC public health |
| Heckert, Andrea and Forsythe, Laura P. and Carman, Kristin L. and Frank, Lori and Hemphill, Rachel and Elstad, Emily A. and Esmail, Laura and Lesch, Julie Kennedy | Researchers, patients, and other stakeholders' perspectives on challenges to and strategies for engagement | 2020 | Research involvement and engagement |
| Hefner, J. L. and Nembhard, I. M. and Brewster, A. L. | Aligning Health Care and Social Services for Patients with Complex Needs: The Multiple Roles of Interorganizational Relationships | 2021 | Advances in health care management |
| Helme, Donald W. and Noar, Seth M. and Allard, Suzanne and Zimmerman, Rick S. and Palmgreen, Philip and McClanahan, Karen J. | In-depth investigation of interpersonal discussions in response to a safer sex mass media campaign | 2011 | Health communication |
| Hirooka, K. and Fukahori, H. and Taku, K. and Izawa, S. and Ogawa, A. | Posttraumatic growth in bereaved family members of patients with cancer: a qualitative analysis | 2019 | Supportive Care in Cancer |
| Hitt, Rose and Zhuang, Jie and Anderson, Jennifer | Media Presentation of Breastfeeding Beliefs in Newspapers | 2018 | Health communication |
| Hobson, Deslyn T. G. and Meriwether, Kate V. and Francis, Sean L. and Kinman, Casey L. and Stewart, J. Ryan | Sentiment Analysis of Web Sites Related to Vaginal Mesh Use in Pelvic Reconstructive Surgery | 2019 | Female pelvic medicine & reconstructive surgery |
| Hogan, R. and Orr, F. and Fox, D. and Cummins, A. and Foureur, M. | Developing nursing and midwifery students' capacity for coping with bullying and aggression in clinical settings: Students' evaluation of a learning resource | 2018 | Nurse education in practice |
| Hokka, Minna and Melender, Hanna-Leena and Lehto, Juho T. and Kaakinen, Pirjo | Palliative Nursing Competencies Required for Different Levels of Palliative Care Provision: A Qualitative Analysis of Health Care Professionals' Perspectives | 2021 | Journal of palliative medicine |
| Hom, Melanie A. and Bauer, Brian W. and Stanley, Ian H. and Boffa, Joseph W. and Stage, Dese'Rae L. and Capron, Daniel W. and Schmidt, Norman B. and Joiner, Thomas E. | Suicide attempt survivors' recommendations for improving mental health treatment for attempt survivors | 2021 | Psychological services |
| Hou, Fengsu and Cerulli, J. D. Catherine and Wittink, M. B. E. Marsha N. and Caine, Eric D. and Thompson-Stone, M. A. Jennifer and Qiu, Peiyuan | Rural Chinese Women's Recognition of Intimate Partner Violence and their Potential Coping Strategies: A Qualitative Study | 2022 | Journal of Family Violence |
| Hoyt, Jenna and Hamon, Jessie K. and Krishnaratne, Shari and Houndekon, Emmanuel and Curry, Dora and Mbembe, Miriam and Marcus, Seth and Kambanje, Misozi and Pryor, Shannon and Barbra, Ariko Angela and Muhumuza, Herbert and Spilotros, Nathaly and Webster, Jayne | "It was my own decision": the transformational shift that influences a woman's decision to use contraceptives covertly | 2022 | BMC Women's Health |
| Hoyt, Jenna and Krishnaratne, Shari and Hamon, Jessie K. and Boudarene, Lydia and Chantler, Tracey and Demissie, Shiferaw Dechasa and Landegger, Justine and Moseti, Easterlina and Marcus, Seth and Kambanje, Misozi and Pryor, Shannon and Spilotros, Nathaly and Gnintoungbe, Marius and Curry, Dora and Webster, Jayne | "As a woman who watches how my family is... I take the difficult decisions": a qualitative study on integrated family planning and childhood immunisation services in five African countries | 2021 | Reproductive health |
| Htut, K. M. and Mon, M. M. and Aye, Z. M. and Ni, L. L. | Young key affected population in Myanmar: Are there any challenges in seeking information and care for HIV/sexually transmitted infections and reproductive health? | 2018 | F1000Research |
| Hughes, M. and Salamonson, Y. and Metcalfe, L. | Student engagement using multiple-attempt 'Weekly Participation Task' quizzes with undergraduate nursing students | 2020 | Nurse education in practice |
| Hwang, K. O. and Ottenbacher, A. J. and Green, A. P. and Cannon-Diehl, M. R. and Richardson, O. and Bernstam, E. V. and Thomas, E. J. | Social support in an Internet weight loss community | 2010 | International Journal of Medical Informatics |
| Jackson, L. A. and Dykeman, M. and Gahagan, J. and Karabanow, J. and Parker, J. | Challenges and opportunities to integrating family members of injection drug users into harm reduction efforts within the Atlantic Canadian context | 2011 | International Journal of Drug Policy |
| Jacobzon, Anna and EngstrÃ¶m, Ã…sa and Lindberg, Birgitta and Gustafsson, Silje Rysst | Mothers' strategies for creating positive breastfeeding experiences: a critical incident study from Northern Sweden | 2022 | International Breastfeeding Journal |
| Jalil, Aisha and Zakar, Rubeena and Zakar, Muhammad Zakria and Fischer, Florian | Patient satisfaction with doctor-patient interactions: a mixed methods study among diabetes mellitus patients in Pakistan | 2017 | BMC health services research |
| James, Arthur and Tran, Viet-Thi and Gauss, Tobias and Hamada, Sophie and Roquet, Florian and Bitot, Valerie and Boutonnet, Mathieu and Raux, Mathieu and Ravaud, Philippe | Important Issues to Severe Trauma Survivors: A Qualitative Study | 2022 | Annals of surgery |
| Jaramillo, Elise T. and Haozous, Emily and Willging, Cathleen E. | The Community as the Unit of Healing: Conceptualizing Social Determinants of Health and Well-Being for Older American Indian Adults | 2022 | The Gerontologist |
| Jenholt Nolbris, M. and Ragnarsson, S. and Brorsson, A. L. and Garcia de Avila, M. and Forsner, M. and Kull, I. and Olinder, A. L. and Mattson, J. and Nilsson, S. and Rullander, A. C. and Rydstrom, L. L. and Olaya-Contreras, P. and Berghammer, M. | Young children's voices in an unlocked Sweden during the COVID-19 pandemic | 2022 | Scandinavian journal of public health |
| Jones, Nev and Kosyluk, Kristin and Gius, Becky and Wolf, Jessica and Rosen, Cherise | Investigating the mobility of the peer specialist workforce in the United States: Findings from a national survey | 2020 | Psychiatric rehabilitation journal |
| Jones, N. and Pincock, K. and Emirie, G. and Gebeyehu, Y. and Yadete, W. | Supporting resilience among young people at risk of child abuse in Ethiopia: The role of social system alignment | 2021 | Child Abuse and Neglect |
| Jorm, C. and Roberts, C. and Lim, R. and Roper, J. and Skinner, C. and Robertson, J. and Gentilcore, S. and Osomanski, A. | A large-scale mass casualty simulation to develop the non-technical skills medical students require for collaborative teamwork | 2016 | BMC medical education |
| Jors, K. and Seibel, K. and Bardenheuer, H. and Buchheidt, D. and Mayer-Steinacker, R. and Viehrig, M. and Xander, C. and Becker, G. | Education in End-of-Life Care: What Do Experienced Professionals Find Important? | 2016 | Journal of cancer education : the official journal of the American Association for Cancer Education |
| Kalvesmaki, A. F. and Chapman, A. B. and Peterson, K. S. and Pugh, M. J. and Jones, M. and Gleason, T. C. | Analysis of a national response to a White House directive for ending veteran suicide | 2022 | Health Services Research |
| Karp, Melinda Mechur and Bork, Rachel Hare | "They never told me what to expect, so I didn't know what to do": Defining and clarifying the role of a community college student | 2014 | Teachers College Record |
| Kaye, E. C. and Applegarth, J. and Gattas, M. and Kiefer, A. and Reynolds, J. and Zalud, K. and Baker, J. N. | Hospice nurses request paediatric-specific educational resources and training programs to improve care for children and families in the community: Qualitative data analysis from a population-level survey | 2020 | Palliative Medicine |
| Kelly, Fiona and McMillan, Sara and Spinks, Jean and Bettington, Emilie and Wheeler, Amanda J. | You don't throw these things out:' an exploration of medicines retention and disposal practices in Australian homes | 2018 | BMC public health |
| Kemp, C. L. and Luo, S. and Ball, M. M. | "Meds are a real tricky area": Examining medication management and regulation in assisted living | 2012 | Journal of Applied Gerontology |
| Khanova, Julia and Roth, Mary T. and Rodgers, Jo Ellen and McLaughlin, Jacqueline E. | Student experiences across multiple flipped courses in a single curriculum | 2015 | Medical education |
| Kilcoyne, S. and Rogers, C. and Thomas, G. P. L. and Wall, S. and Johnson, D. | Craniofacial Surgery-Related Hashtag Utilisation on Instagram | 2021 | The Journal of craniofacial surgery |
| Kim, Junhee | The relationships among the learning transfer system, managers' creative learning transfer, and job performance | 2017 | Dissertation Abstracts International Section A: Humanities and Social Sciences |
| Kim, Wonsun and Kreps, Gary L. and Shin, Cha-Nam | The role of social support and social networks in health information-seeking behavior among Korean Americans: a qualitative study | 2015 | International journal for equity in health |
| Kisicki, A. and Becker, S. and Chaple, M. and Gustafson, D. H. and Hartzler, B. J. and Jacobson, N. and Murphy, A. A. and Tapscott, S. and Molfenter, T. | Behavioral healthcare organizations' experiences related to use of telehealth as a result of the COVID-19 pandemic: an exploratory study | 2022 | BMC health services research |
| Klaassen, H. and Dukes, K. and Marchini, L. | Patient satisfaction with dental treatment at a university dental clinic: A qualitative analysis | 2021 | Journal of dental education |
| Kolshus, E. M. and Akinmoluwa, S. and Sloan, D. and MacSuibhne, S. and Kolshus, E. H. | Patients' Attitudes Towards Medical Student Presence in Psychiatric Consultations | 2022 | Irish journal of psychological medicine |
| Kopecky, Kimberly E. and Zens, Tiffany J. and Suwanabol, Pasithorn A. and Schwarze, Margaret L. | Third-Year Medical Students' Reactions to Surgical Patients in Pain: Doubt, Distress, and Depersonalization | 2018 | Journal of pain and symptom management |
| Kostas-Polston, E. A. and Braun, L. A. and Miedema, J. | Female warfighters' perceptions of urogenital health in operational settings | 2020 | Journal of the American Association of Nurse Practitioners |
| Krzyzaniak, Natalia and Scott, Anna Mae and Bakhit, Mina and Bryant, A. N. N. and Taylor, Marianne and Mar, Chris Del | Impact of the COVID-19 pandemic on the Australian residential aged care facility (RACF) workforce | 2021 | Australian Journal of Advanced Nursing |
| Kwabla, M. P. and Klett-Tammen, C. J. and Castell, S. | Barriers and motivation for presumptive tuberculosis case referral: qualitative analysis among operators of community medicine outlets in Ghana | 2022 | BMC health services research |
| Laestadius, Linnea I. and Wahl, Megan M. and Pokhrel, Pallav and Cho, Young I. | From Apple to Werewolf: A content analysis of marketing for e-liquids on Instagram | 2019 | Addictive behaviors |
| Landers, Margaret and McCarthy, Geraldine and Savage, Eileen | Bowel symptom experiences and management following sphincter saving surgery for rectal cancer: A qualitative perspective | 2012 | European journal of oncology nursing : the official journal of European Oncology Nursing Society |
| Lane, J. V. and Hamilton, D. F. and MacDonald, D. J. and Ellis, C. and Howie, C. R. | Factors that shape the patient's hospital experience and satisfaction with lower limb arthroplasty: an exploratory thematic analysis | 2016 | BMJ open |
| Lane, Shannon R. and Kagotho, Njeri and McClendon, Jennifer and Flowers, Theresa D. and Vanidestine, Todd and Bogenschutz, Matthew | In their own words: Social work faculty discuss incivility | 2022 | Social Work Education |
| Latella, Lauren E. and Rogers, Madeline and Leventhal, Howard and Parker, Patricia A. and Horwitz, Steven and Matasar, Matthew J. and Bylund, Carma L. and Kissane, David W. and Franco, Kara and Banerjee, Smita C. | Fear of cancer recurrence in lymphoma survivors: A descriptive study | 2020 | Journal of Psychosocial Oncology |
| Lawson, J. L. and Doran, J. M. and O'Shea, M. K. and Abel, E. A. | The Good, The Bad, The Uncertain: Diverse Provider Experiences with Telemental Health During COVID-19 | 2022 | Psychiatric Quarterly |
| Leather, J. Z. and Keyworth, C. and Epton, T. and Goldthorpe, J. and Ulph, F. and Armitage, C. J. | "We want to live a little longer and our family want[s] us around": A summative content analysis of adherence to COVID-19-related guidelines using the Theoretical Domains Framework | 2022 | British journal of health psychology |
| Lee, Kai-Sean and Tao, Chen-Wei | Secretless pastry chefs on Instagram: The disclosure of culinary secrets on social media | 2021 | International Journal of Contemporary Hospitality Management |
| Lees, Shelley and Zalwango, Flavia and Andrew, Bahati and Vandepitte, Judith and Seeley, Janet and Hayes, Richard J. and Francis, Suzanna C. | Understanding motives for intravaginal practices amongst Tanzanian and Ugandan women at high risk of HIV infection: the embodiment of social and cultural norms and well-being | 2014 | Social science & medicine (1982) |
| Lehmann, Birthe A. and Ruiter, Robert A. C. and Kok, Gerjo | A qualitative study of the coverage of influenza vaccination on Dutch news sites and social media websites | 2013 | BMC public health |
| Lemke, S. and Vorster, H. H. and van Rensburg, N. S. Jansen and Ziche, J. | Empowered women, social networks and the contribution of qualitative research: broadening our understanding of underlying causes for food and nutrition insecurity | 2003 | Public health nutrition |
| Lemmon, Monica E. and Huffstetler, Hanna and Barks, Mary Carol and Kirby, Christine and Katz, Madelaine and Ubel, Peter A. and Docherty, Sharron L. and Brandon, Debra | Neurologic Outcome After Prematurity: Perspectives of Parents and Clinicians | 2019 | Pediatrics |
| Liebregts, N. and Rigoni, R. and Petruzelka, B. and Bartak, M. and Rowicka, M. and Zurhold, H. and Schiffer, K. | Different phases of ATS use call for different interventions: a large qualitative study in Europe | 2022 | Harm Reduction Journal |
| Limoges, Jacqueline and Jagos, Kim | The influences of nursing education on the socialization and professional working relationships of Canadian practical and degree nursing students: A critical analysis | 2015 | Nurse education today |
| Lindell, Deborah F. and Hagler, Debra and Poindexter, Kathleen | A National, Qualitative Study of the Motivators and Outcomes of Nurse Educator Certification | 2020 | Nursing education perspectives |
| Lingard, L. and Sue-Chue-Lam, C. and Tait, G. R. and Bates, J. and Shadd, J. and Schulz, V. and Heart Failure/Palliative Care Teamwork Research, Group | Pulling together and pulling apart: influences of convergence and divergence on distributed healthcare teams | 2017 | Advances in health sciences education : theory and practice |
| Linh, Trinh Thi and Giang, Ngo Thi Hoang and Khanh Ha, Truong Thi | When are children satisfied with life? An initiative qualitative study from vietnamese children sample | 2021 | Science progress |
| Litterbach, E. and Holmesâ€Truscott, E. and Pouwer, F. and Speight, J. and Hendrieckx, C. | I wish my health professionals understood that it's not just all about your HbA1c!'. Qualitative responses from the second Diabetes MILES â€“ Australia (MILESâ€2) study | 2020 | Diabetic Medicine |
| Littlewood, Donna L. and Quinlivan, Leah and Graney, Jane and Appleby, Louis and Turnbull, Pauline and Webb, Roger T. and Kapur, Navneet | Learning from clinicians' views of good quality practice in mental healthcare services in the context of suicide prevention: a qualitative study | 2019 | BMC psychiatry |
| Lohiniva, Anna-Leena and Pensola, Annika and Hyokki, Suvi and Sivela, Jonas and Tammi, Tuukka | COVID-19 risk perception framework of the public: an infodemic tool for future pandemics and epidemics | 2022 | BMC public health |
| Lopez, Andrea and Detz, Alissa and Ratanawongsa, Neda and Sarkar, Urmimala | What patients say about their doctors online: a qualitative content analysis | 2012 | Journal of general internal medicine |
| Lopez-Perez, Belen and Zuffiano, Antonio and Benito-Ambrona, Tamara | Cross-cultural differences in children's conceptualizations of happiness at school | 2021 | European Journal of Developmental Psychology |
| Lubi, Kadi and Simm, Kadri and Lempu, Kaja and Zameska, Jay and Eensalu-Lind, Angela | Other patients become a secondary priority:' perceptions of Estonian frontline healthcare professionals on the influence of COVID-19 on health (in)equality and ethical decision-making | 2022 | Journal of Communication in Healthcare |
| Luke, D. and Allen, P. and Arian, G. and Crawford, M. and Headen, S. and Spigner, C. and Tassler, P. and Ureda, J. | Teens' images of smoking and smokers | 2001 | Public Health Reports |
| Lundin, Karl and Mahdi, Soheil and Isaksson, Johan and BÃ¶lte, Sven | Functional gender differences in autism: An international, multidisciplinary expert survey using the International Classification of Functioning, Disability, and Health model | 2021 | Autism: The International Journal of Research & Practice |
| Lunt, J. and Hemming, S. and Burton, K. and Elander, J. and Baraniak, A. | What workers can tell us about post-COVID workability | 2022 | Occupational medicine (Oxford, England) |
| Luzius, Abbie and Dobbs, Page D. and Jozkowski, Kristen N. | College students' reasons for using different e-cigarette products: A mixed methods analysis | 2020 | Journal of American College Health |
| Mabuza, Langalibalele H. and Omole, Olufemi B. and Govender, Indiran and Ndimande, John V. | Reasons for inpatients not to seek clarity at Dr George Mukhari Academic Hospital, Pretoria | 2014 | African journal of primary health care & family medicine |
| Mackert, M. and Stanforth, D. and Garcia, A. A. | Undermining of Nutrition and Exercise Decisions: Experiencing Negative Social Influence | 2011 | Public Health Nursing |
| Maison, Dominika and Jaworska, Diana and Adamczyk, Dominika and Affeltowicz, Daria | The challenges arising from the COVID-19 pandemic and the way people deal with them. A qualitative longitudinal study | 2021 | PloS one |
| Manchaiah, Vinaya and Amlani, Amyn M. and Bricker, Christina M. and Whitfield, Clayton T. and Ratinaud, Pierre | Benefits and Shortcomings of Direct-to-Consumer Hearing Devices: Analysis of Large Secondary Data Generated From Amazon Customer Reviews | 2019 | Journal of Speech, Language & Hearing Research |
| Manning, Wendy D. and Smock, Pamela J. | Measuring and modeling cohabitation: New perspectives from qualitative data | 2005 | Special Issue: Theoretical and Methodological Issues in Studying Families. |
| Martey, Rosa Mikeal and Stromer-Galley, Jennifer and Consalvo, Mia and Wu, Jingsi and Banks, Jaime and Strzalkowski, Tomek | Communicating age in Second Life: The contributions of textual and visual factors | 2015 | New Media & Society |
| Martial, Charlotte and Cassol, Helena and Charland-Verville, Vanessa and Pallavicini, Carla and Sanz, Camila and Zamberlan, Federico and Vivot, Rocio Martinez and Erowid, Fire and Erowid, Earth and Laureys, Steven and Greyson, Bruce and Tagliazucchi, Enzo | Neurochemical models of near-death experiences: A large-scale study based on the semantic similarity of written reports | 2019 | Consciousness and Cognition: An International Journal |
| Matthews, T. and Fisher, H. L. and Bryan, B. T. and Danese, A. and Moffitt, T. E. and Qualter, P. and Verity, L. and Arseneault, L. | This is what loneliness looks like: A mixed-methods study of loneliness in adolescence and young adulthood | 2022 | International Journal of Behavioral Development |
| May, C. R. and Finch, T. L. and Cornford, J. and Exley, C. and Gately, C. and Kirk, S. and Jenkings, K. N. and Osbourne, J. and Robinson, A. L. and Rogers, A. and Wilson, R. and Mair, F. S. | Integrating telecare for chronic disease management in the community: what needs to be done? | 2011 | BMC health services research |
| Mazor, Kathleen M. and Gaglio, Bridget and Nekhlyudov, Larissa and Alexander, Gwen L. and Stark, Azadeh and Hornbrook, Mark C. and Walsh, Kathleen and Boggs, Jennifer and Lemay, Celeste A. and Firneno, Cassandra and Biggins, Colleen and Blosky, Mary Ann and Arora, Neeraj K. | Assessing Patient-Centered Communication in Cancer Care: Stakeholder Perspectives | 2013 | Journal of Oncology Practice |
| McClement, Susan E. and Fallis, Wendy M. and Pereira, Asha | Family presence during resuscitation: Canadian critical care nurses' perspectives | 2009 | Journal of nursing scholarship : an official publication of Sigma Theta Tau International Honor Society of Nursing |
| McCloskey, L. and Kennedy, H. P. and Declercq, E. R. and Williams, D. R. | The practice of nurse-midwifery in the era of managed care: reports from the field | 2002 | Maternal and child health journal |
| McDougall, Rosalind and Hayes, Barbara and Sellars, Marcus and Pratt, Bridget and Hutchinson, Anastasia and Tacey, Mark and Detering, Karen and Shadbolt, Cade and Ko, Danielle | This is uncharted water for all of us': challenges anticipated by hospital clinicians when voluntary assisted dying becomes legal in Victoria | 2020 | Australian Health Review |
| McHutchion, Lindsay D. and Pringle, Julia M. and Tran, My-Han N. and Ostevik, Amberley V. and Constantinescu, Gabriela | A survey of public awareness of dysphagia | 2021 | International Journal of Speech-Language Pathology |
| McKee, Patricia A. and Nelson, Toben F. and Toomey, Traci L. and Shimotsu, Scott T. and Hannan, Peter J. and Jones-Webb, Rhonda J. | Adopting local alcohol policies: a case study of community efforts to regulate malt liquor sales | 2012 | American journal of health promotion : AJHP |
| McKenzie, J. W. and Longman, J. M. and Bailie, R. and Braddon, M. and Morgan, G. G. and Jegasothy, E. and Bennett-Levy, J. | Insurance Issues as Secondary Stressors Following Flooding in Rural Australia-A Mixed Methods Study | 2022 | International Journal of Environmental Research and Public Health |
| McKinley, C. E. and Boel-Studt, S. and Renner, L. M. and Figley, C. R. and Billiot, S. and Theall, K. P. | The Historical Oppression Scale: Preliminary conceptualization and measurement of historical oppression among Indigenous peoples of the United States | 2020 | Transcultural Psychiatry |
| McQueen, A. and Kreuter, M. W. and Boyum, S. and Thompson, V. S. and Caburnay, C. A. and Waters, E. A. and Kaphingst, K. A. and Rath, S. and Fu, Q. | Reactions to FDA-proposed graphic warning labels affixed to U.S. smokers' cigarette packs | 2015 | Nicotine and Tobacco Research |
| Meeks, D. W. and Smith, M. W. and Taylor, L. and Sittig, D. F. and Scott, J. M. and Singh, H. | An analysis of electronic health record-related patient safety concerns | 2014 | Journal of the American Medical Informatics Association |
| Menon, S. and McCullough, L. B. and Beyth, R. J. and Ford, M. E. and Espadas, D. and Braun, U. K. | Use of a values inventory as a discussion aid about end-of-life care: A pilot randomized controlled trial | 2016 | Palliative & supportive care |
| Merolli, Mark and Busuttil, Maria-Louisa and WÃ¥hlin, Charlotte and Green, Ann | Global communication practices of physiotherapists on Twitter | 2019 | European Journal of Physiotherapy |
| Meylakhs, Peter and Rykov, Yuri and Koltsova, Olessia and Koltsov, Sergey | An AIDS-Denialist Online Community on a Russian Social Networking Service: Patterns of Interactions With Newcomers and Rhetorical Strategies of Persuasion | 2014 | Journal of Medical Internet Research |
| Michel, A. and Ryan, N. and Mattheus, D. and Knopf, A. and Abuelezam, N. N. and Stamp, K. and Branson, S. and Hekel, B. and Fontenot, H. B. | Undergraduate nursing students' perceptions on nursing education during the 2020 COVID-19 pandemic: A national sample | 2021 | Nursing outlook |
| Mieras, Adinda and Onwuteaka-Philipsen, Bregje D. and Becker-Commissaris, Annemarie and Bos, Jose C. M. and Pasman, H. Roeline W. | Relatives of deceased patients with metastatic lung cancer's views on the achievement of treatment goals and the choice to start treatment: a structured telephone interview study | 2020 | BMC palliative care |
| Miller, Jordan and Currie, Sinead and O'Carroll, Ronan E. | If I donate my organs it's a gift, if you take them it's theft': a qualitative study of planned donor decisions under opt-out legislation | 2019 | BMC public health |
| Mitchinson, Lucy and Dowrick, Anna and Buck, Caroline and Hoernke, Katarina and Martin, Sam and Vanderslott, Samantha and Robinson, Hannah and Rankl, Felicia and Manby, Louisa and Lewis-Jackson, Sasha and Vindrola-Padros, Cecilia | Missing the human connection: A rapid appraisal of healthcare workers' perceptions and experiences of providing palliative care during the COVID-19 pandemic | 2021 | Palliative Medicine |
| Mitev, Ariel | A narrative analysis of university students' alcohol stories in terms of a Fryeian framework | 2007 | European Journal of Mental Health |
| Mittal, Maria Luisa and Bazzi, Angela Robertson and Rangel, Maria Gudelia and Staines, Hugo and Yotebieng, Kelly and Strathdee, Steffanie A. and Syvertsen, Jennifer L. | He's not my pimp': toward an understanding of intimate male partner involvement in female sex work at the Mexico-US border | 2018 | Culture, health & sexuality |
| Moldestad, Megan and Stryczek, Krysttel C. and Haverhals, Leah and Kenney, Rachael and Lee, Marcie and Ball, Sherry and Au, David and Kirsh, Susan and Sayre, George and Young, Jessica | Competing demands: Scheduling challenges in being veteran-centric in the setting of health system initiatives to improve access | 2021 | Military Medicine |
| Monrouxe, L. V. and Bullock, A. and Gormley, G. and Kaufhold, K. and Kelly, N. and Roberts, C. E. and Mattick, K. and Rees, C. | New graduate doctors' preparedness for practice: A multistakeholder, multicentre narrative study | 2018 | BMJ Open |
| Moore, S. K. and Saunders, E. C. and McLeman, B. and Metcalf, S. A. and Walsh, O. and Bell, K. and Meier, A. and Marsch, L. A. | Implementation of a New Hampshire community-initiated response to the opioid crisis: A mixed-methods process evaluation of Safe Station | 2021 | International Journal of Drug Policy |
| Moreland, Jennifer J. and Apker, Julie | Conflict and Stress in Hospital Nursing: Improving Communicative Responses to Enduring Professional Challenges | 2016 | Health Communication |
| Moreland, Jennifer J. and Raup-Krieger, Janice L. and Hecht, Michael L. and Miller-Day, Michelle M. | The conceptualization and communication of risk among rural appalachian adolescents | 2013 | Journal of health communication |
| Morton, Kara F. and Pantalos, Diana C. and Ziegler, Craig and Patel, Pradip D. | A Place for Plant-Based Nutrition in US Medical School Curriculum: A Survey-Based Study | 2022 | American journal of lifestyle medicine |
| Mottram, Anne | Patients' experiences of day surgery: a Parsonian analysis | 2011 | Journal of advanced nursing |
| Moucheraud, Corrina and Stern, Amy F. and Ahearn, Canice and Ismail, Anisa and Nsubuga-Nyombi, Tamara and Ngonyani, Monica M. and Mvungi, Jane and Ssensamba, Jude | Barriers to HIV Treatment Adherence: A Qualitative Study of Discrepancies Between Perceptions of Patients and Health Providers in Tanzania and Uganda | 2019 | AIDS patient care and STDs |
| Murphy, Marie and Record, Helena and Callander, Jacquelyn K. and Dohan, Daniel and Grandis, Jennifer R. | Mentoring Relationships and Gender Inequities in Academic Medicine: Findings From a Multi-Institutional Qualitative Study | 2022 | Academic medicine : journal of the Association of American Medical Colleges |
| Nahm, Eun-Shim and Son, Hyojin and Yoon, Jung Min | Older adults' use of patient portals: Experiences, challenges, and suggestions shared through discussion board forums | 2020 | Geriatric nursing (New York, N.Y.) |
| Nallamothu, Brahmajee K. and Guetterman, Timothy C. and Harrod, Molly and Kellenberg, Joan E. and Lehrich, Jessica L. and Kronick, Steven L. and Krein, Sarah L. and Iwashyna, Theodore J. and Saint, Sanjay and Chan, Paul S. | How Do Resuscitation Teams at Top-Performing Hospitals for In-Hospital Cardiac Arrest Succeed? A Qualitative Study | 2018 | Circulation |
| Nayyar, D. and Pendrith, C. and Kishimoto, V. and Chu, C. and Fujioka, J. and Rios, P. and Sacha Bhatia, R. and Lyons, O. D. and Harvey, P. and O'Brien, T. and Martin, D. and Agarwal, P. and Mukerji, G. | Quality of virtual care for ambulatory care sensitive conditions: Patient and provider experiences | 2022 | International Journal of Medical Informatics |
| Neale, J. and Strang, J. | Naloxone--does over-antagonism matter? Evidence of iatrogenic harm after emergency treatment of heroin/opioid overdose | 2015 | Addiction (Abingdon, England) |
| Nelson, G. and Stefancic, A. and Rae, J. and Townley, G. and Tsemberis, S. and Macnaughton, E. and Aubry, T. and Distasio, J. and Hurtubise, R. and Patterson, M. and Stergiopoulos, V. and Piat, M. and Goering, P. | Early implementation evaluation of a multi-site housing first intervention for homeless people with mental illness: A mixed methods approach | 2014 | Evaluation and Program Planning |
| Nikolaou, Charoula Konstantia and Tay, Zoey and Leu, Jodie and Rebello, Salome Antonette and Te Morenga, Lisa and Van Dam, Rob M. and Lean, Michael Ernest John | Young People's Attitudes and Motivations Toward Social Media and Mobile Apps for Weight Control: Mixed Methods Study | 2019 | JMIR mHealth and uHealth |
| Nissanholtz-Gannot, Rachel and Shapiro, Ephraim | Community nurses and chronic disease in Israel: Professional dominance as a social justice issue | 2021 | Nursing inquiry |
| Nkhoma, Kennedy Bashan and Ebenso, Bassey and Akeju, David and Adejoh, Samuel and Bennett, Michael and Chirenje, Mike and Dandadzi, Adlight and Nabirye, Elizabeth and Namukwaya, Elizabeth and Namisango, Eve and Okunade, Kehinde and Salako, Omolola and Harding, Richard and Allsop, Matthew J. | Stakeholder perspectives and requirements to guide the development of digital technology for palliative cancer services: a multi-country, cross-sectional, qualitative study in Nigeria, Uganda and Zimbabwe | 2021 | BMC palliative care |
| Nolen, Erin and Siegel, Jaclyn A. and Pownall, Madeleine and Talbot, Catherine and Dann, Charlotte | "I feel more protective over my body:" A brief report on pregnant women's embodied experiences during the COVID-19 pandemic | 2022 | Body image |
| Nugus, Peter and Greenfield, David and Travaglia, Joanne and Braithwaite, Jeffrey | The politics of action research: "if you don't like the way things are going, get off the bus" | 2012 | Social science & medicine (1982) |
| Nuri, R. P. and Xu, X. and Aldersey, H. M. | Users' satisfaction and experiences in using assistive devices distributed by a rehabilitation centre in Bangladesh: a cross-sectional study | 2022 | Disability and rehabilitation. Assistive technology |
| O' Sullivan, Lydia and Feeney, Laura and Crowley, Rachel K. and Sukumar, Prasanth and McAuliffe, Eilish and Doran, Peter | An evaluation of the process of informed consent: views from research participants and staff | 2021 | Trials |
| O'Hara Tompkins, Nancy and Northrup, Karen and Grant, Josh and Weikle, Mary Folz and Long, Dustin and Bassler, John and Workman, Charlotte and Ramsey, Zachary and Jarrett, Traci and Sirk, Hannah and Cottrell, Lesley | Translating School Physical Education and Activity Policies into Practice: A Case Study | 2020 | Translational journal of the American College of Sports Medicine |
| O'Mahony, M. and Hegarty, J. and Rooney, V. M. | Making sense of turmoil: How women reconcile their emotional response to discovery of a potential breast cancer symptom | 2018 | Cancer Nursing |
| Odindo, M. A. and Mwanthi, M. A. | Role of governmental and non-governmental organizations in mitigation of stigma and discrimination among HIV/AIDS persons in Kibera, Kenya | 2008 | East African journal of public health |
| Ofei-Dodoo, Samuel and Goerl, Kyle and Moser, Scott | Exploring the Impact of Group Size on Medical Students' Perception of Learning and Professional Development During Clinical Rotations | 2018 | Kansas journal of medicine |
| Oketch, S. Y. and Kwena, Z. and Choi, Y. and Adewumi, K. and Moghadassi, M. and Bukusi, E. A. and Huchko, M. J. | Perspectives of women participating in a cervical cancer screening campaign with community-based HPV self-sampling in rural western Kenya: A qualitative study | 2019 | BMC Women's Health |
| Omilion-Hodges, Leah M. and Manning, Bryanna L. and Orbe, Mark P. | "Context matters:" An exploration of young adult social constructions of meaning about death and dying | 2019 | Health Communication |
| Ornelas, C. and Torres, J. M. and Torres, J. R. and Alter, H. and Taira, B. R. and Rodriguez, R. M. | Anti-immigrant rhetoric and the experiences of latino immigrants in the emergency department | 2021 | Western Journal of Emergency Medicine |
| Oser, T. K. and Oser, S. M. and Parascando, J. A. and Grisolano, L. A. and Krishna, K. B. and Hale, D. E. and Litchman, M. and Majidi, S. and Haidet, P. | Challenges and successes in raising a child with type 1 diabetes and autism spectrum disorder: Mixed methods study | 2020 | Journal of Medical Internet Research |
| Ouma, Lynette and Bozkurt, Burcu and Chanley, Jill and Power, Christine and Kakonge, Ronald and Adeyemi, Oluwatosin C. and Kudekallu, Ramya Jawahar and Leahy Madsen, Elizabeth | A cross-country qualitative study on contraceptive method mix: contraceptive decisionmaking among youth | 2021 | Reproductive health |
| Overby, M. S. and Mazeika, S. and DiFazio, M. and Ioli, J. and Birch, K. and Devorace, L. | Clinicians' Perspectives of Treatment for Lateralization Errors: A Quantitative and Qualitative Study | 2022 | Language, speech, and hearing services in schools |
| Paramo, Pablo | The significance of public places to the people of Bogota, and policy implications for the city as a learning environment (Colombia) | 2005 | Dissertation Abstracts International: Section B: The Sciences and Engineering |
| Peacey, Sarah J. | Opportunities for organization development: Hierarchy, subculture and participation in enterprise social media at IASL LLP | 2016 | Dissertation Abstracts International Section A: Humanities and Social Sciences |
| Peddie, Valerie L. and Whitelaw, Natalie and Cumming, Grant P. and Bhattacharya, Siladitya and Black, Mairead | Qualitative website analysis of information on birth after caesarean section | 2015 | BMC pregnancy and childbirth |
| Pedersen, Willy and Tutenges, SÃ©bastien and Sandberg, Sveinung | The pleasures of drunken one-night stands: Assemblage theory and narrative environments | 2017 | International Journal of Drug Policy |
| Peletz, R. and Kisiangani, J. and Bonham, M. and Ronoh, P. and Delaire, C. and Kumpel, E. and Marks, S. and Khush, R. | Why do water quality monitoring programs succeed or fail? A qualitative comparative analysis of regulated testing systems in sub-Saharan Africa | 2018 | International journal of hygiene and environmental health |
| Pene, Bobbieâ€Jo and Aspinall, Cathleen and Wilson, Denise and Parr, Jenny and Slark, Julia | Indigenous Maori experiences of fundamental care delivery in an acute inpatient setting: A qualitative analysis of feedback survey data | 2022 | Journal of Clinical Nursing (John Wiley & Sons, Inc.) |
| Pennay, Amy and Torronen, Jukka and Herold, Maria Dich and Fenton, Laura and MacLean, Sarah and Caluzzi, Gabriel and Fairbrother, Hannah and Frank, Vibeke A. and Samuelsson, Eva and Holmes, John | "There's a lot of stereotypes going on": A cross-national qualitative analysis of the place of gender in declining youth drinking | 2022 | The International journal on drug policy |
| Pentz, Rebecca D. and Pelletier, Wendy and Alderfer, Melissa A. and Stegenga, Kristin and Fairclough, Diane L. and Hinds, Pamela S. | Shared Decision-Making in Pediatric Allogeneic Blood and Marrow Transplantation: What If There Is No Decision to Make? | 2012 | Oncologist |
| Pereira, J. and Meadows, L. and Kljujic, D. and Strudsholm, T. and Parsons, H. and Riordan, B. and Faulkner, J. and Fisher, K. | Learner Experiences Matter in Interprofessional Palliative Care Education: A Mixed Methods Study | 2022 | Journal of Pain and Symptom Management |
| Perkins, R. and Mason-Bertrand, A. and Tymoszuk, U. and Spiro, N. and Gee, K. and Williamon, A. | Arts engagement supports social connectedness in adulthood: findings from the HEartS Survey | 2021 | BMC public health |
| Perry, Samuel L. | Social capital, race, and personal fundraising in evangelical outreach ministries | 2013 | Journal for the Scientific Study of Religion |
| Perz, J. and Ussher, J. and Gilbert, E. | Loss, uncertainty, or acceptance: subjective experience of changes to fertility after breast cancer | 2014 | European journal of cancer care |
| Pesut, Barbara and Duggleby, Wendy and Warner, Grace and Ghosh, Sunita and Bruce, Paxton and Dunlop, Rowena and Puurveen, Gloria | Scaling out a palliative compassionate community innovation: Nav-CARE | 2022 | Palliative care and social practice |
| Peterson, Colleen M. and Gaugler, Joseph E. | To speed or not to speed: Thematic analysis of American driving narratives | 2021 | Journal of Safety Research |
| Pfeiffer, D. and Holingue, C. and Dillon, E. and Kalb, L. and Reetzke, R. and Landa, R. | Parental concerns of children with ASD by age: A qualitative analysis | 2021 | Research in Autism Spectrum Disorders |
| Pfeiffer, Simone and In-Albon, Tina | Barriers to seeking psychotherapy for mental health problems in adolescents: A mixed method study | 2022 | Journal of Child and Family Studies |
| Philip, Keir E. J. and Lonergan, Bradley and Cumella, Andrew and Farrington-Douglas, Joe and Laffan, Michael and Hopkinson, Nicholas S. | COVID-19 related concerns of people with long-term respiratory conditions: a qualitative study | 2020 | BMC pulmonary medicine |
| Phillips, Georgina and Kendino, Mangu and Brolan, Claire E. and Mitchell, Rob and Herron, Lisa-Maree and Korver, Sarah and Sharma, Deepak and O'Reilly, Gerard and Poloniati, Penisimani and Kafoa, Berlin and Cox, Megan | Lessons from the frontline: Leadership and governance experiences in the COVID-19 pandemic response across the Pacific region | 2022 | The Lancet regional health. Western Pacific |
| Pitman, Alexandra and De Souza, Tanisha and Khrisna Putri, Adelia and Stevenson, Fiona and King, Michael and Osborn, David and Morant, Nicola | Support Needs and Experiences of People Bereaved by Suicide: Qualitative Findings from a Cross-Sectional British Study of Bereaved Young Adults | 2018 | International journal of environmental research and public health |
| Ploeg, Jenny and Matthew-Maich, Nancy and Fraser, Kimberly and Dufour, Sinead and McAiney, Carrie and Kaasalainen, Sharon and Markle-Reid, Maureen and Upshur, Ross and Cleghorn, Laura and Emili, Anna | Managing multiple chronic conditions in the community: a Canadian qualitative study of the experiences of older adults, family caregivers and healthcare providers | 2017 | BMC geriatrics |
| Pongiglione, B. and Kern, M. L. and Carpentieri, J. D. and Schwartz, H. A. and Gupta, N. and Goodman, A. | Do children's expectations about future physical activity predict their physical activity in adulthood? | 2020 | International Journal of Epidemiology |
| Ponnaiah, M. and Bhatnagar, T. and Ganeshkumar, P. and Bhar, D. and Elumalai, R. and Vijayageetha, M. and Abdulkader, R. S. and Chaudhuri, S. and Sharma, U. and Murhekar, M. V. | "Design and implementation challenges of massive open online course on research methods for Indian medical postgraduates and teachers -descriptive analysis of inaugural cycle" | 2022 | BMC medical education |
| Pool, R. and Montgomery, C. M. and Morar, N. S. and Mweemba, O. and Ssali, A. and Gafos, M. and Lees, S. and Stadler, J. and Crook, A. and Nunn, A. and Hayes, R. and McCormack, S. | A mixed methods and triangulation model for increasing the accuracy of adherence and sexual behaviour data: The microbicides development programme | 2010 | PLoS ONE |
| Poole, Grace and Pinto, Alex and Evans, Sharon and Ford, Suzanne and O'Driscoll, Mike and Buckley, Sharon and Ashmore, Catherine and Daly, Anne and MacDonald, Anita | Hungry for Change: The Experiences of People with PKU, and Their Caregivers, When Eating Out | 2022 | Nutrients |
| Postelnik, Talya and Robertson, Rhonda and Jury, Angela and Kongs-Taylor, Heather and Hetrick, Sarah and Tuason, Charito | Lived experience and clinical co-facilitation of a mental health literacy programme: qualitative exploration of satisfaction and factors supporting effective delivery | 2022 | Journal of Mental Health Training, Education & Practice |
| Pourette, Dolores and Pierlovisi, Carole and Randriantsara, Ranjatiana and Rakotomanana, Elliot and Mattern, Chiarella | Avoiding a "big" baby: Local perceptions and social responses toward childbirth-related complications in Menabe, Madagascar | 2018 | Social science & medicine (1982) |
| Powers, M. and Saberi, P. and Pepino, R. and Strupp, E. and Bugos, E. and Cannuscio, C. C. | Popular epidemiology and "fracking": citizens' concerns regarding the economic, environmental, health and social impacts of unconventional natural gas drilling operations | 2015 | Journal of community health |
| Prentice, Trisha M. and Gillam, Lynn and Davis, Peter G. and Janvier, Annie | Always a burden? Healthcare providers' perspectives on moral distress | 2018 | Archives of disease in childhood. Fetal and neonatal edition |
| Proctor, Gillian and Hargate, Rebecca | Quantitative and qualitative analysis of a set of goal attainment forms in primary care mental health services | 2013 | Counselling & Psychotherapy Research |
| Pulice-Farrow, Lex and McNary, Sebastian B. and Galupo, M. Paz | "Bigender is just a Tumblr thing": Microaggressions in the romantic relationships of gender non-conforming and agender transgender individuals | 2020 | Sexual and Relationship Therapy |
| Putney, Heather L. | Getting to the heart of the matter: Understanding relational satisfaction in modern-day couples | 2018 | Dissertation Abstracts International: Section B: The Sciences and Engineering |
| Quetsch, Lauren B. and Jackson, Carrie B. and Onovbiona, Harlee and Bradley, Rebecca | Caregiver decision-making on young child schooling/care in the face of COVID-19: The influence of child, caregiver, and systemic factors | 2022 | Children & Youth Services Review |
| Rahman, Abdul Rashid Abdul and Ji-Guang, Wang and Gary Mak Yiu, Kwong and Morales, Dante D. and Sritara, Piyamitr and Sukmawan, Renan | Perception of hypertension management by patients and doctors in Asia: potential to improve blood pressure control | 2015 | Asia Pacific Family Medicine |
| Raiman, Lewis and Antbring, Richard and Mahmood, Asad | WhatsApp messenger as a tool to supplement medical education for medical students on clinical attachment | 2017 | BMC medical education |
| Raj, Anita and Salazar, Marissa and Jackson, Emma C. and Wyss, Natalie and McClendon, Katherine A. and Khanna, Aarushi and Belayneh, Yemeserach and McDougal, Lotus | Students and brides: a qualitative analysis of the relationship between girls' education and early marriage in Ethiopia and India | 2019 | BMC public health |
| Randle, Reese W. and Bushman, Norah M. and Orne, Jason and Balentine, Courtney J. and Wendt, Elizabeth and Saucke, Megan and Pitt, Susan C. and Macdonald, Cameron L. and Connor, Nadine P. and Sippel, Rebecca S. | Papillary Thyroid Cancer: The Good and Bad of the "Good Cancer" | 2017 | Thyroid : official journal of the American Thyroid Association |
| Rapport, Frances L. and Jerzembek, Gabi S. and Doel, Marcus A. and Jones, Aled and Cella, Matteo and Lloyd, Keith R. | Narrating uncertainties about treatment of mental health conditions | 2010 | Social psychiatry and psychiatric epidemiology |
| Redshaw, M. and Henderson, J. | Learning the hard way: expectations and experiences of infant feeding support | 2012 | Birth (Berkeley, Calif.) |
| Redshaw, M. and Miller, Y. D. and Hennegan, J. | Young women's experiences as consumers of maternity care in Queensland | 2014 | Birth (Berkeley, Calif.) |
| Reed, Rachel and Sharman, Rachael and Inglis, Christian | Women's descriptions of childbirth trauma relating to care provider actions and interactions | 2017 | BMC pregnancy and childbirth |
| Reynolds, B. R. and Bulsara, C. and Zeps, N. and Codde, J. and Lawrentschuk, N. and Bolton, D. and Vivian, J. | Exploring pathways towards improving patient experience of robot-assisted radical prostatectomy (RARP): assessing patient satisfaction and attitudes | 2018 | BJU International |
| Ridosh, Monique M. and Sawin, Kathleen J. and Roux, Gayle and Brei, Timothy J. | Quality of Life in Adolescents and Young Adults with and Without Spina Bifida: An Exploratory Analysis | 2019 | Journal of pediatric nursing |
| Rink, K. A. and Turk, P. and Archibeque-Engle, S. L. and Wilmer, H. and Ahola, J. K. and Hadrich, J. C. and Roman-Muniz, I. N. | Dairy producer perceptions of the Farmers Assuring Responsible Management (FARM) Animal Care Program | 2019 | Journal of dairy science |
| Rissanen, Marjaâ€Liisa and KylmÃ¤, Jari and Hintikka, Jukka and Honkalampi, Kirsi and Tolmunen, Tommi and Laukkanen, Eila | Factors helping adolescents to stop self-cutting: descriptions of 347 adolescents aged 13-18 years | 2013 | Journal of Clinical Nursing (John Wiley & Sons, Inc.) |
| Robbins, Chloe J. and Allen, Harriet A. and Chapman, Peter | Comparing car drivers' and motorcyclists' opinions about junction crashes | 2018 | Accident; analysis and prevention |
| Robert and Delir Haghighi, Pari and Burstein, Frada and Urquhart, Donna and Cicuttini, Flavia | Investigating Individuals' Perceptions Regarding the Context Around the Low Back Pain Experience: Topic Modeling Analysis of Twitter Data | 2021 | Journal of medical Internet research |
| Robinson, I. and Stoyel, H. and Robinson, P. | If she had broken her leg, she would not have waited in agony 9 months: Caregiver's experiences of ED treatment and implications | 2021 | European Eating Disorders Review |
| Robinson, K. R. and Masud, T. and Hawley-Hague, H. | Instructors' Perceptions of Mostly Seated Exercise Classes: Exploring the Concept of Chair Based Exercise | 2016 | BioMed Research International |
| Rodgers, Rachel F. and Wertheim, Eleanor H. and Damiano, Stephanie R. and Gregg, Karen J. and Paxton, Susan J. | A qualitative, prospective study of children's understanding of weight gain | 2019 | The British journal of developmental psychology |
| Rodin, Diana and Silow-Carroll, Sharon and Cross-Barnet, Caitlin and Courtot, Brigette and Hill, Ian | Strategies to Promote Postpartum Visit Attendance Among Medicaid Participants | 2019 | Journal of women's health (2002) |
| Rodriguez, Maria Y. and Storer, Heather | A computational social science perspective on qualitative data exploration: Using topic models for the descriptive analysis of social media data* | 2020 | Journal of Technology in Human Services |
| Rolfe, A. and Dalton, S. and Krishnan, M. and Orford, J. and Mehdikhani, M. and Cawley, J. and Ferrins-Brown, M. | Alcohol, gender, aggression and violence: Findings from the Birmingham untreated heavy drinkers project | 2006 | Journal of Substance Use |
| Romero, D. and Kwan, A. and Suchman, L. | Methodologic approach to sampling and field-based data collection for a large-scale in-depth interview study: The Social Position and Family Formation (SPAFF) project | 2019 | PLoS ONE |
| Rosen, Anna and Ivarsson, Anneli and Nordyke, Katrina and Karlsson, Eva and Carlsson, Annelie and Danielsson, Lars and Hogberg, Lotta and Emmelin, Maria | Balancing health benefits and social sacrifices: a qualitative study of how screening-detected celiac disease impacts adolescents' quality of life | 2011 | BMC pediatrics |
| Rosen, Rebecca and Wieringa, Sietse and Greenhalgh, Trisha and Leone, Claudia and Rybczynska-Bunt, Sarah and Hughes, Gemma and Moore, Lucy and Shaw, Sara E. and Wherton, Joseph and Byng, Richard | Clinical risk in remote consultations in general practice: findings from in-COVID-19 pandemic qualitative research | 2022 | BJGP Open |
| Ross, Alyson and Bevans, Margaret and Friedmann, Erika and Williams, Laurie and Thomas, Sue | “I Am a Nice Person When I Do Yoga!!!”: A Qualitative Analysis of How Yoga Affects Relationships | 2014 | Journal of Holistic Nursing |
| Ross, Victoria and Kolves, Kairi and De Leo, Diego | Teachers' Perspectives on Preventing Suicide in Children and Adolescents in Schools: A Qualitative Study | 2017 | Archives of suicide research : official journal of the International Academy for Suicide Research |
| Rossolatos, George | The depth of brand engagement funnel: Dimensionalizing interaction in social media brand communities | 2021 | Qualitative Market Research: An International Journal |
| Roupetz, Sophie and Garbern, Stephanie and Michael, Saja and Bergquist, Harveen and Glaesmer, Heide and Bartels, Susan A. | Continuum of sexual and gender-based violence risks among Syrian refugee women and girls in Lebanon | 2020 | BMC women's health |
| Rowe, Theresa A. and Patel, Mallika and O'Conor, Rachel and McMackin, Sheila and Hoak, Vicki and Lindquist, Lee A. | COVID-19 exposures and infection control among home care agencies | 2020 | Archives of Gerontology & Geriatrics |
| Rozema, Emily J. and Creekmur, Beth and Musigdilok, Visanee V. and Steltz, Jennifer and Gould, Michael K. and Slatore, Christopher G. | Patient responses to passive enrollment into a large, pragmatic clinical trial: A qualitative content analysis | 2022 | Contemporary clinical trials |
| Russinova, Zlatka and Griffin, Shanta and Bloch, Philippe and Wewiorski, Nancy J. and Rosoklija, Ilina | Workplace prejudice and discrimination toward individuals with mental illnesses | 2011 | Journal of Vocational Rehabilitation |
| Rycroft-Malone, J. and Seers, K. and Eldh, A. C. and Cox, K. and Crichton, N. and Harvey, G. and Hawkes, C. and Kitson, A. and McCormack, B. and McMullan, C. and Mockford, C. and Niessen, T. and Slater, P. and Titchen, A. and van der Zijpp, T. and Wallin, L. | A realist process evaluation within the Facilitating Implementation of Research Evidence (FIRE) cluster randomised controlled international trial: an exemplar | 2018 | Implementation science : IS |
| Sacks, Emma and Mendez Alvarez, Montserrat and Bancalari, Pilar and Alegre, Juan-Carlos | Traditions and trust: a qualitative study of barriers to facility-based obstetric and immediate neonatal care in Chiapas, Mexico | 2022 | Women & health |
| Salasibew, M. M. and Moss, C. and Ayana, G. and Kuche, D. and Eshetu, S. and Dangour, A. D. | The fidelity and dose of message delivery on infant and young child feeding practice and nutrition sensitive agriculture in Ethiopia: a qualitative study from the Sustainable Undernutrition Reduction in Ethiopia (SURE) programme | 2019 | Journal of health, population, and nutrition |
| Samandari, Ghazaleh and Grant, Carolyn and Brent, Lily and Gullo, Sara | "It is a thing that depends on God": barriers to delaying first birth and pursuing alternative futures among newly married adolescent girls in Niger | 2019 | Reproductive health |
| Sanchez, P. R. P. and Folgado-Fernandez, J. A. and Sanchez, M. A. R. | Virtual Reality Technology: Analysis based on text and opinion mining | 2022 | Mathematical biosciences and engineering : MBE |
| Sandgren, Maria | Exploring personality and musical self-perceptions among vocalists and instrumentalists at music colleges | 2019 | Psychology of Music |
| Sanger, D. and Snow, P. C. and Colburn, C. and Gergen, M. and Ruf, M. | Speech-language pathologists' reactions to response to intervention: a qualitative study | 2012 | International journal of speech-language pathology |
| Santarossa, Sara and Coyne, Paige and Lisinski, Carly and Woodruff, Sarah J. | #fitspo on Instagram: A mixed-methods approach using Netlytic and photo analysis, uncovering the online discussion and author/image characteristics | 2019 | Journal of health psychology |
| Sarang, A. and Rhodes, T. and Platt, L. | Access to syringes in three Russian cities: implications for syringe distribution and coverage | 2008 | International Journal of Drug Policy |
| Sawyer, K. E. and Carpenter, A. T. and Coleman, R. D. and Tume, S. C. and Crawford, C. A. and Casas, J. A. | Provider Perceptions for Withdrawing Life Sustaining Therapies at a Large Pediatric Hospital | 2022 | Journal of Pain and Symptom Management |
| Schaffler, Yvonne and Gachter, Afsaneh and Dale, Rachel and Jesser, Andrea and Probst, Thomas and Pieh, Christoph | Concerns and Support after One Year of COVID-19 in Austria: A Qualitative Study Using Content Analysis with 1505 Participants | 2021 | International journal of environmental research and public health |
| Schiller, Shu Z. | CHAT for chat: Mediated learning in online chat virtual reference service | 2016 | Computers in Human Behavior |
| Schmidt, David and Reyment, Jill and Kirby, Sue and Webster, Emma L. and Lyle, David | The place of research in the rural health workplace structure: a content analysis of a rural health organisation's strategic and operational documents | 2020 | Rural and remote health |
| Schmitt, C. A. and Schiffman, R. | Perceived needs and coping resources of newly hired nurses | 2019 | SAGE Open Medicine |
| Scott, R. and Wallace, R. and Chary, S. | School pupils and understanding of significant change and losses in life | 2018 | Palliative Medicine |
| Scott, Samantha R. and Rivera, Kenia M. and Rushing, Ella and Manczak, Erika M. and Rozek, Christopher S. and Doom, Jenalee R. | "I Hate This": A Qualitative Analysis of Adolescents' Self-Reported Challenges During the COVID-19 Pandemic | 2021 | The Journal of adolescent health : official publication of the Society for Adolescent Medicine |
| Seear, K. H. and Spry, E. P. and Carlin, E. and Atkinson, D. N. and Marley, J. V. | Aboriginal women's experiences of strengths and challenges of antenatal care in the Kimberley: A qualitative study | 2021 | Women and Birth |
| Serafin, Alina and Franklin, Sarah and Mehta, Rashesh and Crosby, Scott and Lee, Diane and Edlin, Becky and Bewick, Bridgette M. | NHS patients, staff, and visitor viewpoints of smoking within a hospitals' ground: a qualitative analysis | 2014 | BMC public health |
| Shaffer, J. and Lieu, R. and Gutierrez, A. | Student perceived difficulties in learning organ systems in an undergraduate human anatomy course | 2017 | FASEB Journal |
| Shah-Beckley, Iduna and Clarke, Victoria and Thomas, Zoe | Therapists' and non-therapists' constructions of heterosex: A qualitative story completion study | 2020 | Psychology and psychotherapy |
| Shapiro, Johanna and Ortiz, Diane and Ree, You Ye and Sarwar, Minha | Medical students' creative projects on a third year pediatrics clerkship: a qualitative analysis of patient-centeredness and emotional connection | 2016 | BMC medical education |
| Shimkhada, Riti and Attai, Deanna and Scheitler, A. J. and Babey, Susan and Glenn, Beth and Ponce, Ninez | Using a Twitter Chat to Rapidly Identify Barriers and Policy Solutions for Metastatic Breast Cancer Care: Qualitative Study | 2021 | JMIR public health and surveillance |
| Shin, Jung-Hye | Living independently as an ethnic minority elder: a relational perspective on the issues of aging and ethnic minorities | 2014 | American journal of community psychology |
| Shipmon-Friedli, Shelia | An empirical investigation into the antecedents of the perceptions of work-life balance of professional women | 2022 | Dissertation Abstracts International: Section B: The Sciences and Engineering |
| Shiras, Tess and Cumming, Oliver and Brown, Joe and Muneme, Bacelar and Nala, Rassul and Dreibelbis, Robert | Shared latrines in Maputo, Mozambique: exploring emotional well-being and psychosocial stress | 2018 | BMC international health and human rights |
| Siegel, Karolynn and Meunier, Etienne and Lekas, Helen-Maria | Accounts for Unprotected Sex with Partners Met Online from Heterosexual Men and Women from Large US Metropolitan Areas | 2017 | AIDS patient care and STDs |
| Sikder, Shegufta S. and Labrique, Alain B. and Ullah, Barkat and Mehra, Sucheta and Rashid, Mahbubur and Ali, Hasmot and Jahan, Nusrat and Shamim, Abu A. and West, Keith P., Jr. and Christian, Parul | Care-seeking patterns for fatal non-communicable diseases among women of reproductive age in rural northwest Bangladesh | 2012 | BMC women's health |
| Simonovich, Shannon D. and Spurlark, Roxanne S. and Badowski, Donna and Krawczyk, Susan and Soco, Cheryl and Ponder, Tiffany N. and Rhyner, Debi and Waid, Rachel and Aquino, Elizabeth and Lattner, Christina and Wiesemann, Lucy Mueller and Webber-Ritchey, Kashica and Li, Suling and Tariman, Joseph D. | Examining effective communication in nursing practice during COVID-19: A large-scale qualitative study | 2021 | International nursing review |
| Simpson, Alan and Hannigan, Ben and Coffey, Michael and Jones, Aled and Barlow, Sally and Cohen, Rachel and Vseteckova, Jitka and Faulkner, Alison | Cross-national comparative mixed-methods case study of recovery-focused mental health care planning and co-ordination: Collaborative Care Planning Project (COCAPP) | 2016 |  |
| Singh, Samiksha and Upadhyaya, Sanjeev and Deshmukh, Pradeep and Dongre, Amol and Dwivedi, Neha and Dey, Deepak and Kumar, Vijay | Time motion study using mixed methods to assess service delivery by frontline health workers from South India: methods | 2018 | Human resources for health |
| Smith, Deborah Catherine | Secondary school staffrooms as perceived, conceived, and lived spaces: An investigation into their importance, decline, and sublation | 2015 | Dissertation Abstracts International Section A: Humanities and Social Sciences |
| Smith, Jacqueline and Smith, Jennifer and Mader, Joel and Guestier, Gabrielle and Conn, Lauren and Maddigan, Joy | An exploration of self-reported medicinal cannabis use among a sample of eastern canadian postsecondary students | 2021 | The Journal of Behavioral Health Services & Research |
| Smith, Sarah D. and Hall, Jean P. and Kurth, Noelle K. | Perspectives on health policy from people with disabilities | 2021 | Journal of Disability Policy Studies |
| Snir, Jessica T. and Ko, Danielle N. and Pratt, Bridget and McDougall, Rosalind | Anticipated impacts of voluntary assisted dying legislation on nursing practice | 2022 | Nursing ethics |
| Snook, A. G. and Arnadottir, S. A. and Forbes, R. | A survey of patient education practices and perceptions of physiotherapists: a mixed methods study | 2022 | Physiotherapy theory and practice |
| Snyder, Jeremy and Crooks, Valorie A. and Johnston, Rory and Ceron, Alejandro and Labonte, Ronald | "That's enough patients for everyone!": Local stakeholders' views on attracting patients into Barbados and Guatemala's emerging medical tourism sectors | 2016 | Globalization and health |
| Solheim, K. | Patterns of community relationship: nurses, non-governmental organizations and internally displaced persons | 2005 | International Nursing Review |
| Soltani, H. and Fair, F. J. and Watson, H. and Gardner, R. | Women's perspectives on antenatal breast expression: A cross-sectional survey | 2018 | Maternal and Child Nutrition |
| Sommers-Spijkerman, Marion and Elfrink, Teuntje R. and Drossaert, Constance H. C. and Schreurs, Karlein M. G. and Bohlmeijer, Ernst T. | Exploring compassionate attributes and skills among individuals participating in compassion-focused therapy for enhancing well-being | 2020 | Psychology and psychotherapy |
| Soprovich, A. L. and Wozniak, L. A. and Lee, C. and Sharma, V. and Samanani, S. and Eurich, D. T. | Appropriateness of COVID-19 public health guidelines for an Alberta First Nations community | 2022 | Canadian journal of public health = Revue canadienne de sante publique |
| Sousa, C. and Akesson, B. and Badawi, D. | Most importantly, I hope God keeps illness away from us': The context and challenges surrounding access to health care for Syrian refugees in Lebanon | 2020 | Global Public Health |
| South, Kelsey A. | College student survivors' evaluations of institutional responses to reports of sexual violence | 2018 | Dissertation Abstracts International: Section B: The Sciences and Engineering |
| Southwick, Frederick S. and Cranley, Nicole M. and Hallisy, Julia A. | A patient-initiated voluntary online survey of adverse medical events: the perspective of 696 injured patients and families | 2015 | BMJ quality & safety |
| Spitzer-Shohat, Sivan and Kay, Calanit and Hoshen, Moshe and Balicer, Ran D. and Shadmi, Efrat | Primary Care Networks and Team Effectiveness: The Case of a Large-Scale Quality Improvement Disparity Reduction Program | 2018 | International Journal of Integrated Care (IJIC) |
| Spoto, M. M. and Collins, J. | Physiotherapy diagnosis in clinical practice: a survey of orthopaedic certified specialists in the USA | 2008 | Physiotherapy research international : the journal for researchers and clinicians in physical therapy |
| Stanghellini, Giovanni and Ballerini, Massimo and Fernandez, Anthony Vincent and Cutting, John and Mancini, Milena | Abnormal Body Phenomena in Persons with Major Depressive Disorder | 2021 | Psychopathology |
| Stanghellini, Giovanni and Ballerini, Massimo and Presenza, Simona and Mancini, Milena and Northoff, Georg and Cutting, John | Abnormal Time Experiences in Major Depression: An Empirical Qualitative Study | 2017 | Psychopathology |
| Steiner-Hofbauer, V. and Grundnig, J. S. and Drexler, V. and Holzinger, A. | Now, I think doctors can be heroes ... Medical student's attitudes towards the COVID-19 pandemic's impact on key aspects of medical education and how the image of the medical profession has changed due to the COVID-19 pandemic | 2022 | Wiener Medizinische Wochenschrift |
| Stephens, Christine and Breheny, Mary | Diverse experiences among older adults in Aotearoa/New Zealand during COVID-19 lockdown: A qualitative study | 2022 | Australasian journal on ageing |
| Stern, Jenny and Molin, Moa Sterner and Fernaeus, Maja and Georgsson, Susanne and Carlsson, Tommy | Contraceptive counseling about adverse reactions of intrauterine contraception: Exploration of narratives found in web-based discussion boards | 2022 | Midwifery |
| Stevens, Matthew W. R. and Delfabbro, Paul H. and King, Daniel L. | Prevention approaches to problem gaming: A large-scale qualitative investigation | 2021 | Computers in Human Behavior |
| Stevens, Robin C. and Brawner, Bridgette M. and Kranzler, Elissa and Giorgi, Salvatore and Lazarus, Elizabeth and Abera, Maramawit and Huang, Sarah and Ungar, Lyle | Exploring Substance Use Tweets of Youth in the United States: Mixed Methods Study | 2020 | JMIR public health and surveillance |
| Stevenson, E. and Nicholson, H. and Smith-Han, K. | Medical students' experience of studying while working part-time and the effects of COVID-19 | 2022 | New Zealand Medical Journal |
| Stonelake-French, Heather and Moos, Brent E. and Brueggen, Carol M. and Gravemann, Erin L. and Hansen, Amy L. and Voll, Jeanne M. and Dose, Ann M. | Understanding Distress in the Hospital: A Qualitative Study Examining Adults With Cancer | 2018 | Oncology nursing forum |
| Strasser, Stephen and Bateman, Thomas S. | What we should study, problems we should solve: Perspectives of two constituencies | 1984 | Personnel Psychology |
| Strauss, Penelope and Winter, Sam and Waters, Zoe and Wright Toussaint, Dani and Watson, Vanessa and Lin, Ashleigh | Perspectives of trans and gender diverse young people accessing primary care and gender-affirming medical services: Findings from Trans Pathways | 2022 | International Journal of Transgender Health |
| Struik, L. and Yang, Y. | E-Cigarette cessation: Content analysis of a quit vaping community on reddit | 2021 | Journal of Medical Internet Research |
| Subramanian, Roma | Frames of Mental Illness in an Indian Daily Newspaper | 2019 | Health communication |
| Sullivan, Jennifer L. and Adjognon, Omonyele L. and Engle, Ryann L. and Shin, Marlena H. and Afable, Melissa K. and Rudin, Whitney and White, Bert and Shay, Kenneth and Lukas, Carol VanDeusen | Identifying and overcoming implementation challenges: Experience of 59 noninstitutional long-term services and support pilot programs in the Veterans Health Administration | 2018 | Health care management review |
| Sumankuuro, J. and Crockett, J. and Wang, S. | The use of antenatal care in two rural districts of Upper West Region, Ghana | 2017 | PLoS ONE |
| Sze, Yin and Dixon, Lesley and Paterson, Helen and Campbell, Norma | New Zealand LMC midwives' approaches to discussing nutrition, activity and weight gain during pregnancy | 2014 | New Zealand College of Midwives Journal |
| Tabbarah, Sarah | Exploratory mixed methods analysis of clinician habits, knowledge of, and attitudes towards evidence-based practices | 2020 | Dissertation Abstracts International: Section B: The Sciences and Engineering |
| Tapia, V. J. and Drizin, J. H. and Dalle Ore, C. and Nieto, M. and Romero, Y. and Magallon, S. and Nayak, R. and Sigler, A. and Malcarne, V. and Gosman, A. | Qualitative Methods in the Development of a Bilingual and Bicultural Quality of Life Outcomes Measure for Pediatric Patients With Craniofacial Conditions | 2017 | Annals of plastic surgery |
| Taylor, E. J. and Trippon, M. | What Chaplains Wish Nurses Knew: Findings From an Online Survey | 2020 | Holistic nursing practice |
| Taylor, Joanna and Pagliari, Claudia | #Deathbedlive: the end-of-life trajectory, reflected in a cancer patient's tweets | 2018 | BMC palliative care |
| Tebes, J. K. and Awad, M. N. and Connors, E. H. and Fineberg, S. K. and Gordon, D. M. and Jordan, A. and Kravitz, R. and Li, L. and Ponce, A. N. and Prabhu, M. and Rubman, S. and Silva, M. A. and Steinfeld, M. and Tate, D. C. and Xu, K. and Krystal, J. H. | The Stress and Resilience Town Hall: A systems response to support the health workforce during COVID-19 and beyond | 2022 | General Hospital Psychiatry |
| Thomas, Jeremy N. and Crosby, Lauren and Milford, Jessica | Gender differences among self-reported genital piercing stories | 2015 | Deviant Behavior |
| Timonen, Virpi and Conlon, Catherine and Scharf, Thomas and Carney, Gemma | Family, state, class and solidarity: Re-conceptualising intergenerational solidarity through the grounded theory approach | 2013 | European Journal of Ageing |
| Titeca, Kristof and Joossens, Luk and Raw, Martin | Blood cigarettes: cigarette smuggling and war economies in central and eastern Africa | 2011 | Tobacco Control |
| Tluczek, A. and Orland, K. M. and Nick, S. W. and Brown, R. L. | Newborn screening: an appeal for improved parent education | 2009 | Journal of Perinatal & Neonatal Nursing |
| Trankle, Steven A. and Usherwood, Tim and Abbott, Penelope and Roberts, Mary and Crampton, Michael and Girgis, Christian M. and Riskallah, John and Chang, Yashu and Saini, Jaspreet and Reath, Jennifer | Key stakeholder experiences of an integrated healthcare pilot in Australia: a thematic analysis | 2020 | BMC health services research |
| Treves-Kagan, Sarah and Naidoo, Evasen and Gilvydis, Jennifer M. and Raphela, Elsie and Barnhart, Scott and Lippman, Sheri A. | A situational analysis methodology to inform comprehensive HIV prevention and treatment programming, applied in rural South Africa | 2017 | Global public health |
| Tscholl, David W. and Weiss, Mona and Handschin, Lucas and Spahn, Donat R. and NÃ¶thiger, Christoph B. | User perceptions of avatar-based patient monitoring: a mixed qualitative and quantitative study | 2018 | BMC Anesthesiology |
| Tully, Kristin P. and Ball, Helen L. | Misrecognition of need: women's experiences of and explanations for undergoing cesarean delivery | 2013 | Social science & medicine (1982) |
| Tupesis, Janis P. and Lin, Janet and Nicks, Brett and Chiu, Arthur and Arbalaez, Christian and Wai, Abraham and Jouriles, Nic | Leadership Matters: Needs Assessment and Framework for the International Federation for Emergency Medicine Administrative Leadership Curriculum | 2021 | AEM education and training |
| Turale, Sue and Stone, Teresa Elizabeth and Warunee, Fongkaew | The Nutritional Health Beliefs of Nurses in Japan, Thailand, China and Australia | 2020 | Pacific Rim International Journal of Nursing Research |
| Underhill-Blazey, Meghan and Blonquist, Traci and Chittenden, Anu and Pozzar, Rachel and Nayak, Manan and Lansang, Kristina and Hong, Fangxin and Garber, Judy and Stopfer, Jill E. | Informing models of cancer genetic care in the era of multigene panel testing with patient-led recommendations | 2020 | Journal of Genetic Counseling |
| Ãœzar-Ã–zÃ§etin, Yeter Sinem and Russell-Westhead, Michele and Tee, Stephen | Workplace violence: A qualitative study drawing on the perspectives of UK nursing students | 2021 | Collegian |
| van de Beek, M. H. and Landman, E. and Veling, W. and Schoevers, R. A. and van der Krieke, L. | Discussing the unspoken: A qualitative analysis of online forum discussions on mental health problems in young Moroccan-Dutch migrants | 2022 | Transcultural Psychiatry |
| van den Ende, Eva S. and Schouten, Bo and Kremers, Marjolein N. T. and Cooksley, Tim and Subbe, Chris P. and Weichert, Immo and van Galen, Louise S. and Haak, Harm R. and Kellett, John and Alsma, Jelmer and Siegrist, Victoria and Holland, Mark and Christensen, Erika F. and Graham, Colin A. and Leung, Ling Yan and Laugesen, Line E. and Merten, Hanneke and Mir, Fraz and Kidney, Rachel M. and Brabrand, Mikkel and Nanayakkara, Prabath W. B. and Nickel, Christian H. and all local, collaborators | Understanding what matters most to patients in acute care in seven countries, using the flash mob study design | 2021 | BMC health services research |
| van Diggele, C. and Roberts, C. and Haq, I. | Optimising student-led interprofessional learning across eleven health disciplines | 2021 | BMC medical education |
| Van Erkel, F. M. and Pet, M. J. and Bossink, E. H. M. and Van De Graaf, C. F. M. and Hodes, M. T. J. and Van Ogtrop, S. N. and Mourits, M. J. E. and Welker, G. A. and Halmos, G. B. and Van Leeuwen, B. and Racz, E. and Reyners, A. K. L. and Van Munster, B. C. and Van Der Zaag-Loonen, H. J. | Experiences of patients and health care professionals on the quality of telephone follow-up care during the COVID-19 pandemic: A large qualitative study in a multidisciplinary academic setting | 2022 | BMJ Open |
| Van Stee, Stephanie K. and Noar, Seth M. and Allard, Suzanne and Zimmerman, Rick and Palmgreen, Philip and McClanahan, Kitty | Reactions to safer-sex public service announcement message features: attention, perceptions of realism, and cognitive responses | 2012 | Qualitative health research |
| Vasilescu, C. and Garel, M. and Caeymaex, L. | [Experience of parents after the loss of a newborn twin in the NICU: a qualitative study 3 years after the death] | 2013 | Vecu de parents ayant perdu un jumeau en reanimation neonatale : etude qualitative, 3 ans apres le deces. |
| Vasileva, Mira and Alisic, Eva and De Young, Alex | COVID-19 unmasked: Preschool children's negative thoughts and worries during the COVID-19 pandemic in Australia | 2021 | European Journal of Psychotraumatology |
| Verhoeven, Veronique and Tsakitzidis, Giannoula and Philips, Hilde and Van Royen, Paul | Impact of the COVID-19 pandemic on the core functions of primary care: will the cure be worse than the disease? A qualitative interview study in Flemish GPs | 2020 | BMJ open |
| Vermeulen, Joeri and Bilsen, Johan and Buyl, Ronald and De Smedt, Delphine and Gucciardo, Leonardo and Faron, Gilles and Fobelets, Maaike | Women's experiences with being pregnant and becoming a new mother during the COVID-19 pandemic | 2022 | Sexual & reproductive healthcare : official journal of the Swedish Association of Midwives |
| Viney, Rowena and Rich, Antonia and Needleman, Sarah and Griffin, Ann and Woolf, Katherine | The validity of the Annual Review of Competence Progression: a qualitative interview study of the perceptions of junior doctors and their trainers | 2017 | Journal of the Royal Society of Medicine |
| Virk, Punit and Atwal, Amanbir and Wright, Bruce and Doan, Quynh | Exploring parental perceptions of psychosocial screening in paediatric emergency departments | 2022 | Clinical Child Psychology & Psychiatry |
| Vlckova, Karolina and Polakova, Kristyna and Tuckova, Anna and Houska, Adam and Loucka, Martin | Views of patients with advanced disease and their relatives on participation in palliative care research | 2021 | BMC palliative care |
| Volioti, Georgia and Williamon, Aaron | Performers' discourses on listening to recordings | 2021 | Research Studies in Music Education |
| Waling, Andrea and Bellamy, Roz and Ezer, Paulina and Kerr, Lucille and Lucke, Jayne and Fisher, Christopher | It's kinda bad, honestly': Australian students' experiences of relationships and sexuality education | 2020 | Health Education Research |
| Walters, Cyrill and Ronnie, Linda and Jansen, Jonathan and Kriger, Samantha | "Academic guilt": The impact of the pandemic-enforced lockdown on women's academic work | 2021 | Women's Studies International Forum |
| Ware, N. C. and Wyatt, M. A. and Tugenberg, T. | Social relationships, stigma and adherence to antiretroviral therapy for HIV/AIDS | 2006 | AIDS Care |
| Warner, Robert P. and Sibthorp, Jim and Wilson, Cait and Browne, Laurie P. and Barnett, Sabrica and Gillard, Ann and Sorenson, Jacob | Similarities and differences in summer camps: A mixed methods study of lasting outcomes and program elements | 2021 | Children and Youth Services Review |
| Waters, Erika A. and McQueen, Amy and Caburnay, Charlene A. and Boyum, Sonia and Sanders Thompson, Vetta L. and Kaphingst, Kimberly A. and Kreuter, Matthew W. | Perceptions of the US National Tobacco Quitline Among Adolescents and Adults: A Qualitative Study, 2012-2013 | 2015 | Preventing chronic disease |
| Watts, Emily J. and Fackrell, Kathryn and Smith, Sandra and Sheldrake, Jacqueline and Haider, Haula and Hoare, Derek J. | Why Is Tinnitus a Problem? A Qualitative Analysis of Problems Reported by Tinnitus Patients | 2018 | Trends in hearing |
| Weaver, R. and Salamonson, Y. and Koch, J. and Jackson, D. | Nursing on television: Student perceptions of television's role in public image, recruitment and education | 2013 | Journal of Advanced Nursing |
| Wellecke, C. and D'Cruz, K. and Winkler, D. and Douglas, J. and Goodwin, I. and Davis, E. and Mulherin, P. | Accessible design features and home modifications to improve physical housing accessibility: A mixed-methods survey of occupational therapists | 2022 | Disability and Health Journal |
| Westberg, Kate and Stavros, Constantino and Smith, Aaron C. T. and Munro, Geoff and Argus, Kevin | An examination of how alcohol brands use sport to engage consumers on social media | 2018 | Drug & Alcohol Review |
| Wilk, Violetta and Soutar, Geoffrey N. and Harrigan, Paul | Tackling social media data analysis: Comparing and contrasting QSR NVivo and Leximancer | 2019 | Qualitative Market Research: An International Journal |
| Williams, Pamela Holtzclaw and Nemeth, Lynne S. and Sanner, Jennifer E. and Frazier, Lorraine Q. | Thematic analysis of cardiac care patients' explanations for declining contribution to a genomic research-based biobank | 2013 | American journal of critical care : an official publication, American Association of Critical-Care Nurses |
| Willis, L. D. and Lovenstein, A. and Spray, B. J. and Honeycutt, M. and Walden, M. | Practices and Perceptions of Face Mask Use in a Pediatric Health System During the COVID-19 Pandemic | 2021 | Respiratory Care |
| Winters Fisher, Allison F. | Embodied warrior wellness: Dance/movement therapy-based mind-body medicine in an integrative medicine treatment program for military members with traumatic brain injury | 2020 | Dissertation Abstracts International Section A: Humanities and Social Sciences |
| Wittich, C. M. and Mauck, K. F. and Mandrekar, J. N. and Gluth, K. A. and West, C. P. and Litin, S. C. and Beckman, T. J. | Improving participant feedback to continuing medical education presenters in internal medicine: A mixed-methods study | 2012 | Journal of General Internal Medicine |
| Wong, Susan P. Y. and Foglia, Mary Beth and Cohen, Jennifer and Oestreich, Taryn and O'Hare, Ann M. | The VA Life-Sustaining Treatment Decisions Initiative: A qualitative analysis of veterans with advanced kidney disease | 2022 | Journal of the American Geriatrics Society |
| Woolham, John and Norrie, Caroline and Samsi, Kritika and Manthorpe, Jill | The employment conditions of social care personal assistants in England | 2019 | Journal of Adult Protection |
| Wright, Erin M. and Matthai, Maude Theo and Budhathoki, Chakra | Midwifery Professional Stress and Its Sources: A Mixed-Methods Study | 2018 | Journal of midwifery & women's health |
| Wu, E. X. and Collins, A. and Briggs, S. and Stajduhar, K. I. and Kalsi, A. and Hilliard, N. | Prolonged Grief and Bereavement Supports Within a Caregiver Population Who Transition Through a Palliative Care Program in British Columbia, Canada | 2022 | The American journal of hospice & palliative care |
| Wurz, A. and Culos-Reed, S. N. and Franklin, K. and DeMars, J. and Wrightson, J. G. and Twomey, R. | "I feel like my body is broken": exploring the experiences of people living with long COVID | 2022 | Quality of Life Research |
| Xie, Hongling | The development and functions of social aggression: A narrative analysis of social exchange in interpersonal conflicts | 2000 | Dissertation Abstracts International: Section B: The Sciences and Engineering |
| Xing, Wanli and Popov, Vitaliy and Zhu, Gaoxia and Horwitz, Paul and McIntyre, Cynthia | The effects of transformative and non-transformative discourse on individual performance in collaborative-inquiry learning | 2019 | Computers in Human Behavior |
| Yager, Zali and Prichard, Ivanka and Hart, Laura M. | #Ihaveembraced: a pilot cross-sectional naturalistic evaluation of the documentary film Embrace and its potential associations with body image in adult women | 2020 | BMC women's health |
| Yanchus, Nancy J. and Carameli, Kelley A. and Ramsel, Dee and Osatuke, Katerine | How to make a job more than just a paycheck: Understanding physician disengagement | 2020 | Health care management review |
| Yaphe, Hannah and Adekoya, Itunuoluwa and Steiner, Liane and Maraj, Darshanand and O'Campo, Patricia and Persaud, Nav | Exploring the experiences of people in Ontario, Canada who have trouble affording medicines: a qualitative concept mapping study | 2019 | BMJ open |
| Yasin, S. A. | Prevalence, intensity and manifestation of covid-19 fear: A cross sectional analysis | 2021 | Psychiatria Danubina |
| Yazdani, A. T. and Muhammad, A. and Nisar, M. I. and Khan, U. and Shafiq, Y. | Unveiling and addressing implementation barriers to routine immunization in the peri-urban slums of Karachi, Pakistan: a mixed-methods study | 2021 | Health Research Policy and Systems |
| Yeoman, A. J. and Cooper, J. M. and Urquhart, C. J. and Tyler, A. | The management of health library outreach services: evaluation and reflection on lessons learned on the VIVOS project | 2003 | Journal of the Medical Library Association |
| Yu, Mei-Kuei and Liao, Min-Yu | Taiwanese protective social workers meeting elected representatives: Perspectives of protective social workers | 2020 | Journal of Social Service Research |
| Zamboni, B. D. | A Qualitative Exploration of Adult Baby/Diaper Lover Behavior From an Online Community Sample | 2019 | Journal of sex research |
| Zebroff, P. | Questionnaire for Turn-on Initiation Preference: Development and Initial Reliability and Validation | 2021 | Journal of sex research |
| Zhao, H. and Souders, C. P. and Freedman, A. and Breyer, B. and Anger, J. T. | The applicant's perspective on urology residency interviews: A qualitative analysis | 2020 | Journal of Urology |
| Zickmund, S. L. and Yang, S. and Mulvey, E. P. and Bost, J. E. and Shinkunas, L. A. and Labrecque, D. R. | Predicting cancer mortality: Developing a new cancer care variable using mixed methods and the quasi-statistical approach | 2013 | Health Services Research |
| DeJonckheere, Melissa; Zhao, Aisling; Lane, Jenni; Plegue, Melissa A.; Ranalli, Lauren; Wagner, Ellen; Riley, Margaret; | Results of a national text message poll of youth: Perspectives on primary care | 2020 | Journal of Adolescent Health |
| Knudsen, Gry Hongsmark; Kjeldgaard, Dannie | Online reception analysis: Big data in qualitative marketing research | 2014 | In Consumer culture theory |
| Petroff, Alisa; Viladrich, Anahí; Parella, Sònia; | Framing inclusion: The media treatment of irregular immigrants' right to health care in Spain | 2021 | International Journal of Intercultural Relations |
| White DE, Oelke ND, Friesen S. | Management of a Large Qualitative Data Set: Establishing Trustworthiness of the Data | 2012 | International journal of qualitative methods |
| Brinton MC, Bueno X, Oláh L, Hellum M. | Postindustrial Fertility Ideals, Intentions, and Gender Inequality: A Comparative Qualitative Analysis | 2018 | Population and Development Review |
| Towler L, Bondaronek P, Papakonstantinou T, Amlôt R, Chadborn T, Ainsworth B, Yardley L. | Applying machine-learning to rapidly analyse large qualitative text datasets to inform the COVID-19 pandemic response: Comparing human and machine-assisted topic analysis techniques | 2022 | medRxiv |
| Edwards R, Weller S, Davidson E, Jamieson L | Small Stories of Home Moves: A Gendered and Generational Breadth-and-Depth Investigation | 2023 | Sociological Research Online |
| Neufeld LM, Andrade EB, Suleiman AB, Barker M, Beal T, Blum LS, Demmler KM, Dogra S, Hardy-Johnson P, Lahiri A, Larson N. | Food choice in transition: adolescent autonomy, agency, and the food environment | 2022 | The Lancet |
| Setiawan C. | Representing Physical Education in Social Media: A Summative Content Analysis of A School Subject Through Big Data Analytics | 2023 | European Journal of Educational Research |
| Szymkowiak M., Steinkruger A. | Alaska fishers attest to climate change impacts in discourse on resource management under marine heatwaves | 2023 | Environmental Science and Policy |
| Bernardi C.L., Alhamdan N. | Social media analytics for nonprofit marketing: #Downsyndrome on Twitter and Instagram | 2022 | Journal of Philanthropy and Marketing |
| Cain P., Alan J., Porock D. | Emergency department transfers from residential aged care: what can we learn from secondary qualitative analysis of Australian Royal Commission data? | 2022 | BMJ Open |
| Park H., Biddix J.P., Park H.W. | Discussion, news information, and research sharing on social media at the onset of covid-19 | 2021 | Profesional de la Informacion |
| Choi J.-A., Park S. | Infodemiological study on the use of face masks during covid-19: Comparing U.S. and Korea [Infodemiološka studija o upotrebi maski za lice tijekom pandemije COVID-19: Usporedba sad-a i južne koreje. | 2021 | Drustvena Istrazivanja |
| Purcell C., Maxwell K., Bloomer F., Rowlands S., Hoggart L. | Toward normalising abortion: findings from a qualitative secondary analysis study | 2020 | Culture, Health and Sexuality |

## Appendix 6. List of identified data sources.

- Open-ended survey
- Interviews
- First-person narratives
- Focus groups
- Observations
- Tweets
- Social media
- Documents
- Forum messages
- Internet posts
- Public comments
- Medical records
- Audio-recorded conversations
- Notes
- Newspaper articles
- Videos
- Written responses
- Emails
- Chatbox messages
- Images
- Meeting transcripts
- Text messages
- Written reports
- YouTube comments and user details
- Dissertation abstracts
- Emojis
- Instagram images
- Instagram post hashtags
- Letters
- Narrative comments
- Policies
- Presentations

## Appendix 7. All methods used to analyse large bodies of qualitative data

- thematic analysis
- content analysis
- grounded theory
- consensual qualitative research
- framework analysis
- descriptive analysis
- horizontal thematic approach
- sentiment analysis
- visual mapping of themes
- Latent Dirichlet Association
- exploratory analysis
- cluster analysis
- matrix analysis
- discourse analysis
- narrative inquiry
- breadth and depth method
- concept mapping
- constructionist theoretical approach
- manifest content analysis Graneheim and Lundman 2004
- semantic coding
- social network analysis
- adhering to frameworks/theories
- machine learning approach
- assessment of bias
- content analysis
- convergence coding matrix
- Flanagan's Critical Incident Technique
- granular analysis
- Immersion-crystallization approach
- merged concurrent nested approach
- network analysis
- principles of digital ethnography
- Qualitative comparative analysis
- Qualitative inquiry approach
- semantic network analysis
- semantic themes
- thematic framework approach Gale 2013
- topic modelling

## Appendix 8. All steps used to analyse big qualitative datasets.

- multiple coders
- team discussion
- theme identification
- inductive coding
- categorising codes
- codebook
- coding
- cross-checking
- data familiarisation
- iterative approach
- frequency quantification of findings
- intercoder agreement
- deductive coding
- constant comparison
- open coding
- a priori themes
- triangulation
- validation of findings
- collaborative coding
- memoing
- reflexivity
- adhering to frameworks/theories
- comparison with literature
- staged approach
- iterative coding
- quotes to illustrate themes
- audit trail
- in-depth analysis of sub-sample
- manual coding
- data saturation
- translation
- interpretive approach
- transcription
- axial coding
- data reduction
- multidisciplinary diverse team
- blinded coders
- fragment selection
- co-researching with lived experience, community, and local researchers
- comparative case studies
- concurrent data collection and analysis
- double coding
- semantic coding
- stakeholder engagement
- subgroup analysis
- selective coding
- recursive abstraction
- peer review of analysis
- member checking
- text mining
- deviant cases
- cluster analysis
- constructing a corpus
- nodes/tree nodes
- realist approach
- thematic analysis
- visual mapping of themes
- cohen's kappa coefficient
- assessment of bias
- concept mapping
- consensual qualitative research
- content analysis
- descriptive analysis
- exploratory analysis
- grouping analysis by participants
- machine learning approach
- narrative inquiry
- natural language processing
- Netnography
- pre-processing data
- template analysis
- anonymisation of data
- flow chart development based on themes
- abduction
- analytic narratives] multiple coders
- betweenness centrality
- computational techniques
- convergence coding matrix
- discourse analysis
- economy of effort approach
- human analysis
- in vivo coding
- integrative analysis
- keyword searching
- machine learning image analysis
- Machine-Assisted Topic Analysis
- manifest content analysis
- network analysis
- path analysis
- piloting coding
- principles of digital ethnography
- quality appraisal of data source
- quantification using machine learning
- semantic network analysis
- semantic themes
- sentiment analysis
- social network analysis
- summary tabulation
- supervised researchers
- team discusson
- topic modelling
- trained researchers
- training machine learning
- triple coding
- unfolding approach
- unsupervised topic modelling
- word lemmatization
- SWOT analysis
- assessment of power dynamics

## Appendix 9. All citations referenced in review sample.

- Braun and Clarke
- Hsieh and Shannon 2005
- Corbin and Strauss 2008
- Glaser and Strauss 1967
- Elo and Kyngas 2008
- Ragin 1994
- Sandelowski 2000
- Charmaz 2006
- Miles and Huberman 1994
- manifest content analysis Graneheim and Lundman 2004
- Morse 2008
- Krippendorf's alpha 2004
- Lincoln and Guba 1985
- Saldana 2016
- Guest et al. 2012
- Landis and Koch 1977
- Elo et al. 2014
- Fereday and Muir-Cochrane 2006
- Lofland et al. 2005
- Mayring 2017
- Ritchie and Spencer 1994
- Schreier 2012
- Bengtsson 2016
- Miles et al. 2014
- Neuendorf 2002
- Patton 2001
- Ritchie et al. 2013
- thematic framework approach Gale 2013
- Thomas 2006
- Glaser 1992
- Spangler et al. 2012
- Blei et al. 2003
- Macqueen et al. 1998
- Hill et al. 2005
- Graneheim et al. 2017
- Jackson and Trochim 2002
- amplified analysis Heaton 2004
- Atkinson and Doody 2017
- Beebe 2014
- behaviour change wheel Michie et al. 2011
- Boyatzis 1998
- Bradshaw
- Burnard 1991
- Castro et al. 2010
- Charmaz and Belgrave 2012
- Crabtree and Miller 1999
- Cresswell 2007
- Creswell 2013
- Creswell and Plano Clark 2011
- Graneheim and Lundman 2004
- Kane and Trochim 2007
- Kuckartz 2010
- Lindloff and Taylor 2011
- Marks 2015
- Mayring and Fenzl 2019
- McMahon 2018
- Milne and Oberle 2005
- Morrow 2005
- Morse and Richards 2002
- Moustakas 1994
- Pope and Mays 2006
- quasi-statistical qualitative approach Miller and Crabtree 1992
- Roberts et al. 2016
- Saunders et al. 2018
- Sidnell and Stivers 2013
- Smith 2000
- Spyer and Steedly 2013
- Tashakkori and Creswell 2007
- Teddlie and Tashakkori 2009
- Thornberg 2012
- Ulin et al.
- Vaismoradi et al. 2013
- Desantis and Ugarizza 2000
- Bliss et al. 1983
- Patton 2015
- Glesne 2010
- Fayyad et al. 1996
- Heidegger 1953
- Strauss and Corbin 1998
- Koch 1994
- Emerson et al. 1995
- Carter et al. 2014
- Ryan and Bernard 2003
- Saldana 2013
- Lofland and Lofland 1995
- Bradley et al. 2007
- Gioia et al. 2012
- Lazard and Macavoy 2020
- Lofland and Lofland 1983

## Appendix 10. All software used to analyse large bodies of qualitative data

- NVivo
- Microsoft Excel
- Atlas.ti
- MaxQDA
- Microsoft Word
- R
- Dedoose
- SPSS
- NUD*IST
- Leximancer
- Machine learning
- NodeXL
- QDA Miner
- Treato-data mining company
- Ethnograph
- Microsoft Access
- Netlytic program
- Open code
- Python
- SAS
- Symplur
- Algorithmia
- Altmetric
- Anthropac
- ArcGIS
- AutoML
- Concept System Core
- CSIRO ESA Platform
- Datamuse API
- Google sheet
- Google translate
- HyperRESEARCH
- IBM Watson Alchemy Language Tool
- INCLEN Qualitative Data Analysis Software (IQDAS)
- IRaMuTeQ software
- Limesurvey Database
- Linguistic Word Count software
- Matlab
- MCM Analyst
- Meltwater
- MIRO
- Ncapture tool
- NetDraw
- PAST
- Qualitrics
- Quirkos
- REDCap
- Scikit-learn
- Spreadsheet software (unnamed)
- Stata
- STTM - java software for topic modelling
- Taguette
- UCINET
- Webometric
- Wordle.net
- Perl
- RSS feed scraping
- Unnamed text-mining software
- UCINET6
